# Electrochemical Copper Catalysis: A Triple Catalytic System for Transient C(sp^2^)–H Functionalization through Mediated Electrolysis

**DOI:** 10.1021/acselectrochem.5c00233

**Published:** 2025-07-23

**Authors:** Tsz-Kan Ma, Callum S. Begg, James A. Bull

**Affiliations:** Department of Chemistry, Molecular Sciences Research Hub, 4615 Imperial College London, White City Campus, Wood Lane, London W12 0BZ, United Kingdom

**Keywords:** Electrosynthesis, Transient Directing Group, Copper Catalysis, C−H Functionalization

## Abstract

The development of copper-catalyzed C–H functionalization processes is challenging due to the inefficiency of conventional chemical oxidants in regenerating the copper catalyst. This study details the development of a mediated electrosynthetic approach involving triple catalytic cycles in transient C–H functionalization to achieve efficient copper-catalyzed C­(sp^2^)–H sulfonylation of benzylamines with sodium sulfinate salts. The triple catalytic system consists of a copper organometallic cycle for C–H functionalization, an aldehyde transient directing group (TDG) as an organocatalyst for imine formation, and a ferrocenium salt as an electrocatalyst. This mediated electrolysis strategy addresses key challenges associated with copper electrochemical C–H activation, including irreversible copper electroplating at the cathode and undesired substrate oxidative degradation. Mechanistic studies, including monitoring the anode operating potential and cyclic voltammetry, provided valuable insights into the mediated electrolysis process and the copper ion reoxidation mechanism to support the mechanistic proposal. This mediated strategy provides a new avenue for developing more efficient copper catalyzed transient C–H functionalization processes enabled by synthetic electrochemistry.

## Graphical abstract

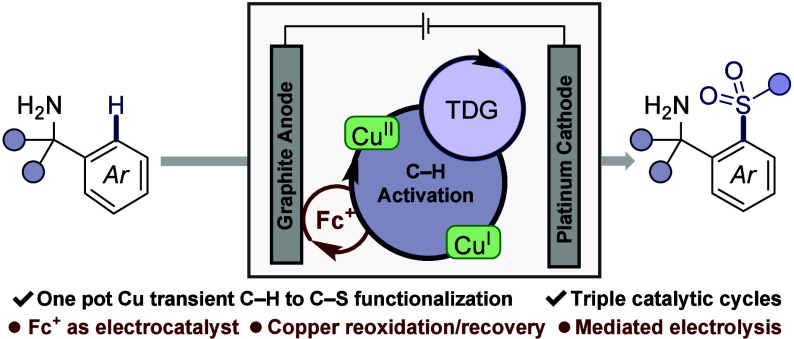

## Introduction

In the past decade, synthetic electrochemistry has gained renewed popularity as a sustainable and viable platform for organic synthesis.^1^ Oxidative electrochemical transformations coupled with the hydrogen evolution reaction (HER) can replace superstoichiometric quantities of chemical oxidants, making this approach highly attractive for developing sustainable synthetic methodologies.^2^ Recent developments in C–H functionalization have forged new pathways for preparing complex organic molecules by converting inert C–H bonds into valuable carbon–carbon or carbon–heteroatom bonds, which have significant implications for medicinal chemistry and materials science.^3–5^ Copper-mediated C–H functionalization can avoid the use of precious palladium catalysts but continue to face inefficiencies as large excesses of additional chemical oxidant or super-stoichiometric copper salts are often required.^6^ Therefore, an electrochemical approach with an earth-abundant and cheap copper catalyst for C–H functionalization would be a highly attractive solution to develop environmentally friendly synthetic protocols.

A complication in the development of efficient copper-catalyzed reactions in undivided electrochemical cells is the low reduction potential of ligandless copper ions (Cu­(II)/Cu­(I): *E*_onset_ −0.8 V vs Fc/Fc^+^ in MeCN), which leads to undesired electroplating of copper at the cathode.^7^ Consequently, copper-catalyzed electrochemical C–H functionalization reactions have been limited to the use of amide linked bidentate directing groups to provide strong coordination (Figure 1A).^8–13^ This copper ligation has been essential to achieve efficient copper catalysis, favoring the HER while minimizing undesired electroplating of the copper catalyst. Notably, Mei developed electrochemical copper catalyzed C­(sp^2^)–H amination using picolinamide as a bidentate directing group (Figure 1B).^8^

**Figure 1:**
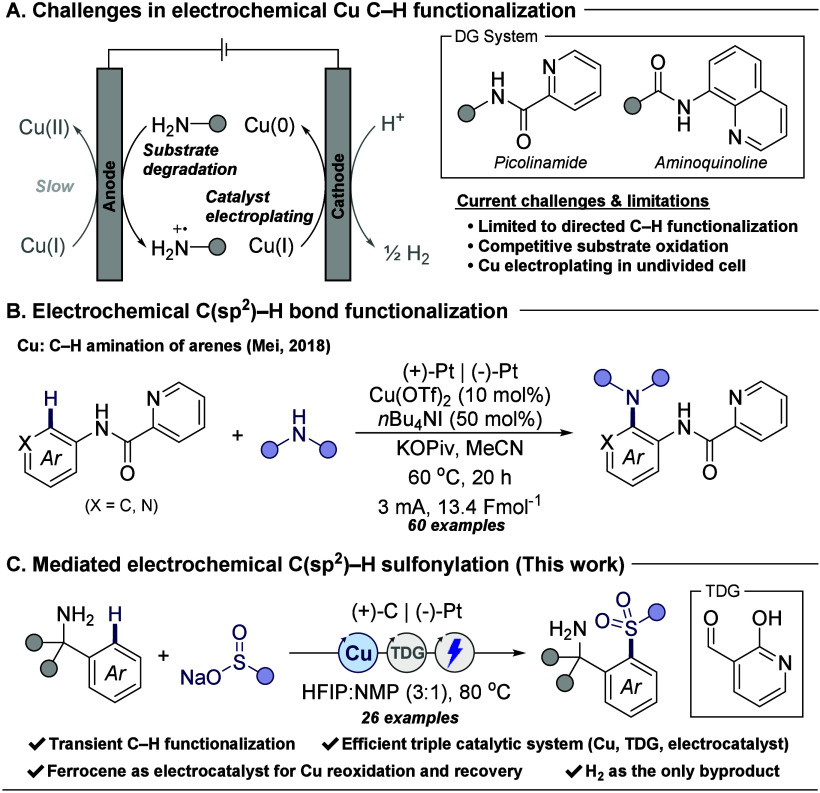
Challenges associated with copper electrosynthesis, previous work on metal catalyzed electrochemical C–H functionalization, and the triple catalytic system for electrochemical transient C­(sp^2^)–H sulfonylation of benzylamines.

The concept of transient directing groups is emerging in the field of C–H functionalization to improve step efficiency by removing additional steps required to install and cleave amide directing groups.^14^ A transient directing group (TDG) is an organocatalyst additive to react with common useful functionality on the substrate (aldehyde, ketone, or amine), often forming an imine as the true directing group for metalation. However, the dynamic nature of the TDG strategy cannot provide strong coordination to copper ions, and therefore, productive catalysis with direct electrolysis in a simple undivided cell is not feasible due to undesired electroplating at the cathode over HER.

Herein, we report a triple catalytic system overcoming the challenges associated with copper electrosynthesis for a transient C­(sp^2^)–H functionalization process. The introduction of an electrocatalyst is crucial in maintaining a high concentration of copper ions throughout electrolysis by reoxidizing Cu­(I) ions as well as recovering the electroplated elemental copper from the cathode. The catalytic system features an electrochemically driven, triple-interlocking catalytic cycle, incorporating a copper catalyst for C–H activation, an aldehyde TDG organocatalyst to form a bidentate imine directing group, and a ferrocenium salt as an electrocatalyst for catalyst recovery and regeneration (Figure 1C). Additionally, monitoring the anode operating potential in this mediated electrolysis process revealed the role of the ferrocene mediator in lowering the operating anodic potential during electrolysis to prevent substrate oxidative degradation.

## Results and Discussion

We previously reported the first example of transient directing group (TDG) catalyzed processes using copper salts to achieve C­(sp^2^)–H sulfonylation of benzaldehydes and benzylamines.^15–17^ However, a superstoichiometric amount of MnO_2_ (10 equiv) was required to achieve a reduction in the Cu loading to 50 mol %. Further reduction of copper loading led to a significant loss of efficiency. With this in mind, we aimed to establish a robust platform for the catalytic use of copper in transient C–H functionalization with the integration of synthetic electrochemistry, enhancing control over the oxidation process, removing chemical oxidants, and lowering copper loading.

### Reaction Optimization

Our early studies highlighted how the highly dynamic nature of the transient C–H functionalization process introduced challenges to the development of the electrochemical process. The ligandless copper catalyst was prone to undesired reduction at the cathode, leading to electroplating and termination of the productive reaction.^18^ Inspired by the elegant work of Sevov and co-workers on using a ferrocene mediator to promote copper-catalyzed anaerobic Chan–Lam coupling reactions, we initiated optimization studies on the sulfonylation of cumylamine **1** with sodium *p*-toluenesulfinate **2**, employing a mediated electrocatalysis strategy.^7^ A catalytic amount of 2-hydroxynicotinaldehyde was used as the TDG in the presence of 50 mol % of Cu­(OAc)_2_ and FcBF_4_ in HFIP at 80 °C in a sealed, undivided cell equipped with a graphite anode and platinum cathode under constant current electrolysis (*i* = 3 mA, *Q* = 4 Fmol^–1^). Early optimization using HFIP as the solvent resulted in less than 10% of the sulfonylation product **3**. Gratifyingly, changing the solvent system to a mixture of HFIP and NMP (3:1) dramatically improved the yield to 71% with 50 mol % of Cu­(OAc)_2_. In this solvent system, the catalyst loading was further reduced to 20 mol %, affording the desired sulfone in 67% yield. To systematically investigate the importance of different reaction parameters, a design of experiments (DoE) approach employing a definitive screening design was used to study six continuous parameters: the loading of amine, Cu­(OAc)_2_, FcBF_4_, TDG, K_2_CO_3_, and the ratio of NMP in HFIP (See SI for details).

Optimal conditions were identified with 1.5 equiv of amine, 15 mol % 2-hydroxynicotinaldehyde as TDG, and 2 equiv of K_2_CO_3_ as the base in a 3:1 mixture of HFIP:NMP (0.20 M) as the solvent without additional supporting electrolyte under constant current electrolysis (*i* = 3 mA, 1.4 mA/cm^2^, *Q* = 4 Fmol^–1^) with a graphite anode and platinum cathode (Table 1, entry 1). Under these conditions, the copper catalyst and electrocatalyst loadings could be lowered to 20 mol % to achieve a 77% yield of sulfonylation product **3**. A series of control experiments confirmed the essential role of TDG, FcBF_4_ as an electrocatalyst, base, heat, and electrochemistry (Table 1, entries 1–7). A low yield of 12% was observed in the absence of the TDG whereby the free amine provides a directing group (Table 1, entry 2). This is consistent with previous DFT calculations that demonstrate that the bidentate imine effectively lowers the energy barrier for C–H activation.^16^ The reaction was unsuccessful when HFIP or NMP was used as the sole solvent (Table 1, entries 8–9). A significant difference in the cell potential was observed when the reaction was carried out in HFIP (Figure S16, gray line, *E*_cell_ = 0.5 V) and in a mixture of HFIP:NMP (Figure S16, black line, *E*_cell_ = 1.5 V), presumably due to unproductive Fc/Fc^+^ recycling in the absence of NMP as co-solvent. Other HFIP solvent mixtures with DMF, DMSO, and MeCN were less effective compared to NMP (Table 1, entries 10–12). Alternative copper catalysts, such as CuOAc, Cu­(OTf)_2_, CuF_2_, and Cu­(TFA)_2_, were less effective than Cu­(OAc)_2_ (Table 1, entries 13–16). Additionally, different electrochemical parameters were investigated. The use of tetrabutylammonium tetrafluoroborate as an additional supporting electrolyte was unnecessary, as FcBF_4_ could serve a dual role as both the electrocatalyst and supporting electrolyte (Table 1, entry 17). Although the use of reticulated vitreous carbon (RVC) as the anode showed a slight improvement in yield, significant disintegration of the electrode was observed under the reaction conditions (Table 1, entry 18). The use of platinum as the cathode was essential to promote the desired hydrogen evolution reaction.^19^ Other cathode materials, such as nickel and stainless steel, which have a higher reduction potential for the hydrogen evolution reaction, showed diminished efficiency as significant copper electroplating was observed (Table 1, entry 19–20). A lower conversion, with a yield of 58%, was observed when the reaction was carried out under constant potential electrolysis with a three-electrode set up to reoxidize ferrocene selectively (Table 1, entry 21).

**Table 1:**
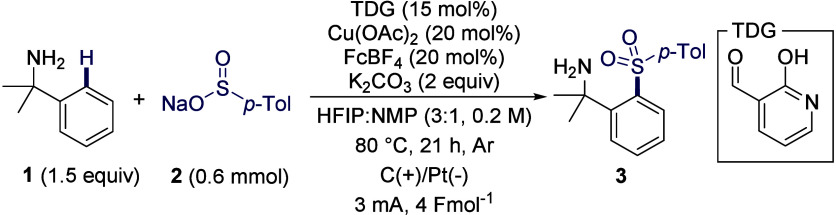
Optimization of Electrochemical Conditions^a^

Entry	Deviation from Standard Conditions	Yield^b^
**Control Experiments**		
**1**	**None**	**77% (74%)**
2	No TDG	12%
3	No Cu(OAc)_2_	0%
4	No FcBF_4_	13%
5	No K_2_CO_3_	14%
6	Room temperature	0%
7	No current	17%
**Solvent Systems**		
8	HFIP	9%
9	NMP	5%
10	HFIP:DMF (3:1)	43%
11	HFIP:DMSO (3:1)	54%
12	HFIP:MeCN (3:1)	18%
**Copper Catalysts**		
13	CuOAc	54%
14	Cu(OTf)_2_	62%
15	CuF_2_	59%
16	Cu(TFA)_2_	51%
**Electrochemical Parameters**		
17	With *n*-Bu_4_NBF_4_	73%
18	RVC(+)/Pt(−)	80%
19	C(+)/Ni(−)	28%
20	C(+)/SS(−)	26%
21	Constant potential electrolysis^c^	58%

a Reactions were conducted on a 0.60 mmol scale with respect to the sulfinate salt.

b Yield determined by analysis of crude ^1^H NMR using 1,3,5-trimethoxybenzene as internal standard. Isolated yield in parentheses.

c Constant potential electrolysis (+0.20 V vs Ag wire, 3.8 Fmol^–1^) was carried out in an undivided cell with Ag wire as the pseudo reference electrode for 21 h.

### Mechanistic Studies

To compare the performance of the electrochemical triple catalytic system, conventional chemical oxidants were evaluated under the reaction conditions (Figure 2A). The mediated electrolysis approach (75%) not only served as a cost-effective methodology with minimal chemical waste but also enabled efficient catalysis that outperformed those of the tested chemical oxidants. Excess FcBF_4_ alone was unable to achieve the same result (37%), likely because the regenerated Cu­(OAc)_2_ acted as an oxidant to non-productively reoxidize ferrocene, diminishing overall catalytic turnover. Other chemical oxidants (2 equiv), including AgOAc, MnO_2_, PhI­(OAc)_2_, and K_2_S_2_O_8_, were ineffective in turning over 20 mol % of copper catalyst, highlighting the unique advantage of the electrochemical approach.

**Figure 2:**
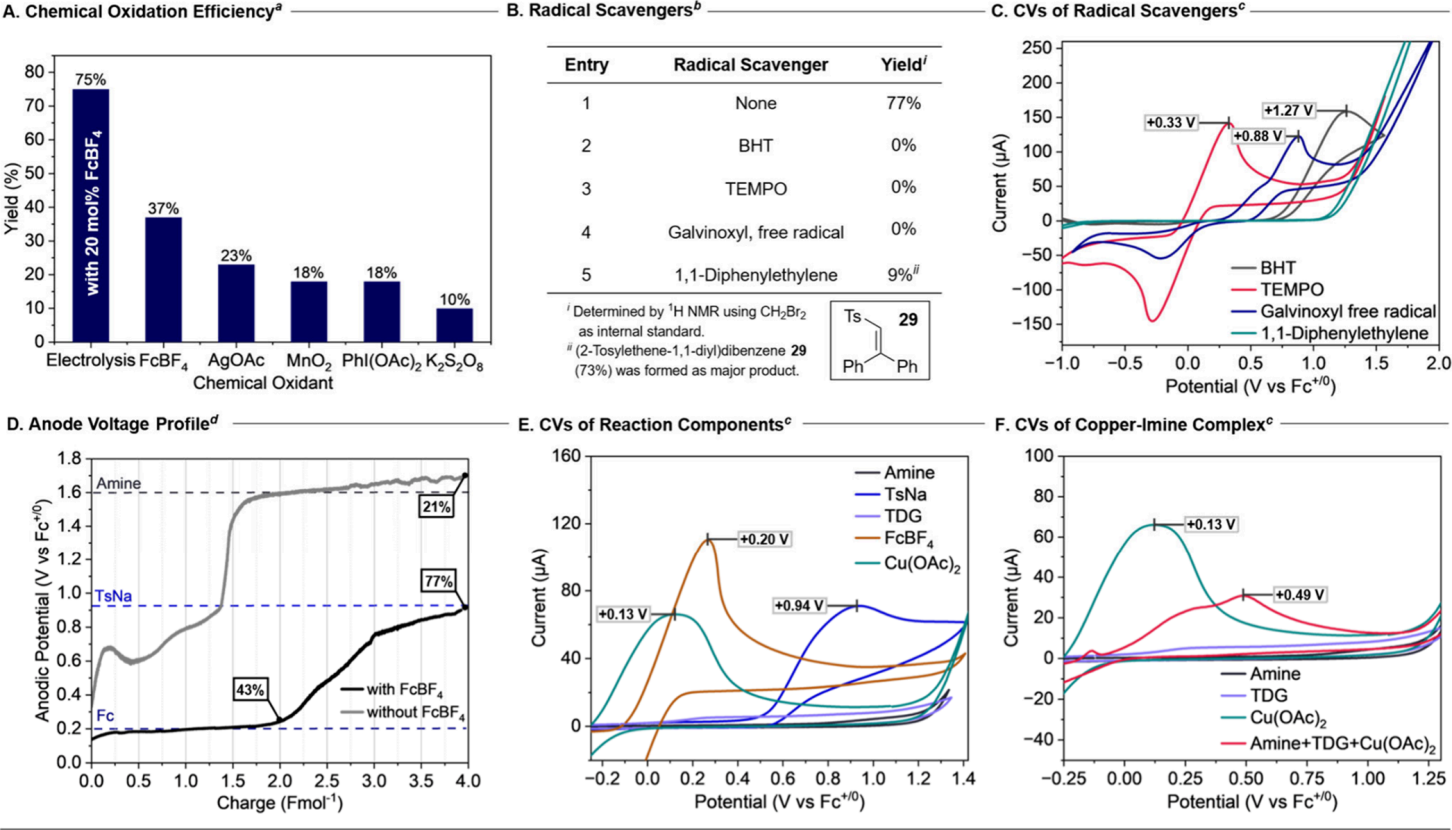
Mechanistic investigations. *^a^* Reaction between amine **1** and sulfinate salt **2** with 2 equiv of chemical oxidant for comparison. *^b^* Reaction between amine **1** and sulfinate salt **2** under standard electrochemical conditions with 1 equiv of radical scavenger. *^c^* CV conditions: 20 mM of analyte in 3:1 HFIP:NMP with 0.10 M *n*Bu_4_BF_4_ as electrolyte and 100 mVs^–1^ scan rate at room temperature using glassy carbon working electrode, Ag wire as reference electrode, and Pt wire counter electrode. Potentials were calibrated with Fc as an internal reference. ^*d*^Electrolysis was carried out with a 3 electrode undivided cell with Ag wire as reference electrode. Yield determined by analysis of crude ^1^H NMR with 1,3,5-trimethoxybenzene as an internal standard.

To investigate the reaction pathway and support the involvement of radical intermediates derived from the sulfinate salt, various radical scavengers (1 equiv) were introduced into the reaction (Figure 2B). The addition of butylated hydroxytoluene (BHT), TEMPO, or galvinoxyl free radical completely inhibited the sulfonylation reaction. When 1,1-diphenylethylene was utilized as the radical trap, a low yield of the desired sulfonylation product was observed (9%), along with the formation of (2-tosylethene-1,1-diyl)­dibenzene **29** (73%) as the major product, resulting from trapping the sulfonyl radical generated under the reaction conditions.^20^ Examination of the electrochemical properties of these radical scavengers by cyclic voltammetry showed that TEMPO (Figure 2C, red curve)^21^ and galvinoxyl free radical (Figure 2C, blue curve) could be oxidized by the anode under the reaction conditions while BHT (Figure 2C, grey curve) and 1,1-diphenylethylene (Figure 2C, green curve) remained electrochemically inert. These results provide direct evidence that the reaction proceeds through a radical pathway involving sulfonyl radicals.

Insight into the electrochemical sulfonylation mechanism was obtained by monitoring the operating potential of the anode throughout the reaction (Figure 2D). During the FcBF_4_ mediated process, the operating anodic potential was maintained at approximately +0.20 V (vs Fc^+/0^) during the first 2 Fmol^–1^ of charge transfer to produce the sulfone product in 43% yield (Figure 2D, black line), consistent with the regeneration of FcBF_4_ as the major electrochemical process (Figure 2E, brown curve). As the reaction progressed (2.0–4.0 Fmol^–1^), the operating anodic potential gradually increased from +0.20 V to +0.80 V (*E*_cell_ 1.5 V to 2.4 V), suggesting simultaneous sulfinate oxidation and FcBF_4_ regeneration in this phase of the reaction, to drive the reaction to completion to achieve 77% yield. In contrast, the unmediated process (Figure 2D, gray line) exhibited significantly higher operating anodic potentials from +0.80 V to +1.60 V (vs Fc^+/0^), corresponding to the direct anodic oxidation of the sulfinate salt (Figure 2E, blue curve) and amine substrate (Figure 2E, black curve). This led to a poor yield of 21% and a complex reaction profile, highlighting the essential role of the ferrocenium mediator. The mediator not only facilitates efficient homogeneous reoxidation of the copper catalyst but also prevents substrate degradation by maintaining the low anodic potential during electrolysis. Additionally, a control experiment replacing 20 mol % Cu­(OAc)_2_ with 20 mol % Cu powder under standard reaction conditions yielded the sulfone product in 82%, demonstrating that FcBF_4_ efficiently reoxidizes Cu(0), enabling the recovery of electroplated copper back to the active catalytic cycle under the mediated electrochemical conditions. Cyclic voltammetry (CV) studies provided further evidence of the involvement of a copper-imine complex. In the presence of amine and TDG, the copper­(II) catalyst exhibited a pronounced oxidative current at +0.49 V (vs Fc^+/0^) (Figure 2F, red curve, and Figure S9), suggesting the formation of a copper­(II)–imine complex. This copper­(II) complex could potentially be oxidized by the sulfonyl radical generated from the sulfinate salt (*E*_pa_ = +0.94 V vs Fc^+/0^) to the corresponding copper­(III) intermediate, facilitating product formation *via* reductive elimination.^22^ The CV of a mixture of CuOAc and FcBF_4_ indicated that the Fc wave becomes less reversible, suggesting its role in the reoxidation of CuOAc (Figure S8).

### Proposed Reaction Mechanism

Based on the above findings, previous mechanistic studies, and DFT calculations, the FcBF_4_-mediated, copper-catalyzed electrochemical transient C­(sp^2^)–H sulfonylation of benzylamines proceeds *via* triple interlocking catalytic cycles (Figure 3).^16^ The benzylamine substrate **A** enters the TDG-catalyzed organocatalytic cycle, condensing with 2-hydroxynicotinaldehyde to form an imine intermediate **B**, with H_2_O as a by-product. The resulting imine coordinates with the Cu­(OAc)_2_ catalyst to form copper–imine complex **C**, which undergoes a reversible concerted metalation deprotonation (CMD) step to generate cupracycle **D**. Radical addition between cupracycle **D** and sulfonyl radicals, generated *via* single-electron transfer (SET) between sodium sulfinate and Cu­(OAc)_2_, yields Cu­(III) intermediate **E**. Reductive elimination of intermediate **E** affords the sulfonylated imine product **F** and copper­(I) ions. Subsequent hydrolysis of the sulfonylated imine **F** by H_2_O regenerates TDG and provides the final sulfonylamine product **G**. Simultaneously, Cu­(I) ions are homogeneously reoxidized by ferrocenium ions, regenerating the active copper­(II) catalyst and ferrocene. Ferrocene undergoes anodic oxidation to regenerate ferrocenium ions, sustaining the electrocatalytic cycle. This ferrocene mediated process bypasses the challenge of slow electron-transfer kinetics for copper ion reoxidation to enable more efficient regeneration of the copper catalyst.^18^ Two distinct cathodic half-cell reactions are operational. The first involves the electroplating of Cu­(I) ions to elemental copper, which is subsequently reoxidized by freely diffusing ferrocenium ions to Cu­(I) and further to Cu­(II), ensuring continuity of the copper catalytic cycle. The second cathodic process involves the hydrogen evolution reaction (HER) of HFIP at the platinum cathode, providing the driving force for the reaction while generating molecular hydrogen as the sole by-product.

**Figure 3:**
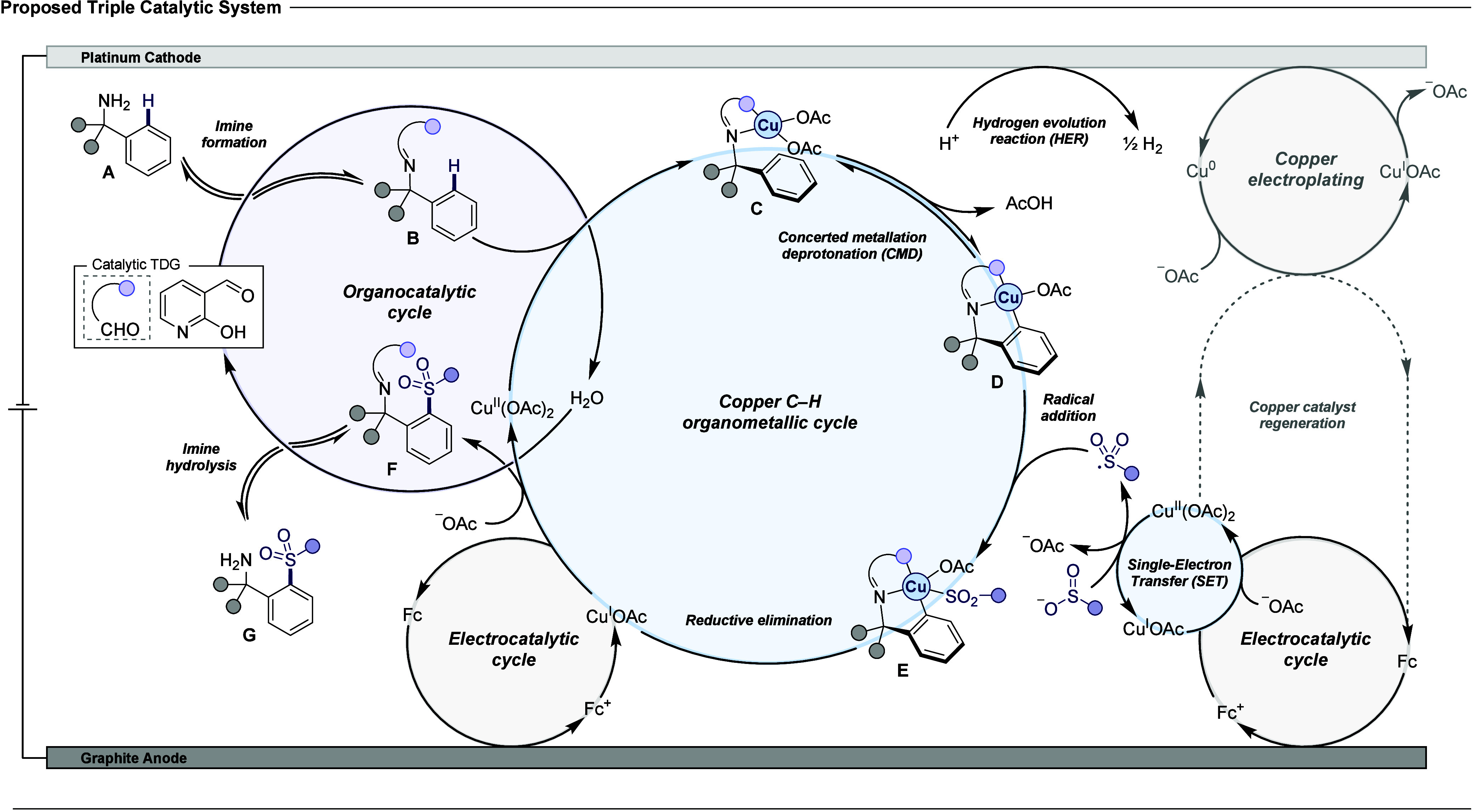
Proposed reaction mechanism with a triple interlocking catalytic system.

### Reaction Scope

With the optimal conditions in hand, the scope of this reaction was explored using various benzylamines and sodium sulfinate salts (Scheme 1). Benzylamines with an electron-donating group (OMe) at the *ortho*-, *meta*-, and *para*-positions were tested. The *meta*-substituted benzylamine afforded a 1:1 mixture of mono-sulfonylated products **5** in an excellent yield (77%). A slight decrease in yield was observed with *para*-substituted benzylamine **6** (53%), while a low yield was obtained with *ortho*-substituted benzylamine **4** (15%), most likely due to steric hindrance. 3,4,5-Trimethoxylbenzylamine **7** underwent sulfonylation successfully without difunctionalization, delivering the product in good yield (63%). Substrates containing benzylic protons, such as benzylamine and 1-phenylethan-1-amine, were found to be incompatible due to undesired benzylic oxidation.^23^ However, the benzylic position was not limited to a *gem*-dimethyl group; a fused ring system such as the cyclopentyl ring at the benzylic position was well tolerated, yielding the desired sulfone **8** in an excellent yield (80%). Halogenated benzylamines **9**–**11**, including those with F, Cl, or Br substituents, were also compatible, with no cross-coupling side products observed. Additionally, benzylamine with an electron-withdrawing group (CF_3_) at the *para*-position underwent sulfonylation successfully to produce sulfone **12** in an excellent yield (70%). Biaryl benzylamine **13** was also compatible under these reaction conditions. Notably, in all cases, no disulfonylation was observed. A wide range of sodium sulfinate salts was well-tolerated under the developed conditions. Sodium methylbenzenesulfinates with the methyl substitution at the *ortho*-, *meta*-, and *para*-positions were tested to investigate the steric effects on the sulfinate salts coupling partner. The highest yield was obtained when the methyl group was at the *para*-position of the arene, affording sulfone product **3** in an excellent yield (74%). A decreasing trend in yield was observed for the *meta*- (55%) and *ortho*-substituted (40%) arenes **14**–**15** due to increased steric hindrance. Reaction with sodium methanesulfinate in an undivided cell gave the corresponding sulfone **16** in a poor yield (11%), presumably due to oxidative degradation by FcBF_4_. However, a much higher yield (61%) was observed when the reaction was performed in a divided cell in the absence of FcBF_4_ under constant current electrolysis conditions (1.5 mA, 2 Fmol^–1^). Sodium cyclopropanesulfinate underwent sulfonylation successfully, providing desired product **17** in good yield (56%). The sulfinate salt derived from bicyclo[1.1.1]­pentane was also compatible, delivering sulfone product **18** in a moderate yield (38%). Halogenated sulfinate salts containing F, Cl, or Br substituents were well-tolerated under the electrochemical conditions to produce the corresponding amines **19**–**21** (46–64%). Sulfinate salts with varying electronic properties on the arene were also compatible. For example, sulfinate salts with electron neutral arene such as naphthalene and phenyl and *tert*-butylbenzene, afforded the corresponding sulfone products **22**, **23**, and **26** in moderate to good yield (30–53%). Sulfinate salt with an electron donating group (OMe) on the arene substituent gave desired product **24** in excellent yield (67%). Electron-withdrawing groups on the arene of the sulfinate salts, including *para*-CF_3_ **25** (52%), 3,4-dichloro **27** (60%), and 3,5-ditrifluoromethyl **28** groups (62%), were also well tolerated.

**Scheme 1:**
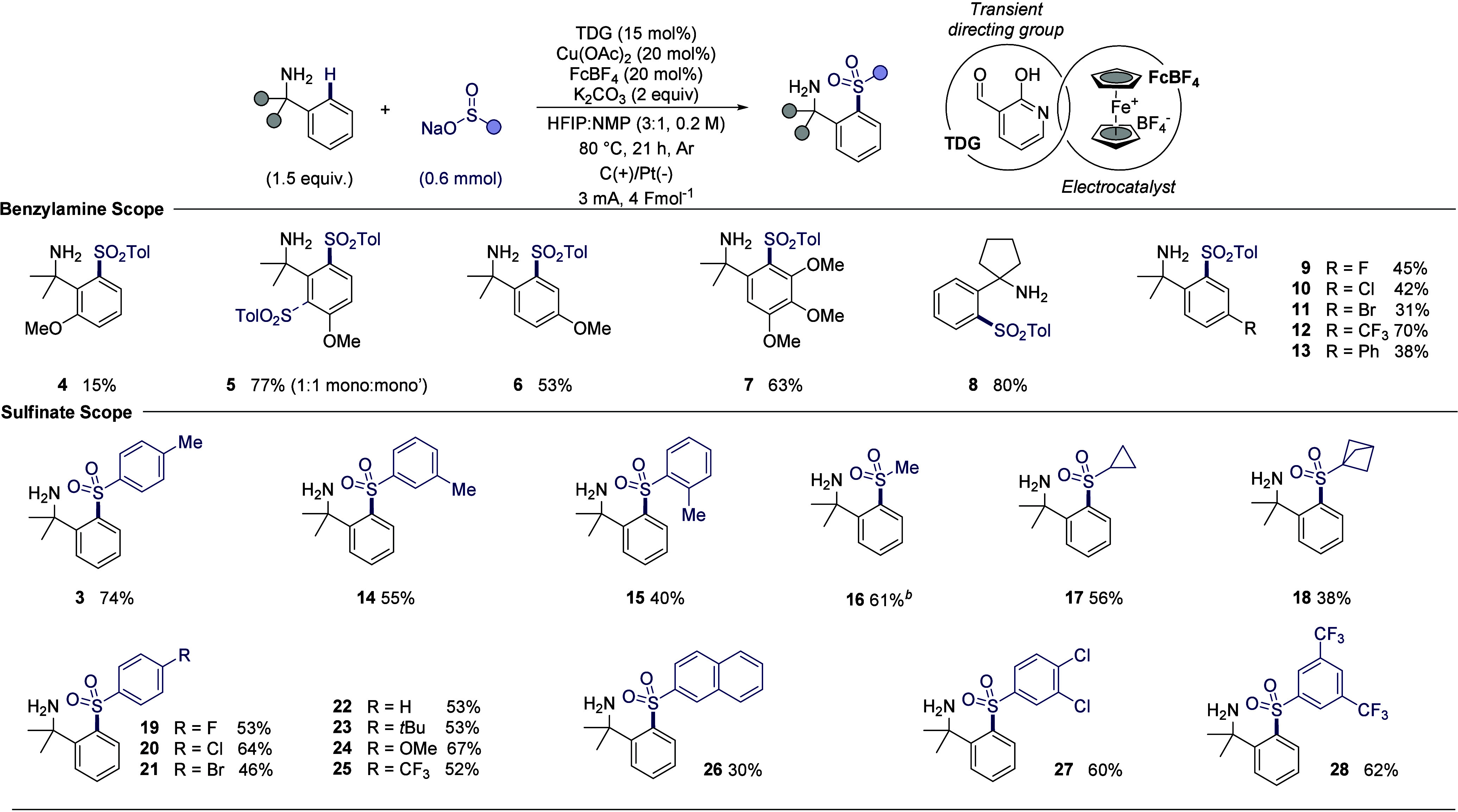
Scope of Electrochemical Transient C–H Sulfonylation of Benzylamines^a^

a Reactions were conducted on a 0.60 mmol scale with isolated yields reported.
b Electrolysis in a divided cell in the absence of FcBF_4_ (1.5 mA, 2 Fmol^–1^).

## Conclusion

In summary, an electrochemically driven triple catalytic system was developed for the efficient catalytic C­(sp^2^)–H sulfonylation of benzylamines with sulfinate salts. The mediated electrochemical approach enables efficient copper catalysis, which was unattainable with conventional chemical oxidants. The ferrocenium salt mediator is essential to maintaining a mild anodic potential of +0.20 V (vs Fc^+/0^) during electrolysis, preventing the undesired direct anodic oxidation of sodium sulfinate salts and benzylamine substrates. Furthermore, it enables efficient recovery of electroplated copper at the platinum cathode back to the active Cu­(II) catalyst, revealing the platinum surface for HER. This catalytic system effectively removes the need for precious metal catalysts, covalently bonded directing groups, and chemical oxidants, demonstrating the potential of efficient copper-catalyzed transient C–H functionalization with electrochemistry to achieve high levels of control and selectivity. We anticipate that this triple catalytic system will inspire further developments in copper-catalyzed transient C–H functionalization. Further studies on the applications of this electrochemical approach to different C–H to C–heteroatom bond formation processes are ongoing in our laboratory.

## Experimental Section

### General Electrochemical Sulfonylation Procedure

Amine (0.90 mmol), 2-hydroxynicotinaldehyde (11 mg, 0.09 mmol), Cu­(OAc)_2_ (22 mg, 0.12 mmol), K_2_CO_3_ (166 mg, 1.20 mmol), FcBF_4_ (33 mg, 0.12 mmol), and sodium sulfinate (0.60 mmol) were added to the reaction tube, and HFIP:NMP (3:1, 3 mL) was added. The reaction tube was sealed with a Suba-Seal and degassed by purging with Ar for 1 min with a vent needle. The reaction tube was then sealed with a PTFE cap fitted with graphite as the working electrode (submerged surface area of 2.16 cm^2^) and platinum as the counter electrode (submerged surface area of 1.05 cm^2^; 4 mm electrode distance). The resulting mixture was electrolyzed under constant current (3 mA, 4 Fmol^–1^) at 80 °C for 21 h with stirring at 800 rpm in an oil bath. The reaction mixture was allowed to cool to room temperature, diluted, and transferred to a separating funnel with EtOAc. The electrodes were submerged in EtOAc (9 × 5 mL) with stirring until the EtOAc remained colorless. The combined organic layer (about 50 mL in total) was washed with H_2_O (4 × 50 mL), dried over Na_2_SO_4_, filtered, and concentrated to give the crude reaction mixture as a dark brown oil. The crude material was diluted with EtOAc (50 mL) and extracted with aqueous HCl (1 M, 4 × 15 mL). The combined aqueous layers were basified with NaOH (2 M, 50 mL) to pH 14 and then extracted with CH_2_Cl_2_ (4 × 20 mL). The combined organic layers were dried over Na_2_SO_4_, filtered, and concentrated to give the crude material as a yellow oil and purified by column chromatography (EtOAc:MeOH 0 to 10%) to give the desired sulfonylated product.

## Supplementary Material



## Data Availability

The data underlying this study are available in the published article and in its Supporting Information and openly available in the Imperial College London Research Data Repository at 10.14469/hpc/15280.

## Notes

Experimental procedure, CV data, characterization data (^1^H, ^13^C, and ^19^F NMR, IR, HRMS, and X-ray crystal structures) (PDF)

## Notes

Deposition Numbers 2416954–2416958 contain the supplementary crystallographic data for this paper. These data can be obtained free of charge via the joint Cambridge Crystallographic Data Centre (CCDC) and Fachinformationszentrum Karlsruhe Access Structures service.
