# Supplementary material for: Electrochemical Copper Catalysis: A Triple Catalytic System for Transient C(sp2)–H Functionalization through Mediated Electrolysis
Source: ACS Electrochem. 2025 Jul 23;1(9):1863–70. doi: 10.1021/acselectrochem.5c00233 (PMC12415948; doi:10.1021/acselectrochem.5c00233)
Supplement: Supplementary file 1 [file ec5c00233_si_001.pdf]

SUPPORTING INFORMATION

Electrochemical Copper Catalysis: A Triple Catalytic System for Transient C(sp²)–H Functionalization Through Mediated Electrolysis

Supporting Information – Reaction Optimisation Data,
Cyclic Voltammograms, Experimental Procedures,
^{1}H and ^{13}C NMR Spectra

Tsz-Kan Ma,* Callum S. Begg and James A. Bull*

Department of Chemistry, Imperial College London, Molecular Sciences Research Hub, White City Campus, Wood Lane, London, W12 0BZ, England

*E-mail: j.bull@imperial.ac.uk, tsz.ma11@imperial.ac.uk

Contents

General Experimental S2
Reaction Optimisation S3
Solvent S3
DoE Optimisation: Definitive Screening Design S3
Control Reactions S6
Electrode Materials S7
Chemical Oxidants Comparison S7
Radical Scavenger S8
General Procedure for the Synthesis of Benzylamines S9
Synthesis of Sulfinate Salts S11
General Procedure: From Thiols S11
General Electrochemical Sulfonylation Procedure S13
Unsuccessful Substrates S14
Sulfonylation Reaction Scope: Varying Amines S15
Sulfonylation Reaction Scope: Varying Sulfinate Salts S19
Cyclic Voltammetry S24
Monitoring Anodic Potential S31
Monitoring Cell Potential S32
^{1}H and ^{13}C Spectra of Selected Compounds S33
X-Ray Crystallography S65
References S71

General Experimental

All reactions were run under an inert argon atmosphere using standard techniques unless otherwise stated. Electrochemical conditions were powered by TTi MX100QP quad output multi-range DC power supply unless otherwise stated. Cyclic voltammograms were recorded with Metrohm Autolab PGSTAT204 workstation. Anhydrous solvents (THF, diethyl ether, and MeOH) were obtained by filtration through drying columns. $Cu(OAc)_2$ (95%, product code: F325218) and 2-oxo-1,2-dihydro-3-pyridinecarbaldehyde (95%, product code: 044084) were obtained from Fluorochem and used as provided. Potassium carbonate (99.5%, product code: 024862) was obtained from Fluorochem and oven dried overnight prior to use. All other commercial reagents were used as supplied or purified by standard techniques where necessary. 1-Phenylcyclopentan-1-amine was prepared according to the procedure reported by Lee and co-workers.[1] 2-(3-Methoxyphenyl)propan-2-amine was synthesized according to the protocol described by Bull and co-workers.[2] 2-(4-(Trifluoromethyl)phenyl)propan-2-amine was prepared according to the procedure reported by Gregg and co-workers.[3]

Flash column chromatography was performed using 230-400 mesh silica with the indicated solvent system according to standard techniques with Biotage® Selekt System. Analytical thin-layer chromatography (TLC) was performed on precoated, glass-backed silica gel plates. Visualisation of the developed chromatogram was performed by UV absorbance (254 nm), aqueous potassium permanganate, *p*-anisaldehyde, phosphomolybdic acid or vanillin.

Infrared spectra (n_{max}, FTIR ATR) were obtained using an Agilent Technologies Cary 630 FTIR or a Perkin Elmer Spectrum 100 FTIR Spectrometer and recorded in reciprocal centimeters (cm^{-1}). Nuclear magnetic resonance spectra were recorded on either 400 or 500 MHz Bruker AvIII HD spectrometers with a SampleXpress automatic sample changer. The frequency used to record the NMR spectra is given in each assignment and spectrum (^{1}H NMR at 400 or 500 MHz; ^{13}C NMR at 101 MHz or 126 MHz; ^{19}F NMR at 377 MHz). Chemical shifts for ^{1}H NMR spectra are recorded in parts per million from tetramethylsilane with the solvent resonance as the internal standard (chloroform: δ = 7.27 ppm, dimethyl sulfoxide: δ = 2.50). Data is reported as follows: chemical shift [multiplicity (s = singlet, d = doublet, t = triplet, q = quartet, pent = pentet, m = multiplet and b = broad), coupling constant in Hz, integration, assignment]. ^{13}C NMR spectra were recorded with complete proton decoupling. Chemical shifts are reported in parts per million from tetramethylsilane with the solvent resonance as the internal standard (chloroform: δ = 77.0 ppm). *J* values are reported in Hz. ^{19}F NMR spectra were recorded with proton decoupling and indirectly referenced to $CFCl_3$ automatically via direct measurement of the absolute frequency of the deuterium lock signal by the spectrometer hardware. Melting points are uncorrected.

The high-resolution mass spectrometry (HRMS) analyses were performed using electrospray ion source (ESI) or pneumatically assisted atmospheric pressure chemical ionization (APCI) using an atmospheric solids analysis probe (ASAP). ESI was performed using a Waters LCT Premier equipped with an ESI source operated in positive or negative ion mode. The software used was MassLynx 4.1. This software does not account for the electron and all the calibrations/references are calculated accordingly, i.e. $[M+H]^+$ is detected and the mass is calibrated to output [M+H]. APCI was performed using an Orbitrap XL or Xevo G2S using an ASAP to insert samples into the APCI source. The sample was introduced at ambient temperature and the temperature increased until the sample vaporized.

Reaction Optimisation

Solvent

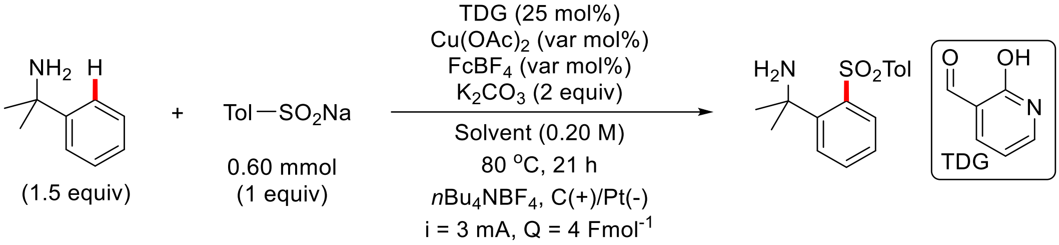

Entry	$Cu(OAc)_2$	$FcBF_4$	Solvent	Yield
1	50 mol%	50 mol%	HFIP	4%
**2**	**50 mol%**	**50 mol%**	**HFIP:NMP (3:1)**	**71% (72%)[a]**
**3**	**20 mol%**	**40 mol%**	**HFIP:NMP (3:1)**	**67%**

Yields determined by ^1H NMR spectroscopy using 1,3,5-trimethoxybenzene as an internal standard.
[a]Isolated yield after acid-base workup.

Table S1. Solvent Effect.

The addition of NMP as co-solvent was found to be an important factor, and the Cu loading can be reduced to 20 mol%.

DoE Optimisation: Definitive Screening Design

A 13 runs definitive screen was carried out with JMP for the electrochemical sulfonylation reaction to identify the most important factors. Below are the factors and range that were examined:

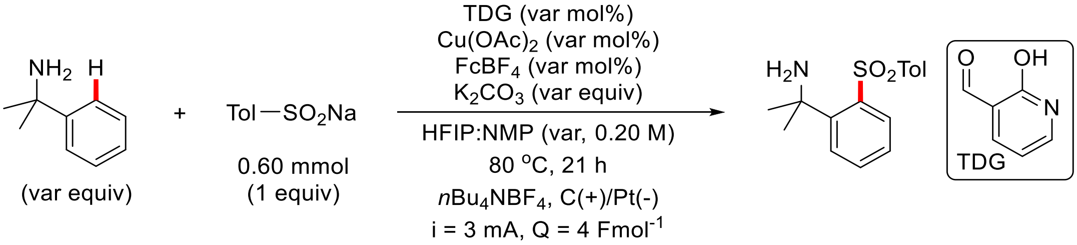

Factor	Range
Amine	1.5 – 2.0 equiv
$Cu(OAc)_2$	10 – 20 mol%
$FcBF_4$	20 – 40 mol%
TDG	15 – 35 mol%
K_2CO_3	2 – 3 equiv
NMP Ratio in HFIP	10 – 40%

Table S2. Variable parameters for DoE optimisation.

The following reaction parameters are fixed:

Factor	Range
TsNa	1.0 equiv (0.60 mmol)
Concentration	0.20 M
Temperature	80°C
$n\mathrm{Bu_4NBF_4}$	0.10 M
Anode	Graphite
Cathode	Platinum
Current and charge	i = 3 mA, Q = 4 $Fmol^{-1}$

Table S3. Fixed parameters for DoE optimisation.

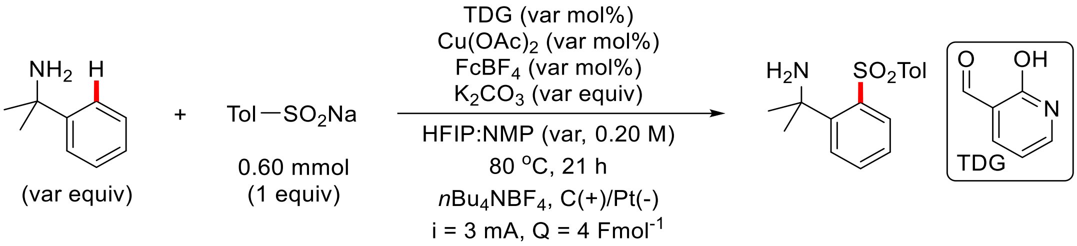

Entry	Amine (equiv)	$Cu(OAc)_2$ (mol%)	$FcBF_4$ (mol%)	TDG (mol%)	K_2CO_3 (equiv)	NMP (%)	Yield (%)
1	1.50	10	40	25	3.0	10	12
2	1.75	20	40	35	3.0	40	13
3	1.75	10	20	15	2.0	10	4
4	2.00	15	20	35	3.0	10	23
5	1.75	15	30	25	2.5	25	50
6	2.00	10	40	35	2.0	25	29
7	1.50	15	40	15	2.0	40	18
8	2.00	10	30	15	3.0	40	21
9	2.00	20	40	15	2.5	10	16
10	1.50	20	30	35	2.0	10	30
11	1.50	20	20	15	3.0	25	59
12	1.50	10	20	35	2.5	40	8
13	2.00	20	20	25	2.0	40	27

Yields determined by ^1H NMR spectroscopy using 1,3,5-trimethoxybenzene as an internal standard.

Table S4. Electrochemical sulfonylation DoE data.

Actual by Predicted Plot

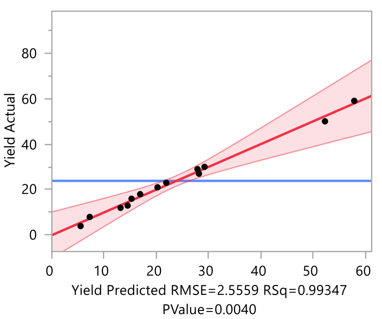

Effect Summary

Source	Logworth	PValue	
NMP Ratio in HFIP*NMP Ratio in HFIP	3.464	0.00034	
Cu(OAc)2(10,20)	2.508	0.00311	
Cu(OAc)2*K2CO3	2.325	0.00474	
FcBF4(20,40)	1.576	0.02654	
FcBF4*NMP Ratio in HFIP	1.471	0.03380	
K2CO3(2,3)	1.047	0.08971	^
TDG(15,35)	0.795	0.16048	
Amine(1.5,2)	0.574	0.26674	
NMP Ratio in HFIP(10,40)	0.086	0.82053	^

Studentized Residuals

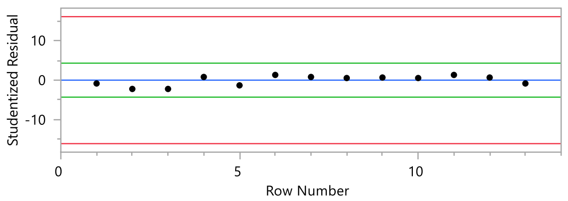

Residual by Predicted Plot

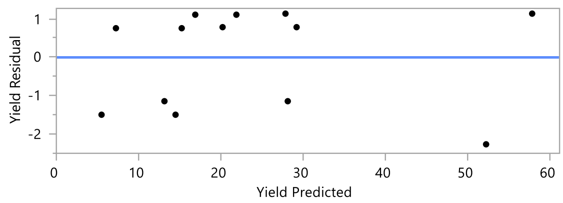

Externally studentized residuals with 95% simultaneous limits (Bonferroni) in red, individual limits in green.

Parameter Estimates

Term	Estimate	Std Error	t Ratio	Prob>|t|	Uncoded Estimate
Intercept	52.265625	1.69056	30.92	<.0001 *	-181.249
Cu(OAc)2(10,20)	7.1	0.808242	8.78	0.0031 *	10.818438
FcBF4(20,40)	-3.3	0.808242	-4.08	0.0265 *	-1.009036
K2CO3(2,3)	2	0.808242	2.47	0.0897	60.390625
NMP Ratio in HFIP(10,40)	0.2	0.808242	0.25	0.8205	7.408559
Cu(OAc)2*K2CO3	-9.398438	1.237363	-7.60	0.0047 *	-3.759375
FcBF4*NMP Ratio in HFIP	4.0742188	1.095142	3.72	0.0338 *	0.0271615
NMP Ratio in HFIP*NMP Ratio in HFIP	-36.94531	1.995188	-18.52	0.0003 *	-0.164201
Amine(1.5,2)	-1.1	0.808242	-1.36	0.2667	-4.4
TDG(15,35)	-1.5	0.808242	-1.86	0.1605	-0.15

Effect Tests

Source	Nparm	DF	Sum of Squares	F Ratio	Prob > F
Cu(OAc)2(10,20)	1	1	504.1000	77.1674	0.0031 *
FcBF4(20,40)	1	1	108.9000	16.6704	0.0265 *
K2CO3(2,3)	1	1	40.0000	6.1232	0.0897
NMP Ratio in HFIP(10,40)	1	1	0.4000	0.0612	0.8205
Cu(OAc)2*K2CO3	1	1	376.8773	57.6922	0.0047 *
FcBF4*NMP Ratio in HFIP	1	1	90.4130	13.8404	0.0338 *
NMP Ratio in HFIP*NMP Ratio in HFIP	1	1	2239.9280	342.8871	0.0003 *
Amine(1.5,2)	1	1	12.1000	1.8523	0.2667
TDG(15,35)	1	1	22.5000	3.4443	0.1605

Prediction Profiler

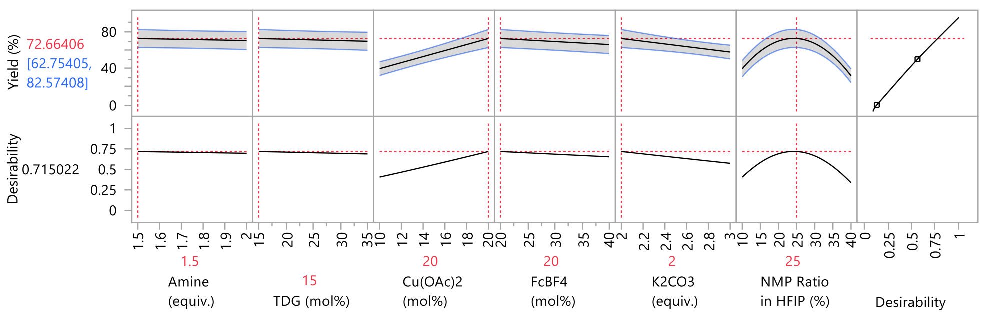

Figure S1. DSD Model with predicted optimal conditions.

The optimal conditions were tested, and the experimental result was 73% yield, which is in excellent agreement with the yield predicted by the DoE model.

Control Reactions

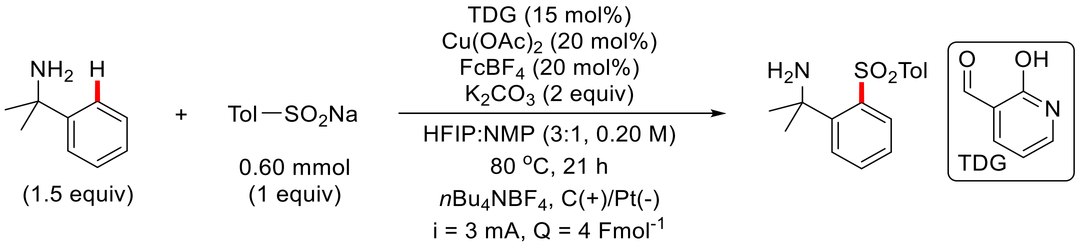

Entry	Deviation from standard conditions	Yield
1	No TDG	12%
2	No $Cu(OAc)_2$	0%
3	No $FcBF_4$	13%
4	No K_2CO_3	14%
5	HFIP as solvent	9%
6	NMP as solvent	5%
7	HFIP:DMF (3:1) as solvent	43%
8	HFIP:DMSO (3:1) as solvent	54%
9	HFIP:MeCN (3:1) as solvent	18%
10	CuOAc as catalyst	54%
11	$Cu(OTf)_2$ as catalyst	62%
12	CuF_2 as catalyst	59%
13	$Cu(O_2CCF_3)_2$ as catalyst	51%
**14**	**Cu powder as catalyst**	**82%**
**15**	**No *n*Bu_4NBF_4**	**75%**
16	No current	17%
17	Room temperature	0%

Yields determined by ^1H NMR spectroscopy using 1,3,5-trimethoxybenzene as an internal standard.

Table S5. Control experiments.

Electrode Materials

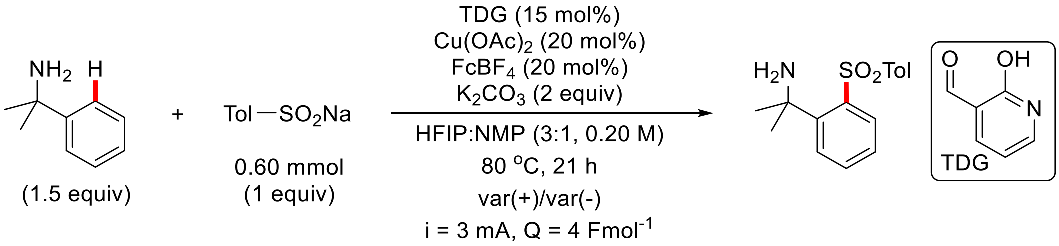

Entry	Anode	Cathode	Yield
**1**	**Graphite**	**Platinum**	**75%**
**2**	**RVC**	**Platinum**	**80%**
3	Graphite	Ni Plate	13%
4	Graphite	Ni Foam	28%
5	Graphite	Stainless Steel	26%

Yields determined by ^1H NMR spectroscopy using 1,3,5-trimethoxybenzene as an internal standard.

Table S6. Effect of different electrode materials.

Both graphite and RVS are suitable anode materials for this reaction, however, it was observed that the RVC electrode is too fragile under the reaction conditions and cannot be reused multiple times. Therefore, the graphite plate was chosen as the anode material for better reusability.

The platinum cathode was essential for this reaction to promote the hydrogen evolution reaction (HER) as the counter half-cell reaction. Other cathode materials examined (Ni and stainless steel) promoted the electroplating of copper, leading to a lower conversion of the desired reaction.

Chemical Oxidants Comparison

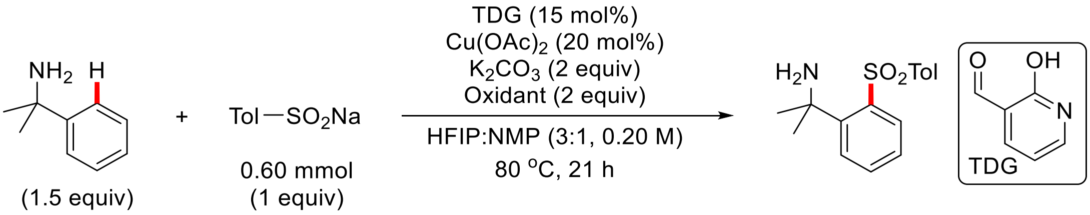

Entry	Oxidant (2 equiv.)	Yield
**1**	**Anodic oxidation with 20 mol% $FcBF_4$**	**75%**
2	MnO_2	18%
3	$K_2S_2O_8$	10%
4	$PhI(OAc)_2$	18%
5	AgOAc	23%
6	$FcBF_4$	37%

Yields determined by ^1H NMR spectroscopy using 1,3,5-trimethoxybenzene as an internal standard.

Table S7. Comparing chemical oxidants with electrochemical method.

A range of chemical oxidants was tested, and results have shown that anodic oxidation with a catalytic amount of $FcBF_4$ gave the best yield compared to chemical oxidants (**Table S7**).

Radical Scavenger

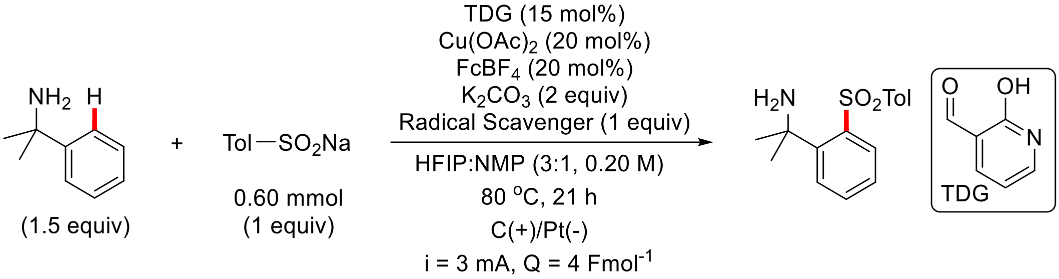

Entry	Radical Scavenger (1 equiv)	Yield
**1**	**None**	**77%**
2	BHT	0%
3	TEMPO	0%
4	Galvinoxyl, free radical	0%
5	1,1-Diphenylethylene	9%[a]

Yields determined by ^{1}H NMR spectroscopy using CH_2Br_2 as an internal standard.

Table S8. Trapping sulfonyl radicals with radical scavengers.

The addition of BHT, TEMPO and galvinoxyl free radial completely inhibited the reaction (**Table S8**). When 1,1-diphenyl ethylene was used as the radical trap, the desired sulfone product was formed in 9% yield, along with the formation of (2-tosylethene-1,1-diyl)dibenzene as the major product in 73% yield. These results support the reaction proceed via the radical pathway with sulfonyl radicals.

General Procedure for the Synthesis of Benzylamines

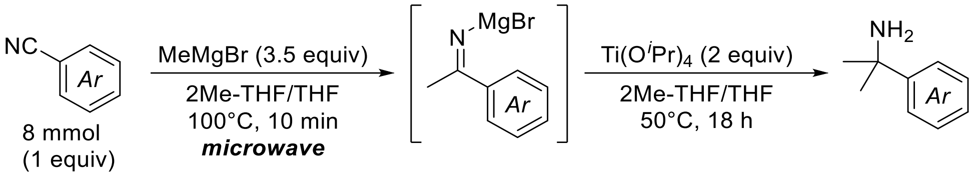

The protocol was modified from the reported procedure by Gregg and co-workers and performed on 8 mmol scale (2 mmol per microwave vial for 4 vials in total).[3] To each 20 mL Biotage microwave vial, MeMgBr (2.2 mL, 3.2 M in Me-THF, 7 mmol, 3.5 equiv) was added dropwise to a solution of nitrile (2 mmol) in THF (8 mL) and the resulting reaction mixture was heated at 100 °C for 10 min under microwave conditions. The reaction was allowed to cool down to room temperature and then diluted with THF (5 mL). $Ti(O^iPr)_4$ (0.6 mL, 4 mmol) was added, and the resulting mixture was heated at 50 °C for 18 h. The reaction mixture was allowed to cool to room temperature and the pressure was released with a short needle. The reaction was quenched with dropwise addition of brine (5 mL) and the resulting mixture was stirred for 30 min until a colour change from deep blue to yellow was observed. All the reaction mixture from 4 vials was transferred to a separating funnel with CH_2Cl_2 (200 mL in total). The organic layer was washed with brine (3 × 50 mL) and then dried over Na_2SO_4, filtered, and concentrated to afford the desired crude amine. The crude amine was dissolved in Et_2O (200 mL) with stirring and HCl in Et_2O (2 M; 5 mL) was added to precipitate out the amine HCl salt. The amine HCl salt was filtered and washed with Et_2O (100 mL), which was then dissolved in H_2O (50 mL). The aqueous layer was basified with aqueous NaOH (1 M; 20 mL) and extracted with CH_2Cl_2 (3 × 50 mL). The combined organic layer was dried over Na_2SO_4, filtered, and concentrated to give the pure benzylamine product.

2-(4-Methoxyphenyl)propan-2-amine

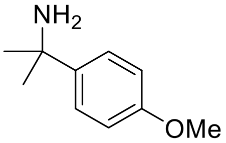

Prepared according to the general procedure using 4-methoxybenzonitrile (1.06 g, 8 mmol) and isolated the benzylamine (604 mg, 3.66 mmol, 46%) as yellow oil. **^{1}H NMR** (400 MHz, $CDCl_3$) δ 7.44 (d, *J* = 8.9 Hz, 1H, 2 × Ar-CH), 6.87 (d, *J* = 8.9 Hz, 1H, 2 × Ar-CH), 3.81 (s, 3H, OCH_3), 1.58 (s, 2H, NH_2), 1.49 (s, 6H, $C(CH_3)_2$). The analytical data (^{1}H, NMR) are in agreement with the reported literature.[4]

2-(3,4,5-Trimethoxyphenyl)propan-2-amine

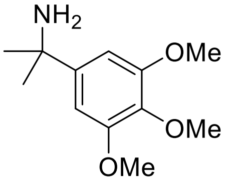

Prepared according to the general procedure using 3,4,5-trimethoxybenzonitrile (1.54 g, 8 mmol) and isolated the benzylamine (862 mg, 3.82 mmol, 48%) as yellow oil. **^{1}H NMR** (400 MHz, $CDCl_3$) δ 6.74 (s, 2H, 2 × Ar-CH), 3.89 (s, 6H, 2 × OCH_3), 3.84 (s, 3H, OCH_3), 1.64 (s, 2H, NH_2), 1.50 (s, 6H, $C(CH_3)_2$). **^{13}C NMR** (101 MHz, $CDCl_3$) δ 152.8 (2 × Ar-C_q), 146.1 (Ar-C_q), 136.3 (Ar-C_q), 102.2 (2 × Ar-CH), 60.8 (OCH_3), 56.1 (2 × OCH_3), 52.6 (C_qNH_2), 33.1 ($C(CH_3)_2$). **IR** (film)/cm^{-1} 2961, 2934, 2832, 1585, 1502, 1409, 1331, 1124, 1008. **HRMS** (TOF—ESI$^+$) m/z calcd. for $C_{12}H_{17}O_3^+$ [M–NH_3]$^+$: 209.1178; found 209.1181.

2-(4-Fluorophenyl)propan-2-amine

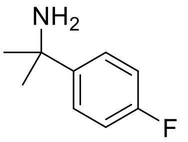

Prepared according to the general procedure using 4-fluorobenzonitrile (968 mg, 8 mmol) and isolated the benzylamine (874 mg, 5.70 mmol, 71%) as pale orange oil. **^{1}H NMR** (400 MHz, $CDCl_3$) δ 7.52 – 7.44 (m, 2H, 2 × Ar-CH), 7.05 – 6.96 (m, 2H, 2 × Ar-CH), 1.56 (s, 2H, NH_2), 1.49 (s, 6H, $C(CH_3)_2$). The analytical data (^{1}H, NMR) are in agreement with the reported literature.[5]

2-(4-Chlorophenyl)propan-2-amine

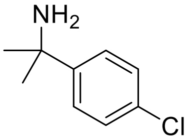

Prepared according to the general procedure using 4-chlorobenzonitrile (1.10 g, 8 mmol) and isolated the benzylamine (1.02 g, 6.00 mmol, 75%) as pale orange oil. **^{1}H NMR** (400 MHz, $CDCl_3$) δ 7.45 (d, *J* = 8.7 Hz, 2H, 2 × Ar-CH), 7.29 (d, *J* = 8.7 Hz, 2H, 2 × Ar-CH), 1.56 (s, 2H, NH_2), 1.48 (s, 6H, $C(CH_3)_2$). The analytical data (^{1}H, NMR) are in agreement with the reported literature.[5]

2-(4-Bromophenyl)propan-2-amine

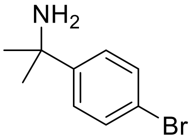

Prepared according to the general procedure using 4-bromobenzonitrile (1.46 g, 8 mmol) and isolated the benzylamine (900 mg, 4.20 mmol, 53%) as pale orange oil. **^{1}H NMR** (400 MHz, $CDCl_3$) δ 7.45 (d, *J* = 8.7 Hz, 2H, 2 × Ar-CH), 7.40 (d, *J* = 8.7 Hz, 2H, 2 × Ar-CH), 1.58 (s, 2H, NH_2), 1.48 (s, 6H, $C(CH_3)_2$). The analytical data (^{1}H, NMR) are in agreement with the reported literature.[2]

2-([1,1'-Biphenyl]-4-yl)propan-2-amine

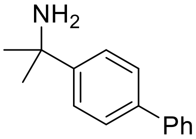

Prepared according to the general procedure using [1,1'-biphenyl]-4-carbonitrile (1.43 g, 8 mmol) and isolated the benzylamine (770 mg, 3.64 mmol, 45%) as yellow solid. **^{1}H NMR** (400 MHz, $CDCl_3$) δ 7.64 – 7.55 (m, 6H, 6 × Ar-CH), 7.45 (t, *J* = 7.6 Hz, 2H, 2 × Ar-CH), 7.35 (t, *J* = 7.3 Hz, 1H, Ar-CH), 1.63 (s, 2H, NH_2), 1.55 (s, 6H, $C(CH_3)_2$). The analytical data (^{1}H, NMR) are in agreement with the reported literature.[6]

Synthesis of Sulfinate Salts

General Procedure: From Thiols

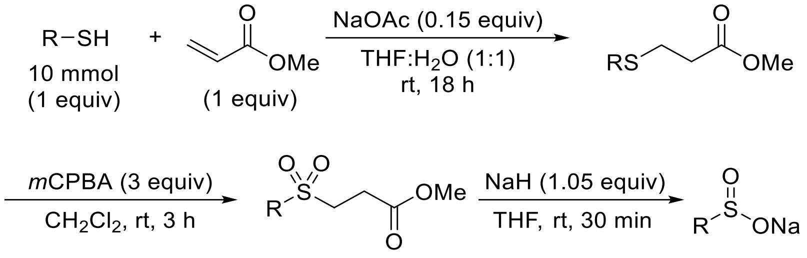

Step 1: Thia-Michael Addition
Sodium acetate (123 mg, 1.5 mmol, 0.15 equiv) and thiol (10 mmol, 1 equiv) were added sequentially to a solution of methyl acrylate (0.9 mL, 10 mmol, 1 equiv) in THF:H_2O (1:1, 34 mL), and the resulting reaction mixture was stirred for 18 h at room temperature. The reaction mixture was concentrated under reduced pressure and diluted with CH_2Cl_2 (50 mL) and H_2O (50 mL). Phases were separated and the aqueous layer was extracted with CH_2Cl_2 (3 × 50 mL). The combined organic layers were dried over Na_2SO_4, filtered, and concentrated to give the desired sulfide for subsequent steps without further purification.

Step 2: *m*CPBA Oxidation
*m*CPBA (6.72 g, 30 mmol, 3 equiv) was added to a solution of sulfide in CH_2Cl_2 (200 mL) and the resulting mixture was stirred for 3 h at room temperature. The reaction was quenched by the addition of aqueous NaOH (1 M, 100 mL), and the phases were separated. The aqueous layer was extracted with CH_2Cl_2 (3 × 50 mL) and the combined organic layers were dried over Na_2SO_4, filtered, and concentrated to give the desired sulfone for subsequent steps without further purification.

Step 3: Sulfinate Salt Formation
Sodium hydride (420 mg, 10.5 mmol, 1.05 equiv) was added to a solution of sulfone in THF (60 mL) and the resulting mixture was stirred for 1 h at room temperature. MeOH (2 mL) was added dropwise to quench the reaction, and the precipitate was filtered and washed with THF (50 mL) and hexane (50 mL) to give the sulfinate salt as white powder.

Sodium 3-methylbenzenesulfinate

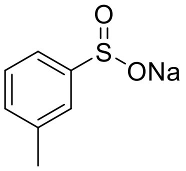

Prepared according to the general procedure using 3-methylbenzenethiol (1.24 g, 10 mmol) and isolated the sodium sulfinate salt (1.42 g, 7.98 mmol, 80%) as white powder. **^{1}H NMR** (400 MHz, $(CD_3)_2SO$) δ 7.29 – 7.21 (m, 2H, 2 × Ar-CH), 7.18 (t, *J* = 7.3 Hz, 1H, Ar-CH), 7.03 (d, J = 7.2 Hz, 1H, Ar-CH), 2.30 (s, 3H, Ar-CH_3).

Sodium 2-methylbenzenesulfinate

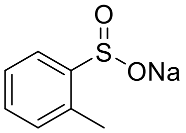

Prepared according to the general procedure using 2-methylbenzenethiol (1.24 g, 10 mmol) and isolated the sodium sulfinate salt (1.66 g, 9.32 mmol, 93%) as white powder. **^{1}H NMR** (400 MHz, $(CD_3)_2SO$) δ 7.62 (dd, *J* = 7.4, 1.7 Hz, 1H, Ar-CH), 7.20 – 7.07 (m, 2H, 2 × Ar-CH), 7.01 (d, J = 7.2 Hz, 1H, Ar-CH), 2.44 (s, 3H, Ar-CH_3).

Sodium 4-fluorobenzenesulfinate

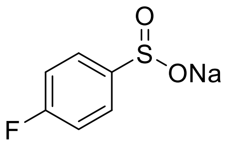

Prepared according to the general procedure using 4-fluorobenzenethiol (1.06 mL, 10 mmol) and isolated the sodium sulfinate salt (1.50 g, 8.25 mmol, 82%) as white powder. **^{1}H NMR** (400 MHz, $(CD_3)_2SO$) δ 7.46 (dd, *J* = 8.3, 6.0 Hz, 2H, 2 × Ar-CH), 7.10 (dd, *J* = 8.9 Hz, 2H, 2 × Ar-CH).

Sodium 4-chlorobenzenesulfinate

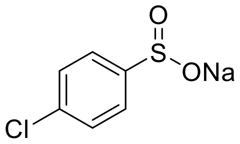

Prepared according to the general procedure using 4-chlorobenzenethiol (1.45 g, 10 mmol) and isolated the sodium sulfinate salt (1.85 g, 9.33 mmol, 93%) as white powder. **^{1}H NMR** (400 MHz, $(CD_3)_2SO$) δ 7.45 (d, *J* = 8.0 Hz, 2H, 2 × Ar-CH), 7.34 (d, *J* = 8.0 Hz, 2H, 2 × Ar-CH).

Sodium 4-bromobenzenesulfinate

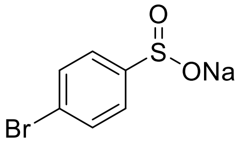

Prepared according to the general procedure using 4-bromobenzenethiol (1.89 g, 10 mmol) and isolated the sodium sulfinate salt (2.32 g, 9.54 mmol, 95%) as white powder. **^{1}H NMR** (400 MHz, $(CD_3)_2SO$) δ 7.48 (d, *J* = 8.0 Hz, 2H, 2 × Ar-CH), 7.39 (d, *J* = 8.2 Hz, 2H, 2 × Ar-CH).

Sodium naphthalene-2-sulfinate

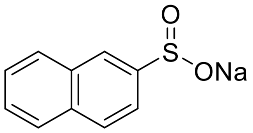

Prepared according to the general procedure using naphthalene-2-thiol (1.60 g, 10 mmol) and isolated the sodium sulfinate salt (1.71 g, 8.00 mmol, 80%) as white powder. **^{1}H NMR** (400 MHz, $(CD_3)_2SO$) δ 7.97 – 7.80 (m, 4H, 4 × Ar-CH), 7.65 (dd, *J* = 8.3, 1.5 Hz, 1H, Ar-CH), 7.51 – 7.43 (m, 2H, 2 × Ar-CH).

Sodium 4-methoxybenzenesulfinate

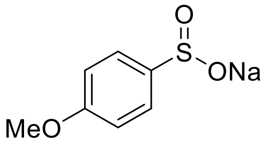

Prepared according to the general procedure using 4-methoxybenzenethiol (1.23 mL, 10 mmol) and isolated the sodium sulfinate salt (1.91 g, 9.82 mmol, 98%) as white powder. **^{1}H NMR** (400 MHz, $(CD_3)_2SO$) δ 7.34 (d, *J* = 8.8 Hz, 2H, 2 × Ar-CH), 6.84 (d, *J* = 8.8 Hz, 2H, 2 × Ar-CH), 3.73 (s, 3H, 2 × Ar-OMe).

Sodium 3,4-dichlorobenzenesulfinate

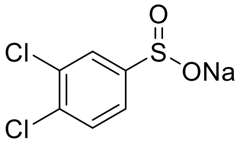

Prepared according to the general procedure using 3,4-dichlorobenzenethiol (1.79 g, 10 mmol) and isolated the sodium sulfinate salt (1.63 g, 7.00 mmol, 70%) as white powder. **^{1}H NMR** (400 MHz, $(CD_3)_2SO$) δ 7.59 – 7.53 (m, 2H, 2 × Ar-CH), 7.38 (dd, *J* = 8.0, 1.7 Hz, 1H, Ar-CH).

Sodium 3,5-bis(trifluoromethyl)benzenesulfinate

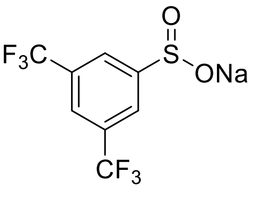

Prepared according to the general procedure using 3,5-bis(trifluoromethyl)benzenethiol (2.46 g, 10 mmol) and isolated the sodium sulfinate salt (2.41 g, 8.00 mmol, 80%) as white powder. **^{1}H NMR** (400 MHz, $(CD_3)_2SO$) δ 8.02 (s, 2H, 2 × Ar-CH), 7.97 (s, 1H, Ar-CH). **^{19}F NMR** (377 MHz,) δ -61.18.

General Electrochemical Sulfonylation Procedure

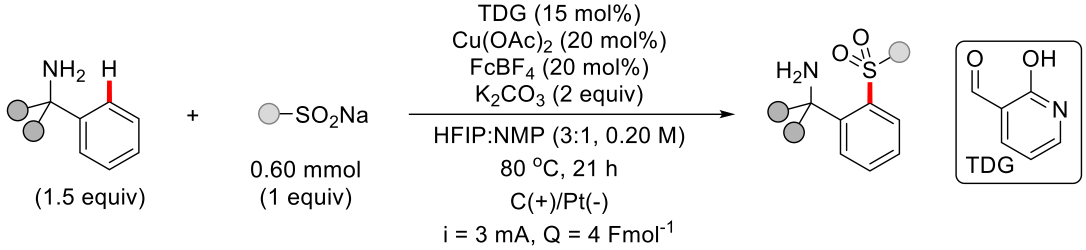

Amine (0.90 mmol), 2-hydroxynicotinaldehyde (11 mg, 0.09 mmol), $Cu(OAc)_2$ (22 mg, 0.12 mmol), K_2CO_3 (166 mg, 1.20 mmol), $FcBF_4$ (33 mg, 0.12 mmol), and sodium sulfinate (0.60 mmol) were added to the reaction tube and HFIP:NMP (3:1, 3 mL) was added. The reaction tube was sealed with a Suba-Seal and degassed by purging with Ar for 1 min with a vent needle. The reaction tube was then sealed with a PTFE cap fitted with graphite as the working electrode and platinum as counter electrode. The resulting mixture was electrolysed under constant current (3 mA, 4 $Fmol^{-1}$) at 80 °C for 21 h with stirring at 800 rpm in an oil bath. The reaction mixture was allowed to cool to room temperature, diluted and transferred to a separating funnel with EtOAc. The electrodes were submerged in EtOAc (9 × 5 mL) with stirring until the EtOAc remained colourless. The combined organic layer (about 50 mL in total) was washed with H_2O (4 × 50 mL), dried over Na_2SO_4, filtered, and concentrated to give the crude reaction mixture as a dark brown oil. The crude material was diluted with EtOAc (50 mL) and extracted with aqueous HCl (1 M, 4 × 15 mL). The combined aqueous layers were basified with NaOH (2 M, 50 mL) to pH 14 and then extracted with CH_2Cl_2 (4 × 20 mL). The combined organic layers were dried over Na_2SO_4, filtered and concentrated to give the crude material as a yellow oil, and purified by column chromatography (EtOAc:MeOH 0 to 10%) to give the desired product.

2-Electrodes undivided electrochemical cell

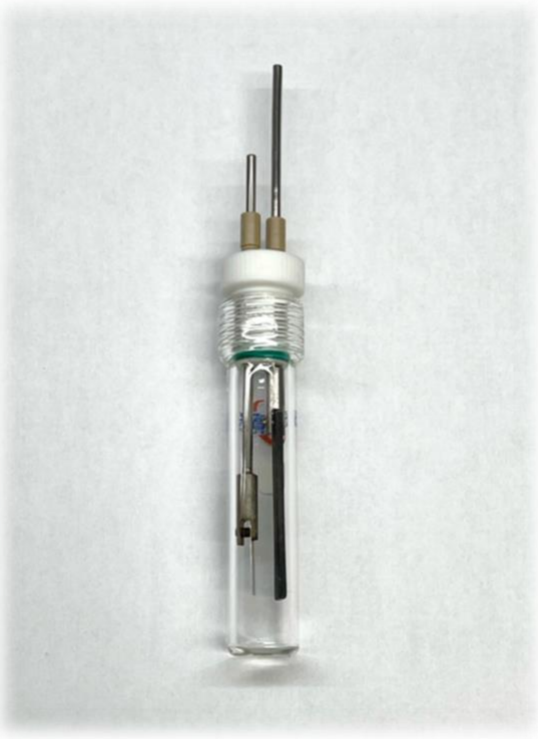

Electrolysis was carried out in an undivided electrochemical cell sealed with a PTFE cap. Graphite and platinum electrodes were connected with stainless steel electrode holders. The cell's diameter was 20 mm, and the distance between the electrodes was 4 mm.

- Graphite anode: Total surface area submerged in solution – 2.16 cm^2.
- Platinum cathode: Total surface area submerged in solution – 1.05 cm^2.

Electrolysis was carried out with TTi MX100QP quad output multi-range DC power supply under constant current conditions.

Unsuccessful Substrates

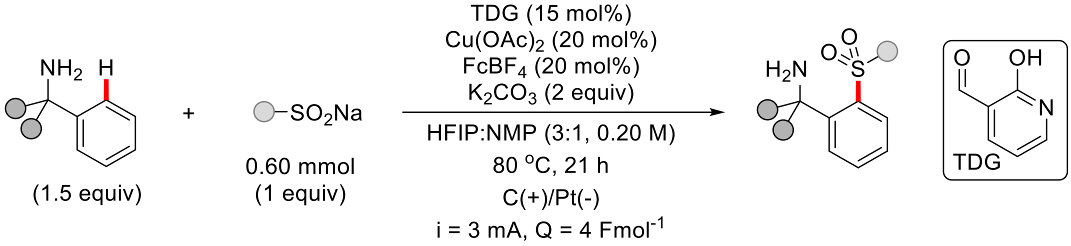

Unsuccessful Benzylamines

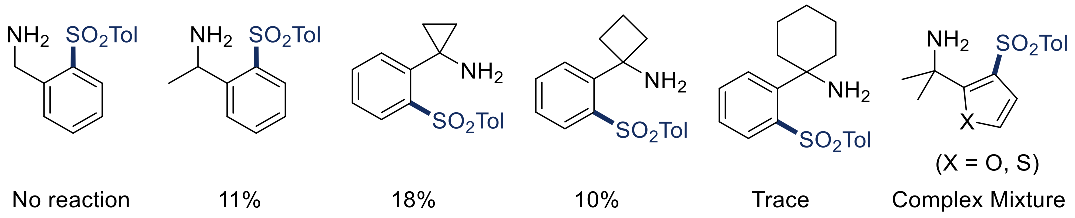

Unsuccessful Sulfinates

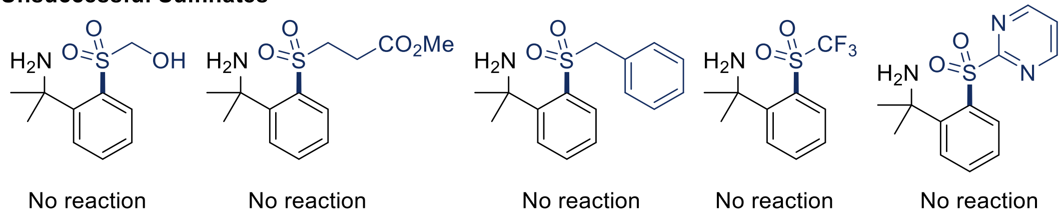

Sulfonylation Reaction Scope: Varying Amines

2-(2-Methoxy-6-tosylphenyl)propan-2-amine 4

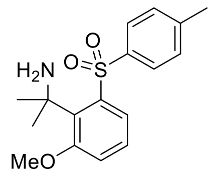

Prepared according to the general procedure using 2-(2-methoxyphenyl)propan-2-amine (149 mg, 0.90 mmol) and sodium *p*-toluenesulfinate (107 mg, 0.60 mmol). Sulfonyl amine **4** was isolated by column chromatography (100% EtOAc to 10% MeOH:EtOAc) as a pale yellow oil (29 mg, 0.09 mmol, 15%). $\mathbf{R_f}$ 0.20 (EtOAc). **^{1}H NMR** (500 MHz, $CDCl_3$) δ 7.71 (d, *J* = 8.2 Hz, 1H, Ar-CH), 7.65 (d, *J* = 8.4 Hz, 2H, 2 × Ar-CH), 7.30 – 7.26 (m, 3H, 3 × Ar-CH), 7.14 (d, *J* = 8.2 Hz, 1H, Ar-CH), 3.88 (s, 3H, OCH_3), 2.43 (s, 3H, Ar-CH_3), 2.10 (s, 2H, NH_2), 1.60 (s, 6H, $C(CH_3)_2$). **^{13}C NMR** (126 MHz, $CDCl_3$) δ 158.5 (Ar-C_q), 143.1 (Ar-C_q), 142.2 (Ar-C_q), 141.7 (Ar-C_q), 141.5 (Ar-C_q), 129.3 (2 × Ar-CH), 126.3 (Ar-CH), 125.4 (2 × Ar-CH), 124.2 (Ar-CH), 116.6 (Ar-CH), 55.9 (OCH_3), 54.9 (C_qNH_2), 30.9 ($C(\mathit{C}H_3)_2$), 21.5 ($ArCH_3$). **IR** (film)/cm^{-1} 2961, 2933, 1431, 1284, 1260, 1146, 732, 669, 565. **HRMS** (TOF—ESI^+) m/z calcd. for $C_{17}H_{22}NO_3S^+$ $[M+H]^+$: 320.1320; found 320.1332.

2-(5-Methoxy-2-tosylphenyl)propan-2-amine 5 & 2-(3-methoxy-2-tosylphenyl)propan-2-amine 5'

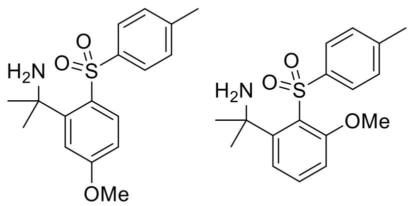

Prepared according to the general procedure using 2-(3-methoxyphenyl)propan-2-amine (149 mg, 0.90 mmol) and sodium *p*-toluenesulfinate (107 mg, 0.60 mmol). Sulfonyl amine **5** (73 mg, 0.23 mmol, 38%) and **5'** (75 mg, 0.24 mmol, 39%) was isolated by column chromatography (100% EtOAc to 10% MeOH:EtOAc) as a yellow oil.

2-(5-Methoxy-2-tosylphenyl)propan-2-amine 5: $\mathbf{R_f}$ 0.34 (EtOAc). **^{1}H NMR** (400 MHz, $CDCl_3$) δ 8.10 (d, *J* = 8.9 Hz, 1H, Ar-CH), 7.65 (d, *J* = 8.4 Hz, 2H, 2 × Ar–CH), 7.26 (d, *J* = 7.5 Hz, 2H, 2 × Ar–CH), 7.13 (d, *J* = 2.7 Hz, 1H, Ar–CH), 6.82 (dd, *J* = 9.0, 2.7 Hz, 1H, Ar–CH), 3.87 (s, 3H, OCH_3), 2.40 (s, 3H, Ar-CH_3), 2.15 (s, 2H, NH_2), 1.62 (s, 6H, $C(\mathit{C}H_3)_2$). **^{13}C NMR** (101 MHz, $CDCl_3$) δ 162.7 (Ar-C_q), 153.9 (Ar-C_q), 143.1 (Ar-C_q), 141.5 (Ar-C_q), 136.09 (Ar-CH), 130.7 (Ar-C_q), 129.5 (2 × Ar-CH), 126.4 (2 × Ar-CH), 115.8 (Ar-CH), 109.7 (Ar-CH), 55.5 (OCH_3), 54.2 ($NH_2\mathit{C}(CH_3)_2$), 33.2 ($NH_2C(\mathit{C}H_3)_2$), 21.5 (Ar-CH_3). The analytical data (^{1}H, ^{13}C NMR) are in agreement with the reported literature.[2]

2-(3-Methoxy-2-tosylphenyl)propan-2-amine 5': $\mathbf{R_f}$ 0.10 (EtOAc). **^{1}H NMR** (500 MHz, $CDCl_3$) δ 7.69 (d, *J* = 8.3 Hz, 2H, 2 × Ar-CH), 7.40 (t, *J* = 8.2 Hz, 1H, Ar-CH), 7.32 (dd, *J* = 8.2, 1.2 Hz, 1H, Ar-CH), 7.26 – 7.22 (m, 2H, 2 × Ar-CH), 6.77 (dd, *J* = 8.2, 1.2 Hz, 1H, Ar-CH), 3.36 (s, 3H, OCH_3), 2.41 (s, 3H, Ar-CH_3), 2.18 (s, 2H, NH_2), 1.80 (s, 6H, $C(CH_3)_2$). **^{13}C NMR** (126 MHz, $CDCl_3$) δ 158.9 (Ar-C_q), 153.6 (Ar-C_q), 143.2 (Ar-C_q), 142.5 (Ar-C_q), 133.6 (Ar-CH), 128.5 (2 × Ar-CH), 126.1 (2 × Ar-CH), 120.7 (Ar-CH), 112.2 (Ar-CH), 56.0 (OCH_3), 54.4 (C_qNH_2), 33.6 ($C(\mathit{C}H_3)_2$), 21.5 (Ar-CH_3). **IR** (film)/cm^{-1} 3394, 2973, 1571, 1467, 1299, 1152, 1087, 1044, 492, 734, 660. **HRMS** (TOF—ESI^+) m/z calcd. for $C_{17}H_{22}NO_3S^+$ $[M+H]^+$: 320.1320; found 320.1316.

2-(4-Methoxy-2-tosylphenyl)propan-2-amine 6

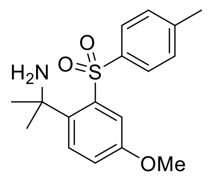

Prepared according to the general procedure using 2-(4-methoxyphenyl)propan-2-amine (149 mg, 0.90 mmol) and sodium *p*-toluenesulfinate (107 mg, 0.60 mmol). Sulfonyl amine **6** was isolated by column chromatography (100% EtOAc to 10% MeOH:EtOAc) as a yellow oil (102 mg, 0.32 mmol, 53%). $\mathbf{R_f}$ 0.10 (EtOAc). **^{1}H NMR** (500 MHz, $CDCl_3$) δ 7.73 (d, *J* = 8.4 Hz, 2H, 2 × Ar-CH), 7.57 (d, *J* = 8.9 Hz, 1H, Ar-CH), 7.48 (d, *J* = 2.9 Hz, 1H, Ar-CH), 7.31 (d, *J* = 8.1 Hz, 2H, 2 × Ar-CH), 7.01 (dd, *J* = 8.9, 2.9 Hz, 1H, Ar-CH), 3.78 (s, 3H, OCH_3), 2.43 (s, 3H, Ar-CH_3), 1.86 (s, 2H, NH_2), 1.68 (s, 6H, $C(CH_3)_2$). **^{13}C NMR** (126 MHz, $CDCl_3$) δ 157.6 (Ar-C_q), 143.8 (2 × Ar-C_q), 140.5 (Ar-C_q), 140.1 (Ar-C_q), 130.1 (Ar-CH), 129.7 (2 × Ar-CH), 127.0 (2 × Ar-CH), 118.6 (Ar-CH), 117.7 (Ar-CH), 55.6 (OCH_3), 54.0 ($NH_2\mathit{C}(CH_3)_2$), 33.2 ($NH_2C(\mathit{C}H_3)_2$),

21.6 (Ar-CH_3). **IR** (film)/cm^{-1} 2977, 2924, 1601, 1299, 1238, 1157, 1139, 587, 550. **HRMS** (TOF—ESI$^+$) m/z calcd. for $C_{17}H_{22}NO_3S^+$ $[M+H]^+$: 320.1320; found 320.1336.

2-(3,4,5-Trimethoxy-2-tosylphenyl)propan-2-amine 7

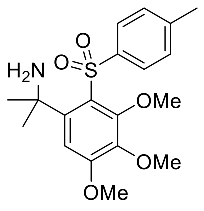

Prepared according to the general procedure using 2-(3,4,5-trimethoxyphenyl)propan-2-amine (202 mg, 0.90 mmol) and sodium *p*-toluenesulfinate (107 mg, 0.60 mmol). Sulfonyl amine **7** was isolated by column chromatography (100% EtOAc to 10% MeOH:EtOAc) as a yellow solid (146 mg, 0.38 mmol, 63%). **Melting Point** 144 – 145 °C (EtOAc/MeOH ppt). **R*f*** 0.10 (EtOAc). **^{1}H NMR** (500 MHz, $CDCl_3$) δ 7.71 (d, *J* = 8.4 Hz, 2H, 2 × Ar-H), 7.25 (d, *J* = 8.1 Hz, 2H, 2 × Ar-H), 7.02 (s, 1H, Ar-H), 3.94 (s, 3H, OCH_3), 3.70 (s, 3H, OCH_3), 3.54 (s, 3H, OCH_3), 3.01 (s, 2H, NH_2), 2.40 (s, 3H, CH_3), 1.85 (s, 6H, $C(CH_3)_2$). **^{13}C NMR** (126 MHz, $CDCl_3$) δ 155.9 (Ar-C_q), 154.4 (Ar-C_q), 146.9 (Ar-C_q), 143.2 (Ar-C_q), 142.6 (Ar-C_q), 141.0 (Ar-C_q), 129.1 (2 × Ar-CH), 126.8 (Ar-C_q), 125.6 (2 × Ar-CH), 106.9 (Ar-CH), 61.0 (OCH_3), 60.6 (OCH_3), 55.9 (OCH_3), 54.6 ($NH_2\mathit{C}(CH_3)_2$), 33.1 ($NH_2C(\mathit{C}H_3)_2$), 21.5 ($ArCH_3$). **IR** (film)/cm^{-1} 2941, 1580, 1562, 1487, 1366, 1310, 1142, 1107, 669, 561. **HRMS** (TOF—ESI$^+$) m/z calcd. for $C_{19}H_{26}NO_5S^+$ $[M+H]^+$: 380.1532; found 380.1522.

1-(2-Tosylphenyl)cyclopentan-1-amine 8

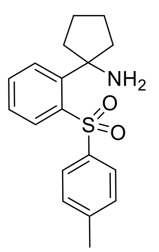

Prepared according to the general procedure using 1-phenylcyclopentan-1-amine (145 mg, 0.90 mmol) and sodium *p*-toluenesulfinate (107 mg, 0.60 mmol). Sulfonyl amine **8** was isolated by column chromatography (100% EtOAc to 10% MeOH:EtOAc) as a white solid (151 mg, 0.48 mmol, 80%). **Melting Point** 86 – 87 °C (EtOAc/MeOH ppt). **R*f*** 0.20 (EtOAc). **^{1}H NMR** (500 MHz, $CDCl_3$) δ 7.82 (dd, *J* = 8.1, 1.5 Hz, 1H, Ar-CH), 7.74 (d, *J* = 8.4 Hz, 2H, 2 × Ar-CH), 7.67 (dd, *J* = 8.0, 1.3 Hz, 1H, Ar-CH), 7.50 (td, *J* = 7.7, 1.5 Hz, 1H, Ar-CH), 7.32 (d, *J* = 8.2 Hz, 2H, 2 × Ar-CH), 7.30 – 7.28 (m, 1H, Ar-CH), 2.44 (s, 3H, Ar-CH_3), 2.42 – 2.35 (m, 2H, 2 × $C(C\mathit{H}H)_2(CH_2)_2$), 1.97 (s, 2H, NH_2), 1.94 – 1.84 (m, 4H, 2 × $C(CH\mathit{H})_2(C\mathit{H}H)_2$), 1.75 – 1.66 (m, 2H, 2 × $C(CH_2)_2(CH\mathit{H})_2$). **^{13}C NMR** (126 MHz, $CDCl_3$) δ 149.9 (Ar-C_q), 143.9 (Ar-C_q), 139.61 (Ar-C_q), 139.58 (Ar-C_q), 133.1 (Ar-CH), 132.3 (Ar-CH), 129.7 (2 × Ar-CH), 128.7 (Ar-CH), 127.4 (2 × Ar-CH), 126.6 (Ar-CH), 64.7 (C_qNH_2), 40.7 ($C(\mathit{C}H_2)_2(CH_2)_2$), 23.0 ($C(CH_2)_2(\mathit{C}H_2)_2$), 21.6 (Ar-$CH_3$). **IR** (film)/$cm^{-1}$ 3383, 2947, 2866, 1299, 1148, 1090, 654, 580. **HRMS** (TOF—ESI$^+$) m/z calcd. for $C_{18}H_{22}NO_2S^+$ $[M+H]^+$: 316.1371; found 316.1381. SCXRD: A portion of the compound was crystallised in EtOH to give clear colourless blocks. Crystal data for $C_{18}H_{21}NO_2S$ (m = 315.42 g/mol): monoclinic, space group $P2_1/c$ (no. 14).

2-(4-Fluoro-2-tosylphenyl)propan-2-amine 9

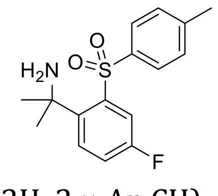

Prepared according to the general procedure using 2-(4-fluorophenyl)propan-2-amine (138 mg, 0.90 mmol) and sodium *p*-toluenesulfinate (107 mg, 0.60 mmol). Sulfonyl amine **9** was isolated by column chromatography (100% EtOAc to 10% MeOH:EtOAc) as a white solid (83 mg, 0.27 mmol, 45%). **Melting Point** 120 – 122 °C (EtOAc/MeOH ppt). **R*f*** 0.23 (EtOAc). **^{1}H NMR** (500 MHz, $CDCl_3$) δ 7.72 (d, *J* = 8.3 Hz, 2H, 2 × Ar-CH), 7.66 (dd, *J* = 9.4, 2.9 Hz, 1H, Ar-CH), 7.62 (dd, *J* = 8.9, 5.5 Hz, 1H, Ar-CH), 7.31 (d, *J* = 8.1 Hz, 2H, 2 × Ar-CH), 7.18 (ddd, *J* = 8.8, 6.9, 2.9 Hz, 1H, Ar-CH), 2.43 (s, 3H, Ar-CH_3), 1.91 (s, 2H, NH_2), 1.67 (s, 6H, $C(CH_3)_2$). **^{13}C NMR** (126 MHz, $CDCl_3$) δ 160.2 (d, *J* = 250.1 Hz, Ar-C_q), 144.0 (2 × Ar-C_q), 141.8 (d, *J* = 4.7 Hz, Ar-C_q), 139.7 (Ar-C_q), 130.7 (d, *J* = 7.1 Hz, Ar-CH), 129.8 (2 × Ar-CH), 127.1 (2 × Ar-CH), 119.8 (d, *J* = 25.1 Hz, Ar-CH), 119.4 (d, *J* = 19.6 Hz, Ar-CH), 54.1 (C_qNH_2), 33.5 ($C(CH_3)_2$), 21.6 ($ArCH_3$). **^{19}F NMR** (377 MHz, $CDCl_3$) δ −114.0. **IR** (film)/cm^{-1} 2924, 2971, 1597, 1478, 1301, 1221, 1150, 693, 652, 585, 550. **HRMS** (TOF—ESI$^+$) m/z calcd. for $C_{16}H_{19}NO_2SF^+$ $[M+H]^+$: 308.1121; found 308.1122. SCXRD: A portion of the compound was crystallised in EtOH to give clear colourless plates. Crystal data for $C_{16}H_{18}FNO_2S$ (m = 307.37 g/mol): orthorhombic, space group $Pna2_1$ (no. 33).

2-(4-Chloro-2-tosylphenyl)propan-2-amine 10

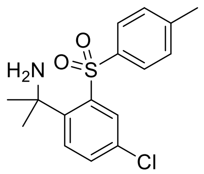

Prepared according to the general procedure using 2-(4-chlorophenyl)propan-2-amine (153 mg, 0.90 mmol) and sodium *p*-toluenesulfinate (107 mg, 0.60 mmol). Sulfonyl amine **10** was isolated by column chromatography (100% EtOAc to 10% MeOH:EtOAc) as a yellow oil (82 mg, 0.25 mmol, 42%). **R*f*** 0.23 (EtOAc). **^{1}H NMR** (500 MHz, $CDCl_3$) δ 8.00 (d, *J* = 2.4 Hz, 1H, Ar-CH), 7.72 (d, *J* = 8.4 Hz, 2H, 2 × Ar-CH), 7.57 (d, *J* = 8.6 Hz, 1H, Ar-CH), 7.46 (dd, *J* = 8.6, 2.4 Hz, 1H, Ar-CH), 7.32 (d, *J* = 8.3 Hz, 2H, 2 × Ar-CH), 2.44 (s, 3H, Ar-CH_3), 1.90 (s, 2H, NH_2), 1.65 (s, 6H, C(CH_3)$_2$). **^{13}C NMR** (126 MHz, $CDCl_3$) δ 149.7 (Ar-C_q), 143.9 (Ar-C_q), 141.4 (Ar-C_q), 140.0 (Ar-C_q), 132.7 (Ar-CH), 132.6 (Ar-C_q), 132.5 (Ar-CH), 130.2 (Ar-CH), 129.7 (2 × Ar-CH), 126.9 (2 × Ar-CH), 54.2 (C_qNH_2), 33.4 (C(CH_3)$_2$), 21.6 (Ar-CH_3). **IR** (film)/cm^{-1} 2969, 2924, 1299, 1141, 1090, 813, 684, 580, 550. **HRMS** (TOF—ESI$^+$) m/z calcd. for $C_{16}H_{19}NO_2SCl^+$ [M+H]$^+$: 324.0825; found 324.0820. SCXRD: A portion of the compound was crystallised in EtOH to give clear colourless blocks. Crystal data for $C_{16}H_{18}ClNO_2S$ (m = 323.82 g/mol): orthorhombic, space group *P*ca2$_1$ (no. 29).

2-(4-Bromo-2-tosylphenyl)propan-2-amine 11

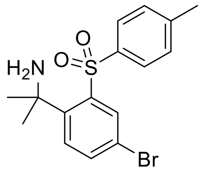

Prepared according to the general procedure using 2-(4-bromophenyl)propan-2-amine (193 mg, 0.90 mmol) and sodium *p*-toluenesulfinate (107 mg, 0.60 mmol). Sulfonyl amine **11** was isolated by column chromatography (100% EtOAc to 10% MeOH:EtOAc) as a white solid (69 mg, 0.19 mmol, 31%). **Melting Point** 124 – 126 °C (EtOAc/MeOH ppt). **R*f*** 0.23 (EtOAc). **^{1}H NMR** (500 MHz, $CDCl_3$) δ 8.16 (d, *J* = 2.2 Hz, 1H, Ar-CH), 7.71 (d, *J* = 8.3 Hz, 2H, 2 × Ar-CH), 7.61 (dd, *J* = 8.6, 2.2 Hz, 1H, Ar-CH), 7.50 (d, *J* = 8.6 Hz, 1H, Ar-CH), 7.32 (d, *J* = 8.3 Hz, 2H, 2 × Ar-CH), 2.44 (s, 3H, Ar-CH_3), 1.81 (s, 2H, NH_2), 1.65 (s, 6H, C(CH_3)$_2$). **^{13}C NMR** (126 MHz, $CDCl_3$) δ 150.1 (Ar-C_q), 143.9 (Ar-C_q), 141.6 (Ar-C_q), 140.1 (Ar-C_q), 135.8 (Ar-CH), 135.3 (Ar-CH), 130.5 (Ar-CH), 129.7 (2 × Ar-CH), 126.9 (2 × Ar-CH), 120.4 (Ar-C_q), 54.3 (C_qNH_2), 33.4 (C(CH_3)$_2$), 21.6 (Ar-CH_3). **IR** (film)/cm^{-1} 3398, 2965, 2926, 1299, 1142, 1090, 1038, 811, 682, 650, 580. **HRMS** (TOF—ESI$^+$) m/z calcd. for $C_{16}H_{19}NO_2SBr^+$ [M+H]$^+$: 368.0320; found 368.0312. SCXRD: A portion of the compound was crystallised in EtOH to give clear colourless blocks. Crystal data for $C_{16}H_{18}ClNO_2S$ (m = 323.82 g/mol): orthorhombic, space group *P*ca2$_1$ (no. 29).

2-(2-Tosyl-4-(trifluoromethyl)phenyl)propan-2-amine 12

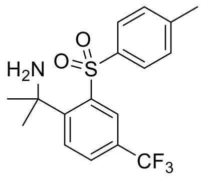

Prepared according to the general procedure using 2-(4-(trifluoromethyl)phenyl)propan-2-amine (183 mg, 0.90 mmol) and sodium *p*-toluenesulfinate (107 mg, 0.60 mmol). Sulfonyl amine **12** was isolated by column chromatography (100% EtOAc to 10% MeOH:EtOAc) as a yellow oil (149 mg, 0.42 mmol, 70%). **R*f*** 0.43 (EtOAc). **^{1}H NMR** (500 MHz, $CDCl_3$) δ 8.32 (s, 1H, Ar-CH), 7.78 – 7.72 (m, 2H, 2 × Ar-CH), 7.70 (d, *J* = 8.4 Hz, 2H, 2 × Ar-CH), 7.32 (d, *J* = 8.1 Hz, 2H, 2 × Ar-CH), 2.44 (s, 3H, Ar-CH_3), 1.92 (s, 2H, NH_2), 1.67 (s, 6H, C(CH_3)$_2$). **^{13}C NMR** (126 MHz, $CDCl_3$) δ 155.7 (Ar-C_q), 143.9 (Ar-C_q), 141.0 (Ar-C_q), 140.0 (Ar-C_q), 129.7 (2 × Ar-CH), 129.5 (Ar-CH), 129.4 (q, *J* = 3.2 Hz, 2 × Ar-CH), 129.1 (q, *J* = 33.8 Hz, Ar-CH), 126.9 (2 × Ar-CH), 123.3 (q, *J* = 278 Hz, CF_3), 54.5 (NH_2*C*(CH_3)$_2$), 33.5 (NH_2C(*C*H_3)$_2$), 21.6 (Ar-CH_3). **^{19}F NMR** (471 MHz, $CDCl_3$) δ –62.8. **IR** (film)/cm^{-1} 2932, 1614, 1328, 1300, 1131, 1187, 572. **HRMS** (TOF—ESI$^+$) m/z calcd. for $C_{17}H_{19}NO_2F_3S^+$ [M+H]$^+$: 358.1089; found 358.1089.

2-(3-Tosyl-[1,1'-biphenyl]-4-yl)propan-2-amine 13

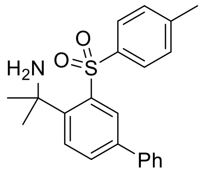

Prepared according to the general procedure using 2-([1,1'-biphenyl]-4-yl)propan-2-amine (190 mg, 0.90 mmol) and sodium *p*-toluenesulfinate (107 mg, 0.60 mmol). Sulfonyl amine **13** was isolated by column chromatography (100% EtOAc to 10% MeOH:EtOAc) as a colourless oil (79 mg, 0.22 mmol, 36%). $\mathbf{R_f}$ 0.20 (EtOAc). **^{1}H NMR** (400 MHz, $CDCl_3$) δ 8.29 (d, *J* = 1.8 Hz, 1H, Ar-CH), 7.80 – 7.69 (m, 4H, 4 × Ar-CH), 7.57 – 7.49 (m, 2H, 2 × Ar-CH), 7.45 (t, *J* = 7.4 Hz, 2H, 2 × Ar-CH), 7.40 (d, *J* = 7.2 Hz, 1H, Ar-CH), 7.30 (d, *J* = 8.1 Hz, 2H, 2 × Ar-CH), 2.42 (s, 3H, Ar-CH_3), 2.23 (s, 2H, NH_2), 1.69 (s, 6H, $C(CH_3)_2$). **^{13}C NMR** (101 MHz, $CDCl_3$) δ 150.4 (Ar-C_q), 143.5 (Ar-C_q), 140.7 (Ar-C_q), 139.9 (Ar-C_q), 139.4 (Ar-C_q), 138.7 (Ar-C_q), 131.4 (Ar-CH), 131.0 (Ar-CH), 129.6 (2 × Ar-CH), 129.3 (Ar-CH), 129.0 (2 × Ar-CH), 128.1 (Ar-CH), 126.9 (2 × Ar-CH), 126.8 (2 × Ar-CH), 54.1 ($NH_2\mathit{C}(CH_3)_2$), 33.6 ($NH_2C(\mathit{C}H_3)_2$), 21.5 (Ar-CH_3). **IR** (film)/cm^{-1} 2396, 2966, 2924, 1596, 1470, 1293, 1141, 1087, 763, 696, 579. **HRMS** (TOF—ESI$^+$) m/z calcd. for $C_{22}H_{24}NO_2S^+$ $[M+H]^+$: 366.1528; found 366.1516.

Sulfonylation Reaction Scope: Varying Sulfinate Salts

2-(2-Tosylphenyl)propan-2-amine 3

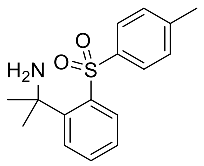

Prepared according to the general procedure using 1-methyl-1-phenylethylamine (122 mg, 0.90 mmol) and sodium *p*-toluenesulfinate (107 mg, 0.60 mmol). Sulfonyl amine **3** was isolated by column chromatography (100% EtOAc to 10% MeOH:EtOAc) as a yellow oil (129 mg, 0.45 mmol, 74%). **R*f*** 0.15 (EtOAc). **^{1}H NMR** (400 MHz, $CDCl_3$) δ 7.94 (dd, *J* = 8.1, 1.4 Hz, 1H, Ar-CH), 7.72 (d, *J* = 8.3 Hz, 2H, 2 × Ar-CH), 7.64 (dd, *J* = 8.1, 1.3 Hz, 1H, Ar-CH), 7.54 – 7.46 (m, 1H, Ar-CH), 7.39 – 7.27 (m, 3H, 3 × Ar-CH), 2.43 (s, 3H, Ar-CH_3), 2.22 (brs, 2H, NH_2), 1.67 (s, 6H, $C(CH_3)_2$). **^{13}C NMR** (101 MHz, $CDCl_3$) δ 151.6 (Ar-C_q), 143.5 (Ar-C_q), 140.6 (Ar-C_q), 139.6 (Ar-C_q), 132.9 (Ar-CH), 132.8 (Ar-CH), 129.6 (2 × Ar-CH), 128.7 (Ar-CH), 126.9 (2 × Ar-CH), 126.6 (Ar-CH), 54.2 (C_qNH_2), 33.5 ($C(CH_3)_2$), 21.5 (Ar-CH_3). The analytical data (^{1}H, ^{13}C NMR) are in agreement with the reported literature.[2]

2-(2-(m-Tolylsulfonyl)phenyl)propan-2-amine 14

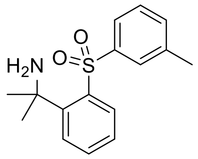

Prepared according to the general procedure using 1-methyl-1-phenylethylamine (122 mg, 0.90 mmol) and sodium 3-methylbenzenesulfinate (107 mg, 0.60 mmol). Sulfonyl amine **14** was isolated by column chromatography (100% EtOAc to 10% MeOH:EtOAc) as a yellow oil (95 mg, 0.33 mmol, 55%). **R*f*** 0.27 (EtOAc). **^{1}H NMR** (400 MHz, $CDCl_3$) δ 7.99 (dd, *J* = 8.1, 1.5 Hz, 1H, Ar-CH), 7.68 – 7.62 (m, 2H, 2 × Ar-CH), 7.62 – 7.56 (m, 1H, Ar-CH), 7.56 – 7.47 (m, 1H, Ar-CH), 7.42 – 7.30 (m, 3H, 3 × Ar-CH), 2.41 (s, 3H, Ar-CH_3), 2.10 (s, 2H, NH_2), 1.67 (s, 6H, $C(CH_3)_2$). **^{13}C NMR** (101 MHz, $CDCl_3$) δ 151.7 (Ar-C_q), 143.5 (Ar-C_q), 139.3 (Ar-C_q), 133.4 (Ar-CH), 133.0 (Ar-CH), 132.9 (Ar-CH), 128.8 (Ar-CH), 128.7 (Ar-CH), 128.2 (Ar-C_q), 126.9 (Ar-CH), 126.6 (Ar-CH), 123.9 (Ar-CH), 54.2 (C_qNH_2), 33.5 ($C(CH_3)_2$), 21.4 (Ar-CH_3). **IR** (film)/cm^{-1} 3392, 2965, 2924, 1293, 1146, 764, 742, 688. **HRMS** (TOF—ESI$^+$) m/z calcd. for $C_{16}H_{19}NO_2S^+$ [M+H]$^+$: 290.1215; found 290.1216.

2-(2-(o-Tolylsulfonyl)phenyl)propan-2-amine 15

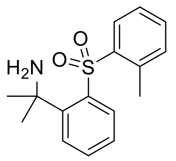

Prepared according to the general procedure using 1-methyl-1-phenylethylamine (122 mg, 0.90 mmol) and sodium 2-methylbenzenesulfinate (107 mg, 0.60 mmol). Sulfonyl amine **15** was isolated by column chromatography (100% EtOAc to 10% MeOH:EtOAc) as a yellow solid (70 mg, 0.240 mmol, 40%). **R*f*** 0.20 (EtOAc). **Melting Point** 96 – 98 °C (EtOAc/MeOH ppt). **^{1}H NMR** (400 MHz, $CDCl_3$) δ 8.00 (dd, *J* = 8.0, 1.4 Hz, 1H, Ar-CH), 7.68 (dd, *J* = 8.1, 1.3 Hz, 1H, Ar-CH), 7.56 – 7.44 (m, 3H, 3 × Ar-CH), 7.39 (t, *J* = 7.7 Hz, 1H, Ar-CH), 7.30 (d, *J* = 7.6 Hz, 1H, Ar-CH), 7.24 – 7.16 (m, 1H, Ar-CH), 2.37 (s, 3H, Ar-CH_3), 2.24 (s, 2H, NH_2), 1.72 (s, 6H, $C(CH_3)_2$). **^{13}C NMR** (101 MHz, $CDCl_3$) δ 151.5 (Ar-C_q), 140.9 (Ar-C_q), 140.0 (Ar-C_q), 137.4 (Ar-C_q), 133.0 (Ar-CH), 132.7 (Ar-CH), 132.5 (Ar-CH), 130.4 (Ar-CH), 128.8 (Ar-CH), 128.4 (Ar-CH), 126.5 (Ar-CH), 126.1 (Ar-CH), 54.2 (C_qNH_2), 33.2 ($C(CH_3)_2$), 20.3 (Ar-CH_3). **IR** (film)/cm^{-1} 3390, 2973, 2932, 1459, 1290, 1152, 1124, 805, 760, 742. **HRMS** (TOF—ESI$^+$) m/z calcd. for $C_{16}H_{20}NO_2S^+$ [M+H]$^+$: 290.1215; found 290.1213.

2-(2-(Cyclopropylsulfonyl)phenyl)propan-2-amine 17

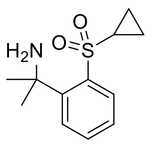

Prepared according to the general procedure using 1-methyl-1-phenylethylamine (122 mg, 0.90 mmol) and cyclopropane sulfinic acid sodium salt (77 mg, 0.60 mmol). Sulfonyl amine **17** was isolated by column chromatography (100% EtOAc to 10% MeOH:EtOAc) as a yellow solid (81 mg, 0.34 mmol, 56%). **R*f*** 0.10 (EtOAc). **Melting Point** 105 – 107 °C (EtOAc/MeOH ppt). **^{1}H NMR** (400 MHz, $CDCl_3$) δ 8.06 (d, *J* = 8.1 Hz, 1H, Ar-CH), 7.59 (d, *J* = 7.2 Hz, 1H, Ar-CH), 7.55 – 7.46 (m, 1H, Ar-CH), 7.40 – 7.32 (m, 1H, Ar-CH), 3.77 (tt, *J* = 8.1, 4.9 Hz, 1H, SO_2CH), 2.40 (s, 2H, NH_2), 1.71 (s, 6H, $C(CH_3)_2$), 1.40 – 1.31 (m, 2H, C*H*HC*H*H), 1.03 – 0.95 (m, 2H, CH*H*CH*H*). **^{13}C NMR** (101 MHz, $CDCl_3$) δ 151.0 (Ar-C_q), 139.6 (Ar-C_q), 132.8 (Ar-CH), 132.1 (Ar-CH), 128.4 (Ar-CH), 126.6

(Ar-CH), 54.2 ($NH_2\mathit{C}(CH_3)_2$), 34.8 ($NH_2C(\mathit{C}H_3)_2$), 34.3 (SO_2CH), 6.3 ($\mathit{C}H_2\mathit{C}H_2$). The analytical data (^{1}H, ^{13}C NMR) are in agreement with the reported literature.[2]

2-(2-(Bicyclo[1.1.1]pentan-1-ylsulfonyl)phenyl)propan-2-amine 18

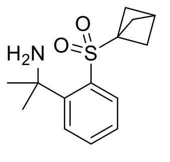

Prepared according to the general procedure using 1-methyl-1-phenylethylamine (122 mg, 0.90 mmol) and bicyclo[1.1.1]pentan-1-ylsulfinic acid sodium salt (92 mg, 0.60 mmol). Sulfonyl amine **18** was isolated by column chromatography (100% EtOAc to 10% MeOH:EtOAc) as a yellow solid (61 mg, 0.23 mmol, 38%). **R_f** 0.11 (EtOAc). **Melting Point** 70 – 73 °C (EtOAc/MeOH ppt). **^{1}H NMR** (400 MHz, $CDCl_3$) δ 8.03 (dd, *J* = 8.0, 1.6 Hz, 1H, Ar-CH), 7.66 (d, *J* = 8.1 Hz, 1H, Ar-CH), 7.53 (t, *J* = 7.3 Hz, 1H, Ar-CH), 7.40 (t, *J* = 7.6 Hz, 1H, Ar-CH), 2.73 (s, 1H, $C(CH_2)_3\mathit{C}H$), 2.40 (s, 2H, NH_2), 2.15 (s, 6H, $C(C\mathit{H}_2)_3CH$), 1.68 (s, 6H, $C(CH_3)_2$). **^{13}C NMR** (101 MHz, $CDCl_3$) δ 152.3 (Ar-C_q), 136.5 (Ar-C_q), 134.2 (Ar-CH), 133.2 (Ar-CH), 127.7 (Ar-CH), 126.6 (Ar-CH), 57.1 (SO_2C_q), 53.4 ($NH_2C(CH_3)_2$), 51.3 ($SO_2C(\mathit{C}H_2)_3CH$), 33.5 ($NH_2C(\mathit{C}H_3)_2$, 26.1 ($SO_2C(CH_2)_3\mathit{C}H$). The analytical data (^{1}H, ^{13}C NMR) are in agreement with the reported literature.[2]

2-(2-(4-Fluorobenzenesulfonyl)phenyl)propan-2-amine 19

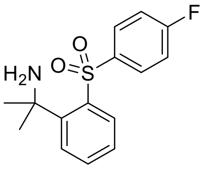

Prepared according to the general procedure using 1-methyl-1-phenylethylamine (122 mg, 0.90 mmol) and sodium 4-fluorobenzenesulfinate (109 mg, 0.60 mmol). Sulfonyl amine **19** was isolated by column chromatography (100% EtOAc to 10% MeOH:EtOAc) as a yellow oil (94 mg, 0.32 mmol, 53%). **R_f** 0.23 (EtOAc). **^{1}H NMR** (400 MHz, $CDCl_3$) δ 8.03 (d, *J* = 8.1 Hz, 1H, Ar-CH), 7.84 (dd, *J* = 8.7, 5.1 Hz, 2H, 2 × Ar-CH), 7.63 (d, *J* = 8.0 Hz, 1H, Ar-CH), 7.52 (t, *J* = 7.7 Hz, 1H, Ar-CH), 7.35 (t, *J* = 7.7 Hz, 1H, Ar-CH), 7.16 (t, *J* = 8.6 Hz, 2H, 2 × Ar-CH), 2.08 (brs, 2H, NH_2), 1.66 (s, 6H, $C(CH_3)_2$). **^{13}C NMR** (101 MHz, $CDCl_3$) δ 164.8 (d, *J* = 254.6 Hz, Ar-C_q), 151.8 (Ar-C_q), 140.1 (d, *J* = 3.5 Hz, Ar-C_q), 139.2 (Ar-C_q), 133.2 (Ar-CH), 132.9 (Ar-CH), 129.5 (d, *J* = 9.2 Hz, 2 × Ar-CH), 128.7 (Ar-CH), 126.7 (Ar-CH), 116.1 (d, *J* = 22.7 Hz, 2 × Ar-CH), 54.2 ($NH_2\mathit{C}(CH_3)_2$), 33.8($NH_2C(\mathit{C}H_3)_2$). **^{19}F NMR** (377 MHz, $CDCl_3$) δ −105.4. The analytical data (^{1}H, ^{13}C, ^{19}F NMR) are in agreement with the reported literature.[2]

2-(2-(4-Chlorobenzenesulfonyl)phenyl)propan-2-amine 20

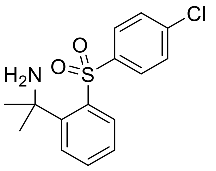

Prepared according to the general procedure using 1-methyl-1-phenylethylamine (122 mg, 0.90 mmol) and sodium 4-chlorobenzenesulfinate (119 mg, 0.60 mmol). Sulfonyl amine **20** was isolated by column chromatography (100% EtOAc to 10% MeOH:EtOAc) as a yellow oil (118 mg, 0.38 mmol, 64%). **R_f** 0.15 (EtOAc). **^{1}H NMR** (400 MHz, $CDCl_3$) δ 8.10 (dd, *J* = 8.1, 1.5 Hz, 1H, Ar-CH), 7.78 – 7.70 (m, 2H, 2 × Ar-CH), 7.61 (dd, *J* = 8.0, 1.4 Hz, 1H, Ar-CH), 7.57 – 7.49 (m, 1H, Ar-CH), 7.47 – 7.42 (m, 2H, 2 × Ar-CH), 7.37 (ddd, *J* = 8.4, 7.1, 1.4 Hz, 1H, Ar-CH), 2.05 (s, 2H, NH_2), 1.64 (s, 6H, $C(CH_3)_2$). **^{13}C NMR** (101 MHz, $CDCl_3$) δ 151.9 (Ar-C_q), 143.0 (Ar-C_q), 138.8 (Ar-C_q), 138.6 (Ar-C_q), 133.3 (Ar-CH), 133.0 (Ar-CH), 129.1 (2 × Ar-CH), 128.7 (Ar-CH), 127.9 (2 × Ar-CH), 126.7 (Ar-CH), 54.1 ($NH_2\mathit{C}(CH_3)_2$), 33.8 ($NH_2C(\mathit{C}H_3)_2$). The analytical data (^{1}H, ^{13}C NMR) are in agreement with the reported literature.[2]

2-(2-(4-Bromobenzenesulfonyl)phenyl)propan-2-amine 21

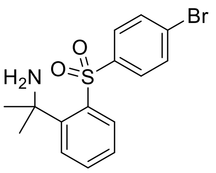

Prepared according to the general procedure using 1-methyl-1-phenylethylamine (122 mg, 0.90 mmol) and sodium 4-bromobenzenesulfinate (146 mg, 0.60 mmol). Sulfonyl amine **21** was isolated by column chromatography (100% EtOAc to 10% MeOH:EtOAc) as a yellow solid (98 mg, 0.28 mmol, 46%). **R_f** 0.17 (EtOAc). **Melting Point** 101 – 103 °C (EtOAc/MeOH ppt). **^{1}H NMR** (400 MHz, $CDCl_3$) δ 8.11 (d, *J* = 8.1 Hz, 1H, Ar-CH), 7.67 (d, *J* = 8.6 Hz, 2H, 2 × Ar-CH), 7.64 – 7.58 (m, 3H, 3 × Ar-CH), 7.57 – 7.51 (m, 1H, Ar-CH), 7.41 – 7.33 (m, 1H, Ar-CH), 2.00 (brs, 2H, NH_2), 1.64 (s, 6H, $C(CH_3)_2$). **^{13}C NMR** (101 MHz, $CDCl_3$) δ 152.0 (Ar-C_q), 143.6 (Ar-C_q), 138.7 (Ar-C_q), 133.3 (Ar-CH), 133.1 (Ar-CH), 132.0 (2 × Ar-CH), 128.7 (Ar-CH), 128.0 (2 × Ar-CH), 127.1 (Ar-C_q), 126.7 (Ar-CH), 54.1 ($NH_2\mathit{C}(CH_3)_2$), 33.9 ($NH_2C(\mathit{C}H_3)_2$). The analytical data (^{1}H, ^{13}C NMR) are in agreement with the reported literature.[2]

2-(2-(Naphthalen-2-ylsulfonyl)phenyl)propan-2-amine 22

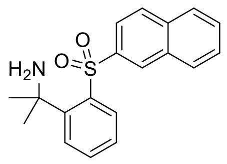

Prepared according to the general procedure using 1-methyl-1-phenylethylamine (122 mg, 0.90 mmol) and sodium naphthalene-2-sulfinate (129 mg, 0.60 mmol). Sulfonyl amine **22** was isolated by column chromatography (100% EtOAc to 10% MeOH:EtOAc) as a yellow oil (59 mg, 0.18 mmol, 30%). **R*f*** 0.31 (EtOAc). **^{1}H NMR** (500 MHz, $CDCl_3$) δ 8.45 – 8.42 (m, 1H, Ar-CH), 8.04 (dd, *J* = 8.1, 1.4 Hz, 1H, Ar-CH), 7.98 – 7.88 (m, 3H, 3 × Ar-CH), 7.77 (dd, *J* = 8.7, 1.9 Hz, 1H, Ar-CH), 7.68 – 7.57 (m, 3H, 3 × Ar-CH), 7.55 – 7.49 (m, 1H, Ar-CH), 7.33 (ddd, *J* = 8.1, 7.3, 1.4 Hz, 1H, Ar-CH), 2.08 (s, 2H, NH_2), 1.69 (s, 6H, $C(CH_3)_2$). **^{13}C NMR** (126 MHz, $CDCl_3$) δ 151.8 (Ar-C_q), 140.7 (Ar-C_q), 139.4 (Ar-C_q), 134.7 (Ar-C_q), 133.0 (Ar-CH), 132.9 (Ar-CH), 132.1 (Ar-C_q), 129.3 (Ar-CH), 129.2 (Ar-CH), 128.9 (Ar-CH), 128.7 (Ar-CH), 127.9 (Ar-CH), 127.7 (Ar-CH), 127.6 (Ar-CH), 126.6 (Ar-CH), 122.3 (Ar-CH), 54.2 (C_qNH_2), 33.6 ($C(CH_3)_2$). **IR** (film)/cm^{-1} 3983, 3056, 2967, 1590, 1295, 1148, 1128, 1074, 855, 814, 747, 673. **HRMS** (TOF—ESI^+) m/z calcd. for $C_{19}H_{20}NO_2S^+$ $[M+H]^+$: 326.1215; found 326.1213.

2-(2-(Phenylsulfonyl)phenyl)propan-2-amine 23

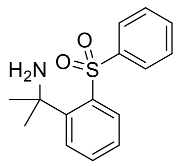

Prepared according to the general procedure using 1-methyl-1-phenylethylamine (122 mg, 0.90 mmol) and sodium benzenesulfinate (98 mg, 0.60 mmol). Sulfonyl amine **23** was isolated by column chromatography (100% EtOAc to 10% MeOH:EtOAc) as a yellow oil (88 mg, 0.32 mmol, 53%). **R*f*** 0.15 (EtOAc). **R*f*** 0.15 (EtOAc). **^{1}H NMR** (400 MHz, $CDCl_3$) δ 8.05 (dd, *J* = 8.1, 1.5 Hz, 1H, Ar-CH), 7.87 – 7.77 (m, 2H, 2 × Ar-CH), 7.64 (dd, *J* = 8.1, 1.4 Hz, 1H, Ar-CH), 7.60 – 7.46 (m, 4H, 4 × Ar-CH), 7.35 (ddd, *J* = 8.3, 7.2, 1.4 Hz, 1H, Ar-CH), 2.10 (s, 2H, NH_2), 1.66 (s, 6H, $C(CH_3)_2$). **^{13}C NMR** (101 MHz, $CDCl_3$) δ 151.9 (Ar-C_q), 144.0 (Ar-C_q), 139.2 (Ar-C_q), 133.1 (2 × Ar-CH), 132.5 (Ar-CH), 129.0 (2 × Ar-CH), 128.7 (Ar-CH), 126.63 (Ar-CH), 126.60 (2 × Ar-CH), 54.2 (C_qNH_2), 33.7 ($C(CH_3)_2$). The analytical data (^{1}H, ^{13}C NMR) are in agreement with the reported literature.[2]

2-(2-(4-*tert*-Butylbenzenesulfonyl)phenyl)propan-2-amine 24

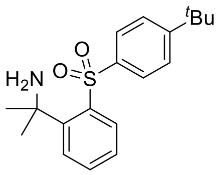

Prepared according to the general procedure using 1-methyl-1-phenylethylamine (122 mg, 0.90 mmol) and 4-*tert*-butylbenzene sulfinic acid sodium salt (132 mg, 0.60 mmol). Sulfonyl amine **24** was isolated by column chromatography (100% EtOAc to 10% MeOH:EtOAc) as a yellow oil (105 mg, 0.32 mmol, 53%). **R*f*** 0.31 (EtOAc). **^{1}H NMR** (500 MHz, $CDCl_3$) δ 7.99 (dd, *J* = 8.1, 1.5 Hz, 1H, Ar-CH), 7.75 (d, *J* = 8.7 Hz, 2H, 2 × Ar-CH), 7.64 (dd, *J* = 8.0, 1.3 Hz, 1H, Ar-CH), 7.54 – 7.48 (m, 3H, 3 × Ar-CH), 7.33 (ddd, *J* = 8.3, 7.3, 1.3 Hz, 1H, Ar-CH), 2.10 (s, 2H, NH_2), 1.69 (s, 6H, $C(CH_3)_2$), 1.33 (s, 9H, $C(CH_3)_3$). **^{13}C NMR** (126 MHz, $CDCl_3$) δ 156.5 (Ar-C_q), 151.4 (Ar-C_q), 140.4 (Ar-C_q), 139.6 (Ar-C_q), 132.9 (2 × Ar-CH), 128.6 (Ar-CH), 126.73 (2 × Ar-CH), 126.66 (Ar-CH), 126.0 (2 × Ar-CH), 54.3 ($H_2NC(CH_3)_2$), 35.2 ($C(CH_3)_3$), 33.4 ($H_2NC(CH_3)_2$), 31.1 ($C(CH_3)_3$). The analytical data (^{1}H, ^{13}C NMR) are in agreement with the reported literature.[2]

2-(2-((4-Methoxyphenyl)sulfonyl)phenyl)propan-2-amine 25

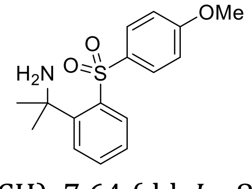

Prepared according to the general procedure using 1-methyl-1-phenylethylamine (122 mg, 0.90 mmol) and sodium 4-methoxybenzenesulfinate (117 mg, 0.60 mmol). Sulfonyl amine **25** was isolated by column chromatography (100% EtOAc to 10% MeOH:EtOAc) as a yellow oil (123 mg, 0.40 mmol, 67%). **R*f*** 0.10 (EtOAc). **^{1}H NMR** (400 MHz, $CDCl_3$) δ 7.87 (dd, *J* = 8.1, 1.5 Hz, 1H, Ar-CH), 7.85 – 7.75 (m, 2H, 2 × Ar-CH), 7.64 (dd, *J* = 8.1, 1.3 Hz, 1H, Ar-CH), 7.51 – 7.42 (m, 1H, Ar-CH), 7.34 – 7.28 (m, 1H, Ar-CH), 7.03 – 6.93 (m, 2H, 2 × Ar-CH), 3.87 (s, 3H, OCH_3), 2.19 (s, 2H, NH_2), 1.68 (s, 6H, $C(CH_3)_2$). **^{13}C NMR** (101 MHz, $CDCl_3$) δ 163.0 (Ar-C_q), 151.4 (Ar-C_q), 140.3 (Ar-C_q), 134.7 (Ar-C_q), 132.7 (Ar-CH), 132.4 (Ar-CH), 129.4 (2 × Ar-CH), 128.7 (Ar-CH), 126.5 (Ar-CH), 114.2 (2 × Ar-CH), 55.6 (OCH_3), 54.2 (C_q), 33.5 ($C(CH_3)_2$). The analytical data (^{1}H, ^{13}C NMR) are in agreement with the reported literature.[2]

2-(2-((4-(Trifluoromethyl)phenyl)sulfonyl)phenyl)propan-2-amine 26

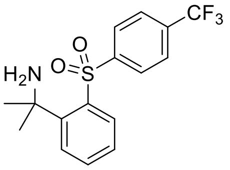

Prepared according to the general procedure using 1-methyl-1-phenylethylamine (122 mg, 0.90 mmol) and sodium 4-(trifluoromethyl)benzenesulfinate (139 mg, 0.60 mmol). Sulfonyl amine **26** was isolated by column chromatography (100% EtOAc to 10% MeOH:EtOAc) as a yellow oil (107 mg, 0.31 mmol, 52%). **R*f*** 0.50 (EtOAc). **^{1}H NMR** (400 MHz, $CDCl_3$) δ 8.28 (d, *J* = 8.1 Hz, 1H, Ar-CH), 7.87 (d, *J* = 8.3 Hz, 2H, 2 × Ar-CH), 7.71 (d, *J* = 8.2 Hz, 2H, 2 × Ar-CH), 7.66 – 7.53 (m, 2H, 2 × Ar-CH), 7.43 (ddd, *J* = 8.4, 6.3, 2.4 Hz, 1H, Ar-CH), 1.80 (s, 2H, NH_2), 1.63 (s, 6H, $C(CH_3)_2$). **^{13}C NMR** (126 MHz, $CDCl_3$) δ 152.4 (Ar-C_q), 149.0 (Ar-C_q), 137.9 (Ar-C_q), 133.6 (Ar-CH), 133.42 (Ar-CH), 133.40 (q, *J* = 33.2 Hz, Ar-C_q), 128.7 (Ar-CH), 126.8 (Ar-CH), 126.2 (2 × Ar-CH), 125.8 (q, *J* = 3.8 Hz, 2 × Ar-CH), 123.3 (q, *J* = 272.8 Hz, CF_3), 54.1 ($\mathit{C}(CH_3)_2$), 34.1 ($C(\mathit{C}H_3)_2$). **^{19}F NMR** (377 MHz, $CDCl_3$) δ −63.0. The analytical data (^{1}H, ^{13}C, ^{19}F NMR) are in agreement with the reported literature.[2]

2-(2-((3,4-Dichlorophenyl)sulfonyl)phenyl)propan-2-amine 27

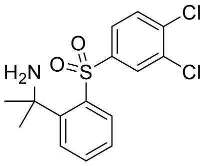

Prepared according to the general procedure using 1-methyl-1-phenylethylamine (122 mg, 0.90 mmol) and sodium 3,4-dichlorobenzenesulfinate (140 mg, 0.60 mmol). Sulfonyl amine **27** was isolated by column chromatography (100% EtOAc to 10% MeOH:EtOAc) as a yellow oil (124 mg, 0.36 mmol, 60%). **R*f*** 0.45 (EtOAc). **^{1}H NMR** (400 MHz, $CDCl_3$) δ 8.22 (dd, *J* = 8.1, 1.4 Hz, 1H, Ar-CH), 7.88 (d, *J* = 2.1 Hz, 1H, Ar-CH), 7.64 – 7.60 (m, 2H, 2 × Ar-CH), 7.57 (dd, *J* = 8.0, 1.4 Hz, 1H, Ar-CH), 7.54 (d, *J* = 8.4 Hz, 1H, Ar-CH), 7.43 (ddd, *J* = 8.4, 6.8, 1.8 Hz, 1H, Ar-CH), 1.90 (s, 2H, NH_2), 1.66 (s, 6H, $C(CH_3)_2$). **^{13}C NMR** (101 MHz, $CDCl_3$) δ 152.2 (Ar-C_q), 144.9 (Ar-C_q), 138.2 (Ar-C_q), 136.6 (Ar-C_q), 133.6 (Ar-CH), 133.3 (Ar-C_q), 133.2 (Ar-CH), 130.7 (Ar-CH), 128.7 (Ar-CH), 128.1 (Ar-CH), 126.8 (Ar-CH), 125.3 (Ar-CH), 54.1 (C_qNH_2), 34.1 ($C(\mathit{C}H_3)_2$). **IR** (film)/cm^{-1} 3394, 2967, 1456, 1370, 1303, 1152, 1031, 818. **HRMS** (TOF—ESI$^+$) m/z calcd. for $C_{15}H_{16}NO_2SCl_2^+$ [M+H]$^+$: 344.0279; found 344.0272.

2-(2-((3,5-Bis(trifluoromethyl)phenyl)sulfonyl)phenyl)propan-2-amine 28

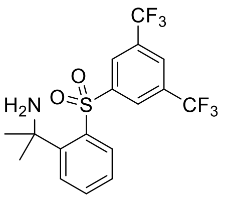

Prepared according to the general procedure using 1-methyl-1-phenylethylamine (122 mg, 0.90 mmol) and sodium 3,5-bis(trifluoromethyl)benzenesulfinate (180 mg, 0.60 mmol). Sulfonyl amine **28** was isolated by column chromatography (100% EtOAc to 10% MeOH:EtOAc) as a yellow oil (152 mg, 0.37 mmol, 62%). **R*f*** 0.65 (EtOAc). **^{1}H NMR** (400 MHz, $CDCl_3$) δ 8.43 (dd, *J* = 8.1, 1.5 Hz, 1H, Ar-CH), 8.20 – 8.16 (m, 2H, 2 × Ar-CH), 7.96 (td, *J* = 1.7, 0.9 Hz, 1H, Ar-CH), 7.64 (ddd, *J* = 8.4, 7.0, 1.5 Hz, 1H, Ar-CH), 7.58 (dd, *J* = 8.0, 1.6 Hz, 1H, Ar-CH), 7.50 (ddd, *J* = 8.5, 7.1, 1.5 Hz, 1H, Ar-CH), 1.71 (s, 2H, NH_2), 1.63 (s, 6H, $C(CH_3)_2$). **^{13}C NMR** (101 MHz, $CDCl_3$) δ 152.1 (Ar-C_q), 148.8 (Ar-C_q), 137.2 (Ar-C_q), 134.2 (Ar-CH), 133.5 (Ar-CH), 132.1 (q, *J* = 34.5 Hz, 2 × Ar-C_q), 129.0 (Ar-CH), 127.2 (Ar-CH), 126.28 – 125.81 (m, 2 × Ar-CH), 124.9 (q, *J* = 3.7 Hz, Ar-CH), 122.6 (q, *J* = 273.1 Hz, 2 × CF_3), 54.2 (C_qNH_2), 34.3($C(\mathit{C}H_3)_2$). **^{19}F NMR** (377 MHz, $CDCl_3$) δ -62.9. **IR** (film)/cm^{-1} 2971, 1359, 1279, 1137, 896, 682, 591. **HRMS** (TOF—ESI$^+$) m/z calcd. for $C_{17}H_{16}NO_2F_6S^+$ [M+H]$^+$: 412.0806; found 412.0798.

2-(2-(Methylsulfonyl)phenyl)propan-2-amine 16

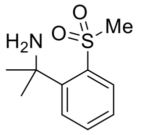

Prepared according to the modified general procedure with 1-methyl-1-phenylethylamine (122 mg, 0.90 mmol) and sodium methanesulfinate (61 mg, 0.60 mmol). All reaction compoents were added to the cathodic chamber of the divided cell. Blank solvent mixture was added to the anodic chamber of the divided cell. nBu$_4$NBF$_4$ (100 mM) was used as supporting electrolyte for electrolysis in the divided cell. Sulfonyl amine **16** was isolated by column chromatography (100% EtOAc to 10% MeOH:EtOAc) as a yellow solid (78 mg, 0.37 mmol, 61%). **R*f*** 0.16 (EtOAc). **Melting Point** 58 – 60 °C (EtOAc/MeOH ppt). **^{1}H NMR** (400 MHz, CDCl$_3$) δ 8.25 (dd, *J* = 8.7, 1.1 Hz, 1H, Ar-CH), 7.62 – 7.45 (m, 2H, 2 × Ar-CH), 7.40 (ddd, *J* = 8.4, 6.3, 2.3 Hz, 1H, Ar-CH), 3.51 (s, 3H, SO$_2$CH$_3$), 2.34 (s, 2H, NH$_2$), 1.69 (s, 6H, C(CH$_3$)$_2$). **^{13}C NMR** (101 MHz, CDCl$_3$) δ 151.0 (Ar-C$_q$), 139.8 (Ar-C$_q$), 133.2 (Ar-CH), 132.2 (Ar-CH), 128.2 (Ar-CH), 126.9 (Ar-CH), 54.1 (NH$_2$*C*(CH$_3$)$_2$), 46.9 (SO$_2$CH$_3$), 34.9 (NH$_2$C(*C*H$_3$)$_2$). The analytical data (^{1}H, ^{13}C NMR) are in agreement with the reported literature.[2]

2-Electrodes divided electrochemical cell

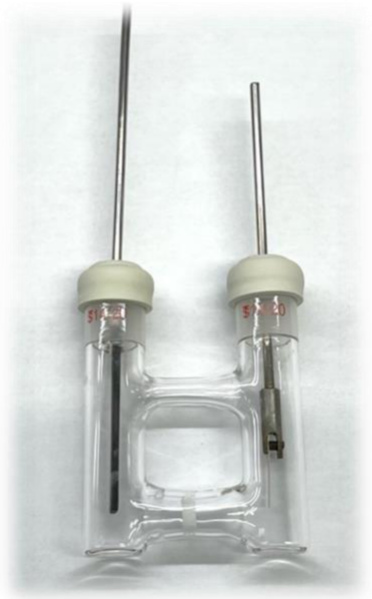

Electrolysis was carried out in a divided electrochemical cell sealed with a subaseal with graphite and platinum electrodes connected with stainless steel electrode holders. The diameter of each chamber of the electrochemical cell was 11 mm are separated with a glass frit.

- Graphite anode: Total surface area submerged in solution – 2.16 cm^2.
- Platinum cathode: Total surface area submerged in solution – 1.05 cm^2.

Electrolysis was carried out with TTi MX100QP quad output multi-range DC power supply under constant current conditions.

Cyclic Voltammetry

Cyclic voltammograms (IUPAC convention) were recorded at room temperature using a Metrohm Autolab PGSTAT204 workstation. Data analysis was performed with Nova 2.0 software. A three-electrode undivided electrochemical cell was employed, comprising a glassy carbon disc electrode (2 mm diameter) as the working electrode, a platinum wire as the counter electrode, and a silver wire as the pseudo-reference electrode.

Before each measurement, the glassy carbon electrode was polished on a polishing pad with a water-alumina slurry using figure-eight motions, then rinsed with deionized water and acetone. The solution of interest was sparged with argon for 2 minutes before data collection. Unless otherwise stated, the scan direction was initiated in the positive direction from 0 V. Ferrocene was used as an internal standard after each measurement, and all potentials were referenced to the ferrocenium/ferrocene redox couple. The current was reported in μA, and applied voltages were corrected using positive feedback iR compensation.

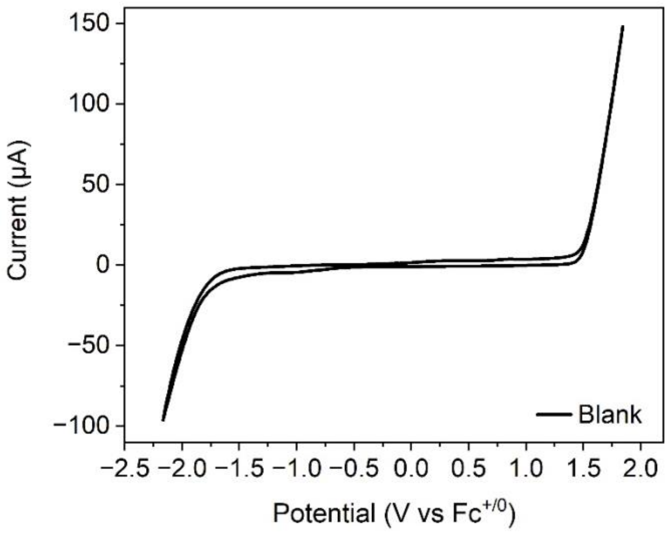

Figure S2. Cyclic voltammogram of the solvent mixture. Conditions: $n\mathrm{Bu_4BF_4}$ (0.10 M in 3:1 HFIP:NMP). Purged with Ar. Scan rate: 100 mVs^{-1}. Scan direction: Positive direction from 0 V.

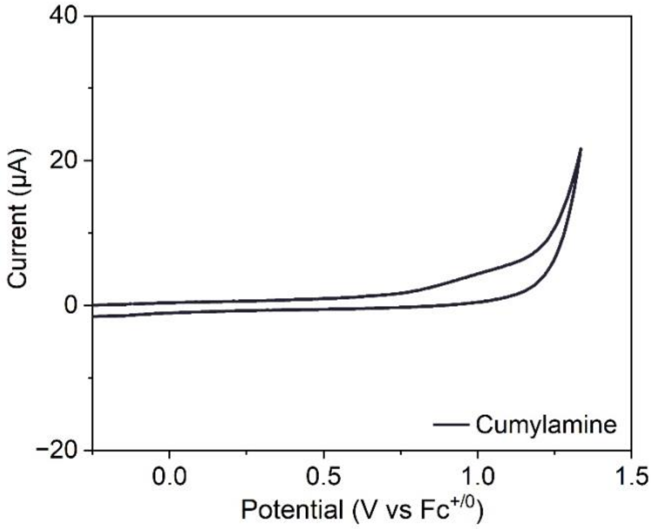

Figure S3. Cyclic voltammogram of cumylamine (20 mM). Conditions: $n\mathrm{Bu_4BF_4}$ (0.10 M in 3:1 HFIP:NMP). Purged with Ar. Scan rate: 100 mVs^{-1}. Scan direction: Positive direction from 0 V.

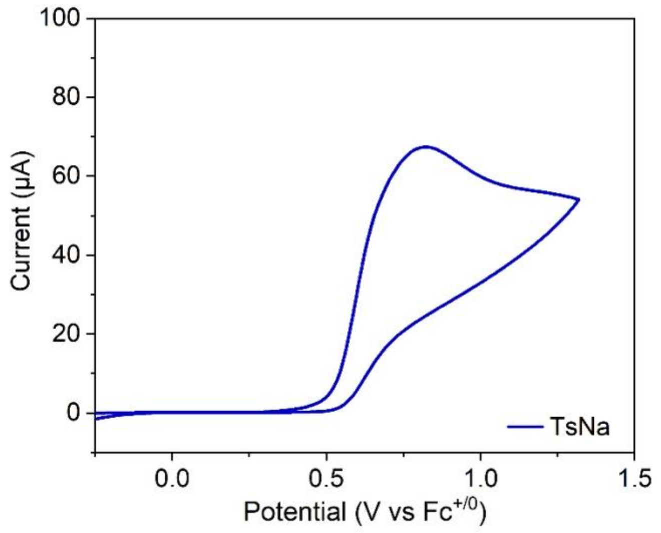

Figure S4. Cyclic voltammogram of TsNa (20 mM). Conditions: *n*Bu_4BF_4 (0.10 M in 3:1 HFIP:NMP). Purged with Ar. Scan rate: 100 mVs^{-1}. Scan direction: Positive direction from 0 V.

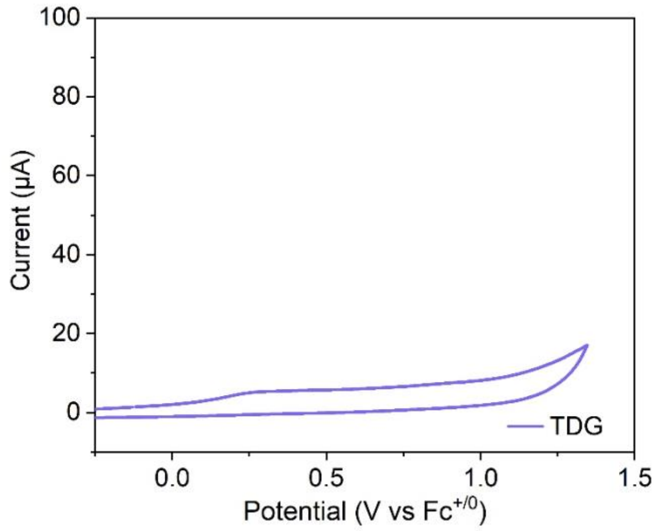

Figure S5. Cyclic voltammogram of 2-hydroxynicotinaldehyde (20 mM). Conditions: *n*Bu_4BF_4 (0.10 M in 3:1 HFIP:NMP). Purged with Ar. Scan rate: 100 mVs^{-1}. Scan direction: Positive direction from 0 V.

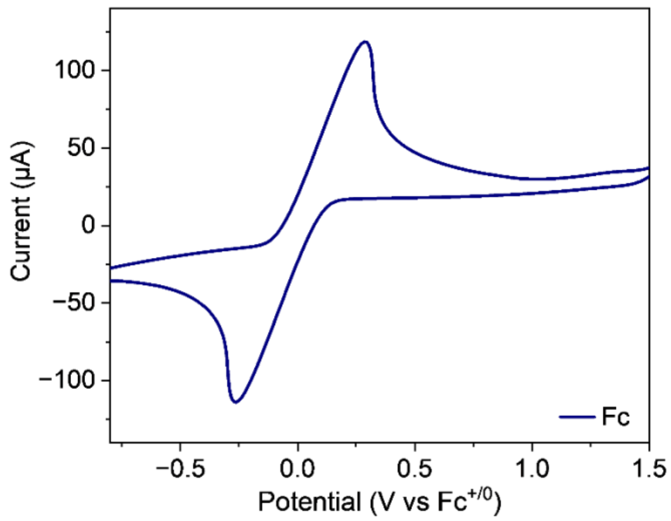

Figure S6. Cyclic voltammogram of Fc (20 mM). Conditions: *n*Bu_4BF_4 (0.10 M in 3:1 HFIP:NMP). Purged with Ar. Scan rate: 100 mVs^{-1}. Scan direction: Positive direction from -0.8 V.

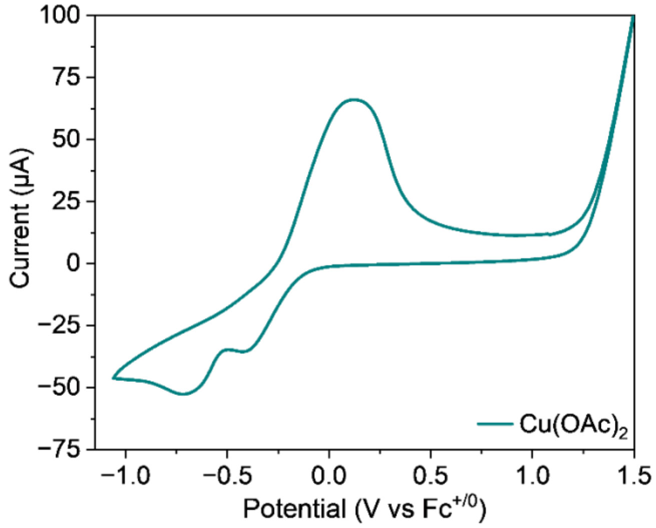

Figure S7. Cyclic voltammogram of $Cu(OAc)_2$ (20 mM). Conditions: *n*Bu_4BF_4 (0.10 M in 3:1 HFIP:NMP). Purged with Ar. Scan rate: 100 mVs^{-1}. Scan direction: Positive direction from 1 V.

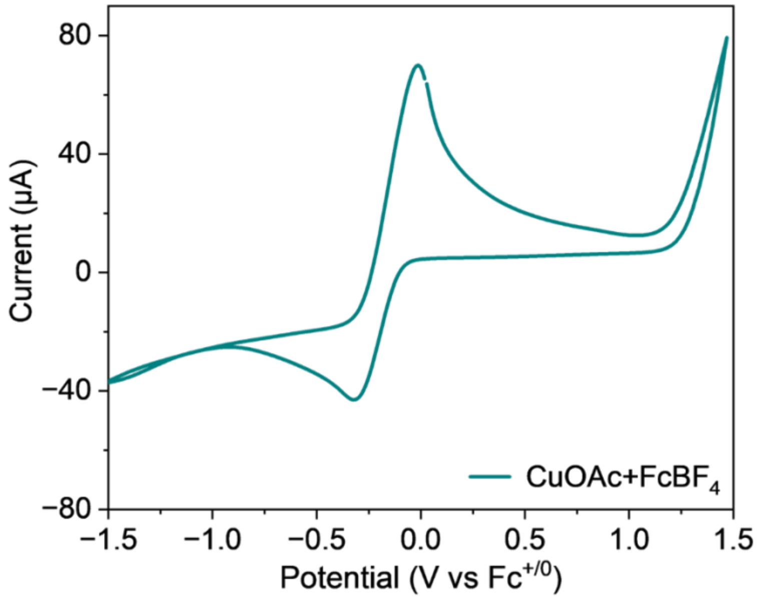

Figure S8. Cyclic voltammogram of CuOAc (20 mM) and $FcBF_4$ (20 mM). Conditions: *n*Bu_4BF_4 (0.10 M in 3:1 HFIP:NMP). Purged with Ar. Scan rate: 100 mVs^{-1}. Scan direction: Positive direction from 0 V.

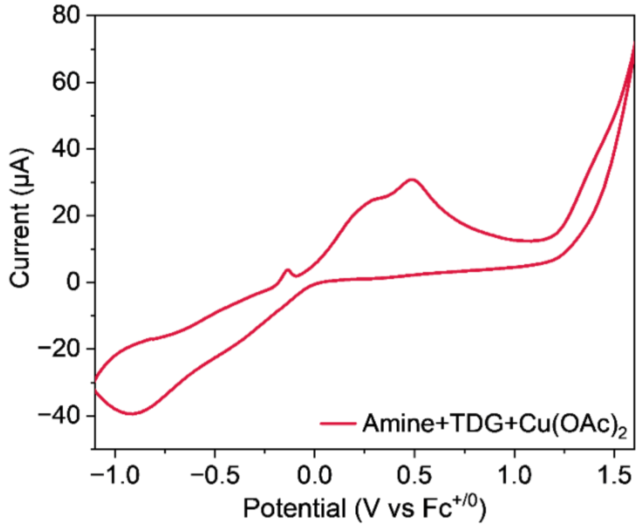

Figure S9. Cyclic voltammogram of $Cu(OAc)_2$ (20 mM) with cumylamine (20 mM) and 2-hydroxynicotinaldehyde (20 mM). Conditions: *n*Bu_4BF_4 (0.10 M in 3:1 HFIP:NMP). Purged with Ar. Scan rate: 100 mVs^{-1}. Scan direction: Positive direction from -0.8 V.

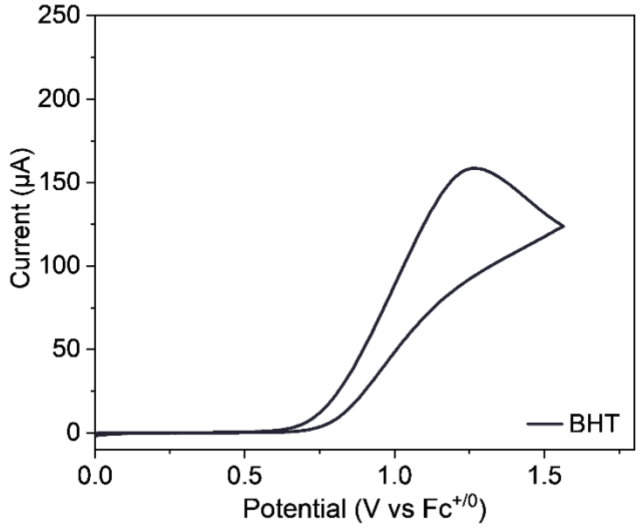

Figure S10. Cyclic voltammogram of BHT (20 mM). Conditions: nBu$_4$BF$_4$ (0.10 M in 3:1 HFIP:NMP). Purged with Ar. Scan rate: 100 mVs^{-1}. Scan direction: Positive direction from -0.8 V.

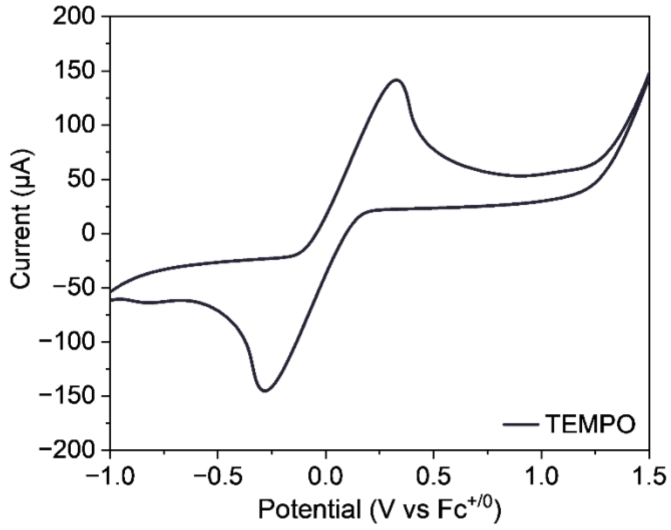

Figure S11. Cyclic voltammogram of TEMPO (20 mM). Conditions: nBu$_4$BF$_4$ (0.10 M in 3:1 HFIP:NMP). Purged with Ar. Scan rate: 100 mVs^{-1}. Scan direction: Positive direction from -0.8 V.

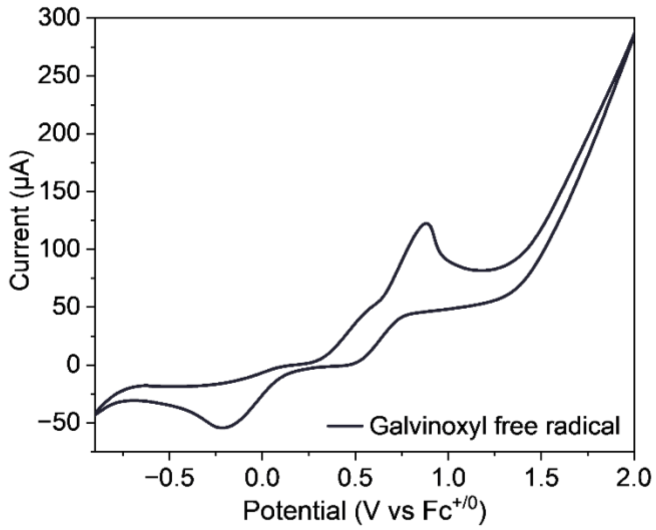

Figure S12. Cyclic voltammogram of galvinoxyl free radical (20 mM). Conditions: *n*Bu_4BF_4 (0.10 M in 3:1 HFIP:NMP). Purged with Ar. Scan rate: 100 mVs^{-1}. Scan direction: Positive direction from -0.8 V.

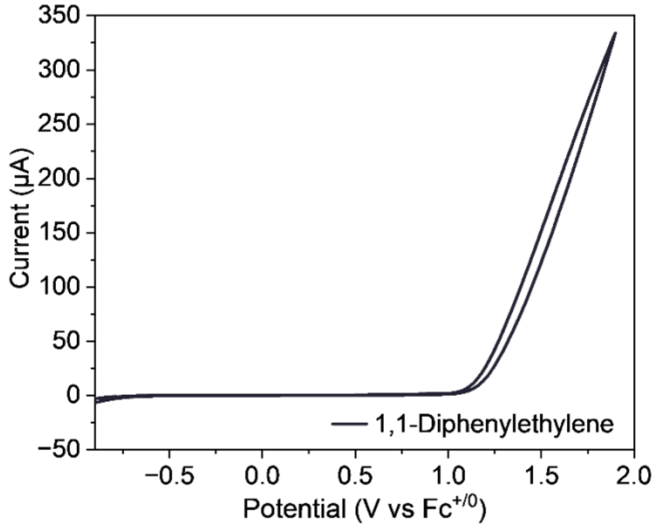

Figure S13. Cyclic voltammogram of 1,1-diphenylethylene (20 mM). Conditions: *n*Bu_4BF_4 (0.10 M in 3:1 HFIP:NMP). Purged with Ar. Scan rate: 100 mVs^{-1}. Scan direction: Positive direction from -0.8 V.

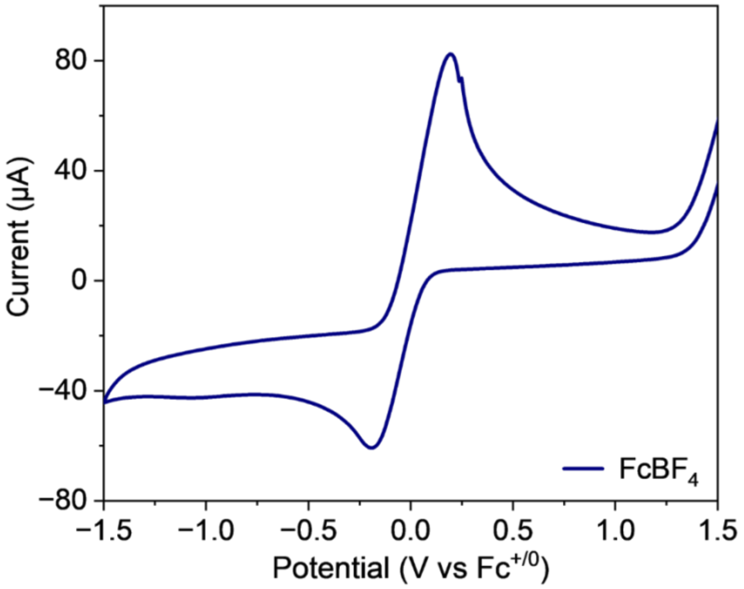

Figure S14. Cyclic voltammogram of $FcBF_4$ (20 mM). Conditions: *n*Bu_4BF_4 (0.10 M in 3:1 HFIP:NMP). Purged with Ar. Scan rate: 100 mVs^{-1}. Scan direction: Positive direction from 0 V.

Monitoring Anodic Potential

The operating potential of the anode was recorded using a Metrohm Autolab PGSTAT204 workstation. Electrolysis was carried out with a three-electrode undivided electrochemical cell with graphite as the anode, a platinum sheet as the cathode, and a silver wire as the pseudo-reference electrode. The potential was then referenced to the ferrocenium/ferrocene redox couple.

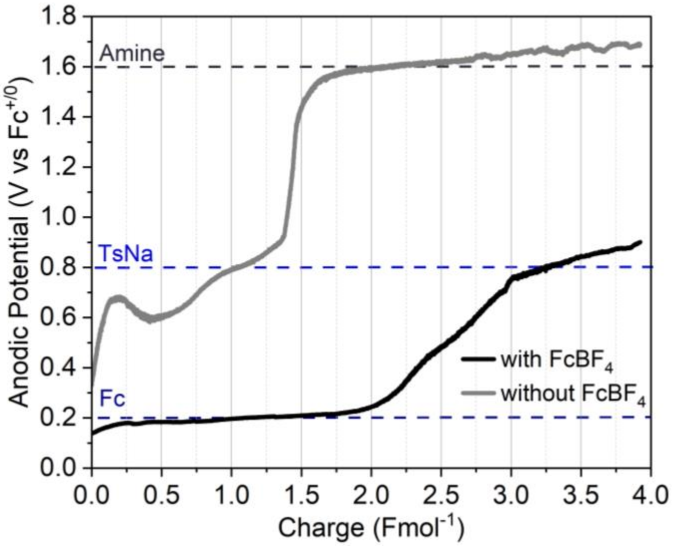

Figure S15. Anodic potential over time.

3-Electrodes undivided electrochemical cell

Since the reaction was performed in a sealed undivided cell under elevated temperature and pressure, the use of more stable regular organic reference electrode was challenging as the reaction temperature is beyond the boiling point of the solvent in those reference electrode.

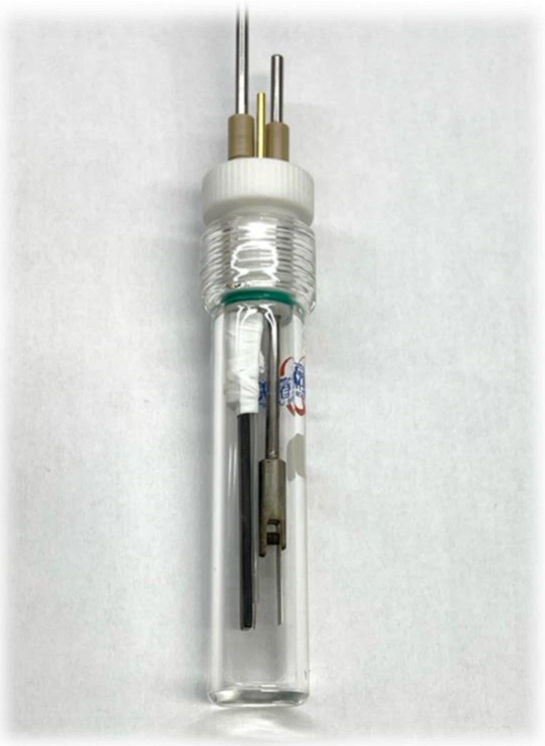

Electrolysis was carried out in an undivided electrochemical cell sealed with a PTFE cap with graphite, silver wire and platinum electrodes. The diameter of the electrochemical cell was 20 mm and the distance between the anode and cathode was 4 mm. The silver wire reference electrode was insulated with a PTFE flow tubing and wrapped around the graphite anode with PTFE tape, the distance between the anode and reference electrode was 1 mm.

- Graphite anode: Total surface area submerged in solution – 2.16 cm^2.
- Platinum cathode: Total surface area submerged in solution – 1.05 cm^2.
- Silver wire reference electrode – 10 mm submerged in solution.

Electrolysis was carried out with Metrohm Autolab PGSTAT204 workstation under constant current condition and the operating potential was recorded every 10s.

Monitoring Cell Potential

The cell potential was recorded by TTi MX100QP quad output multi-range DC power supply. Electrolysis was carried out with a two-electrode undivided electrochemical cell with graphite as the anode and platinum sheet as the cathode. The cell potential was recorded every 10 seconds for 21 h. The electrolysis was carried out under the optimized conditions in HFIP:NMP (3:1) (Entry 1, Table 1) and HFIP (Entry 8, Table 1).

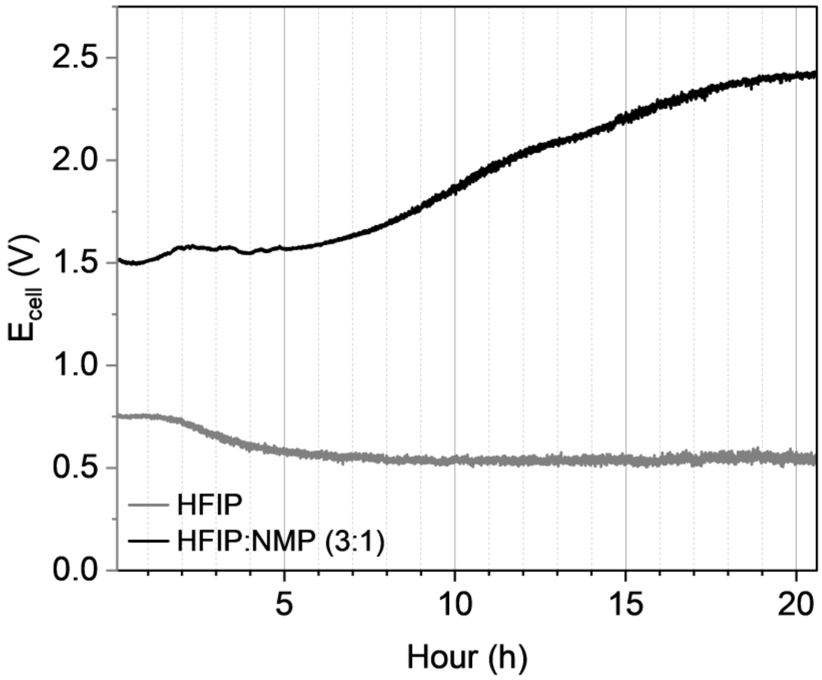

Figure S16. Cell potential over time in different solvent system.

^{1}H and ^{13}C Spectra of Selected Compounds

2-(2-Methoxy-6-tosylphenyl)propan-2-amine 4

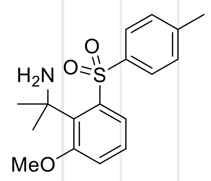

4
^{1}H NMR
(500 MHz, $CDCl_3$)

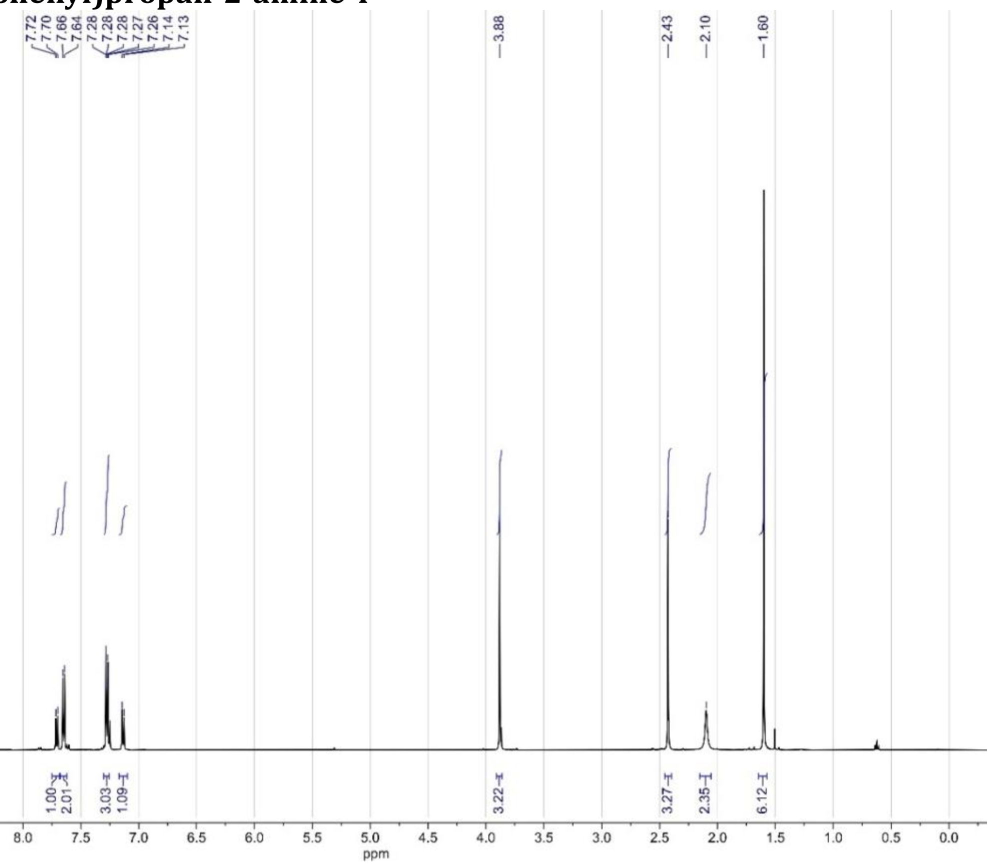

4
^{13}C NMR
(126 MHz, $CDCl_3$)

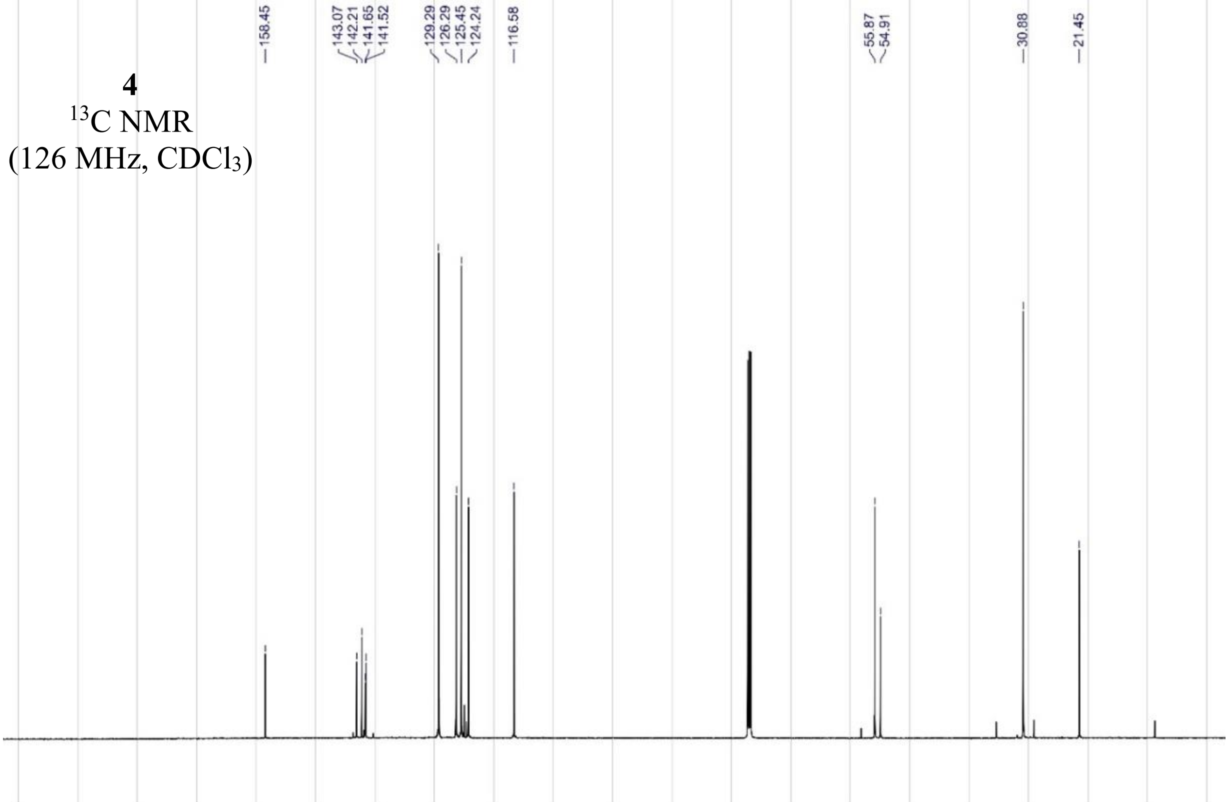

2-(5-Methoxy-2-tosylphenyl)propan-2-amine 5

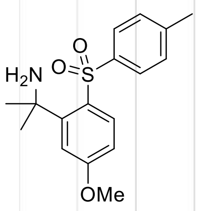

5
^{1}H NMR
(400 MHz, $CDCl_3$)

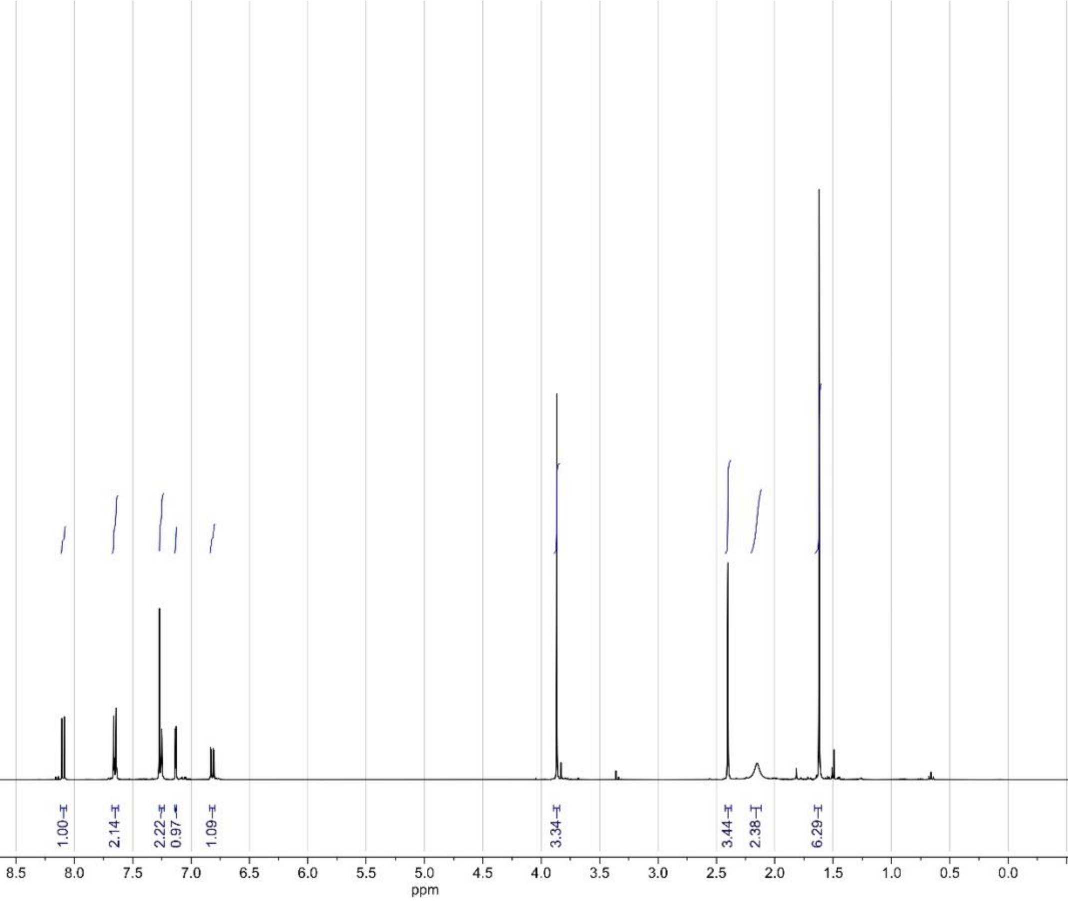

5
^{13}C NMR
(101 MHz, $CDCl_3$)

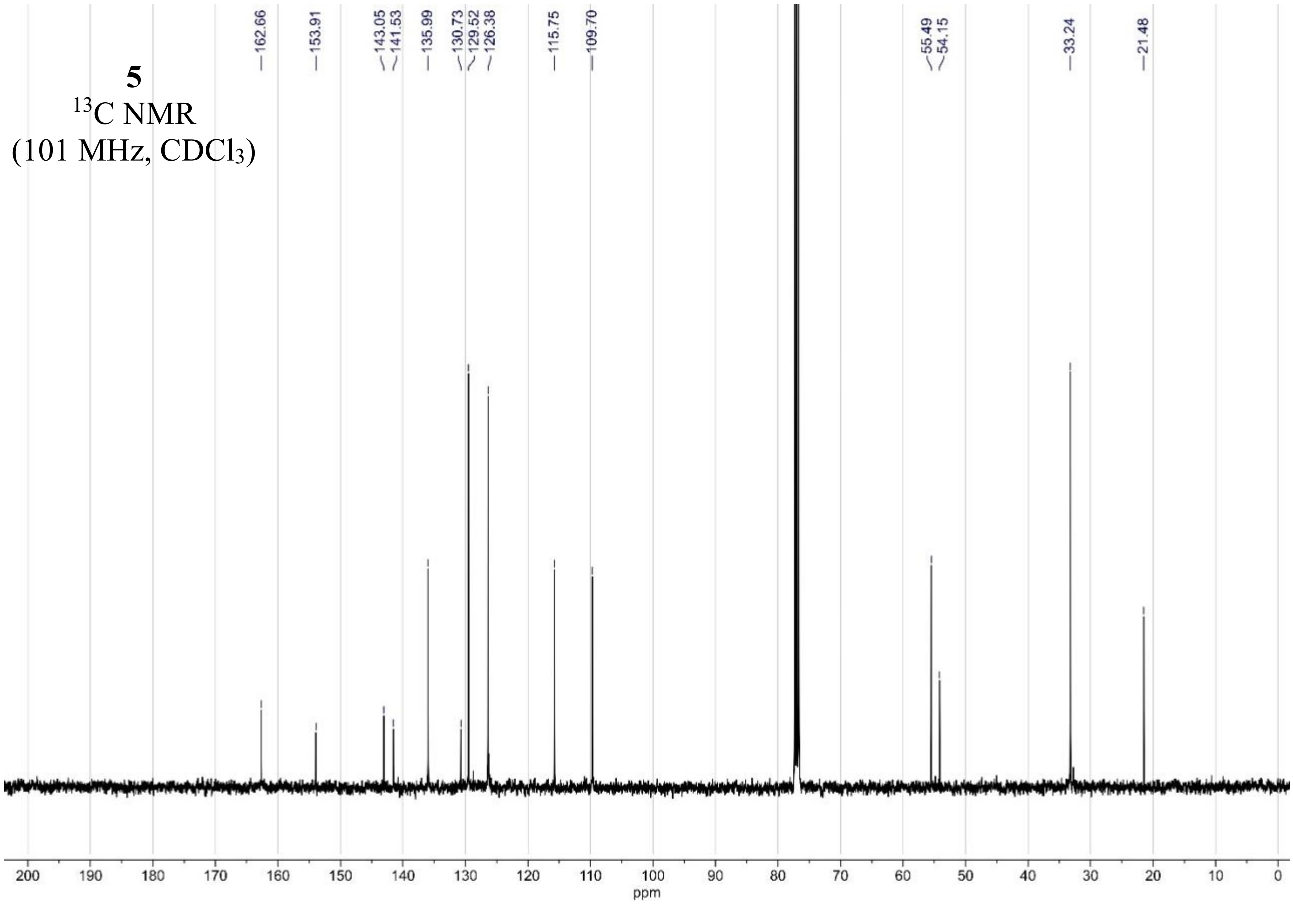

2-(3-Methoxy-2-tosylphenyl)propan-2-amine 5'

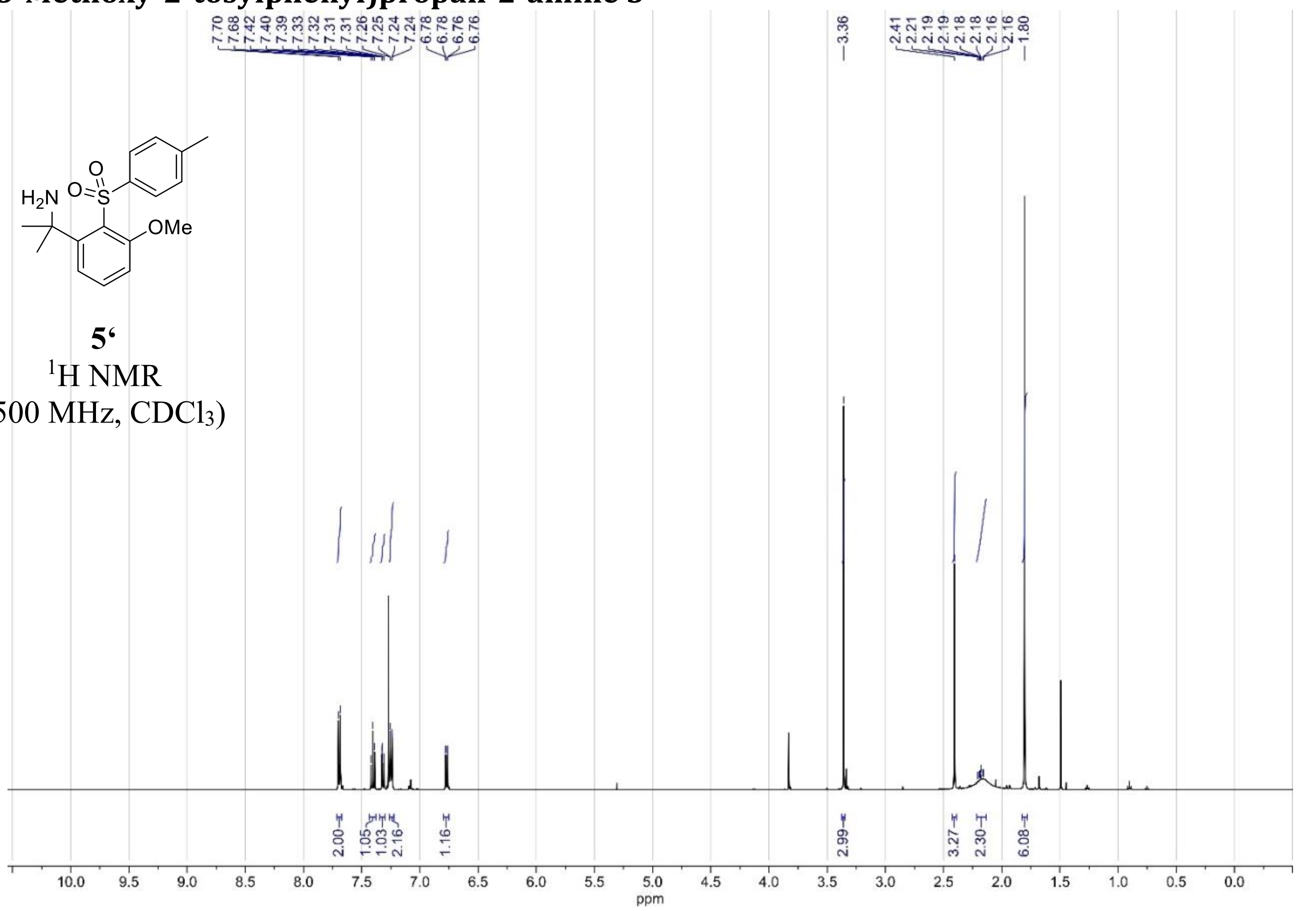

5'
^{1}H NMR
(500 MHz, $CDCl_3$)

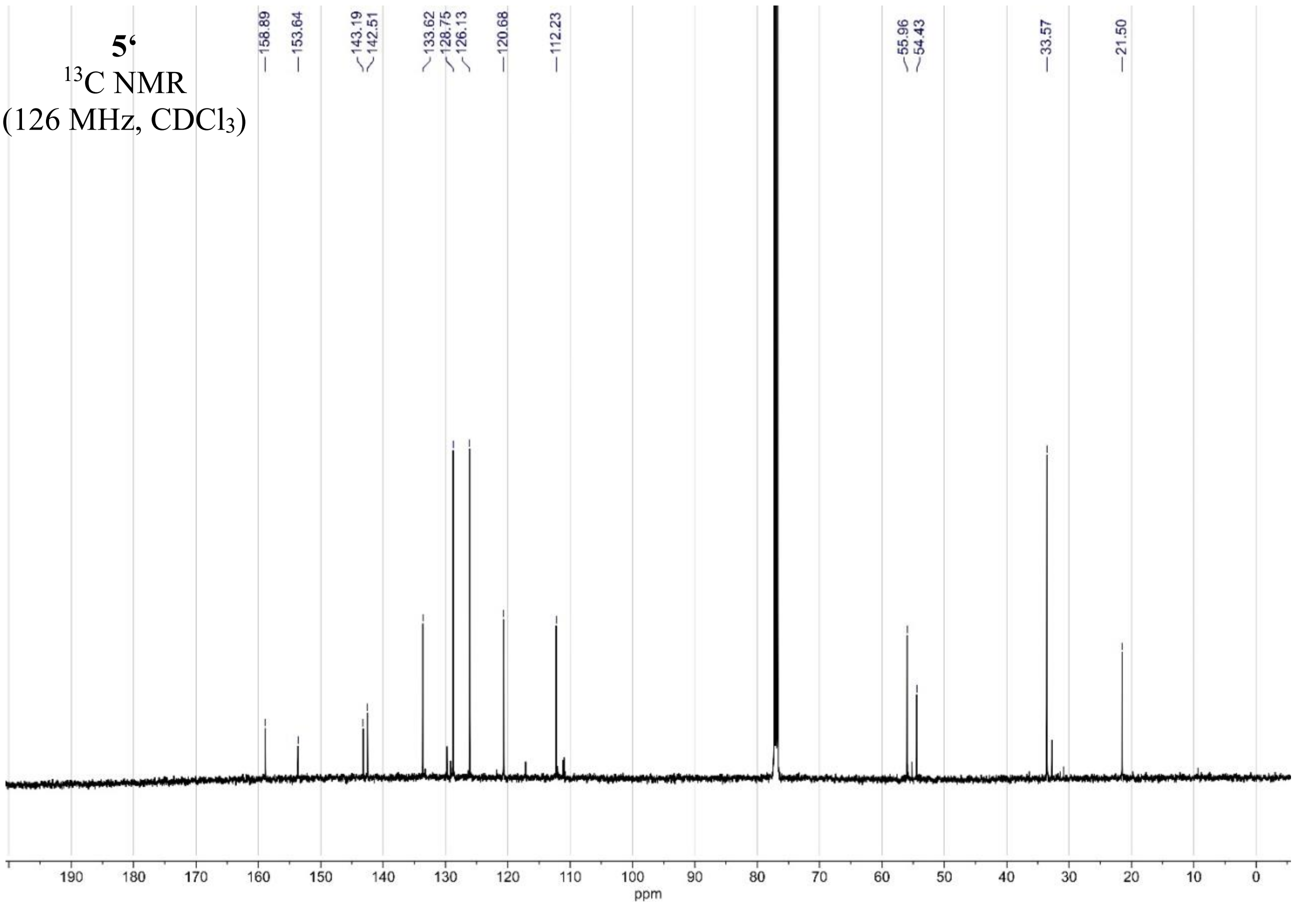

5'
^{13}C NMR
(126 MHz, $CDCl_3$)

2-(4-Methoxy-2-tosylphenyl)propan-2-amine 6

6
^{1}H NMR
(500 MHz, $CDCl_3$)

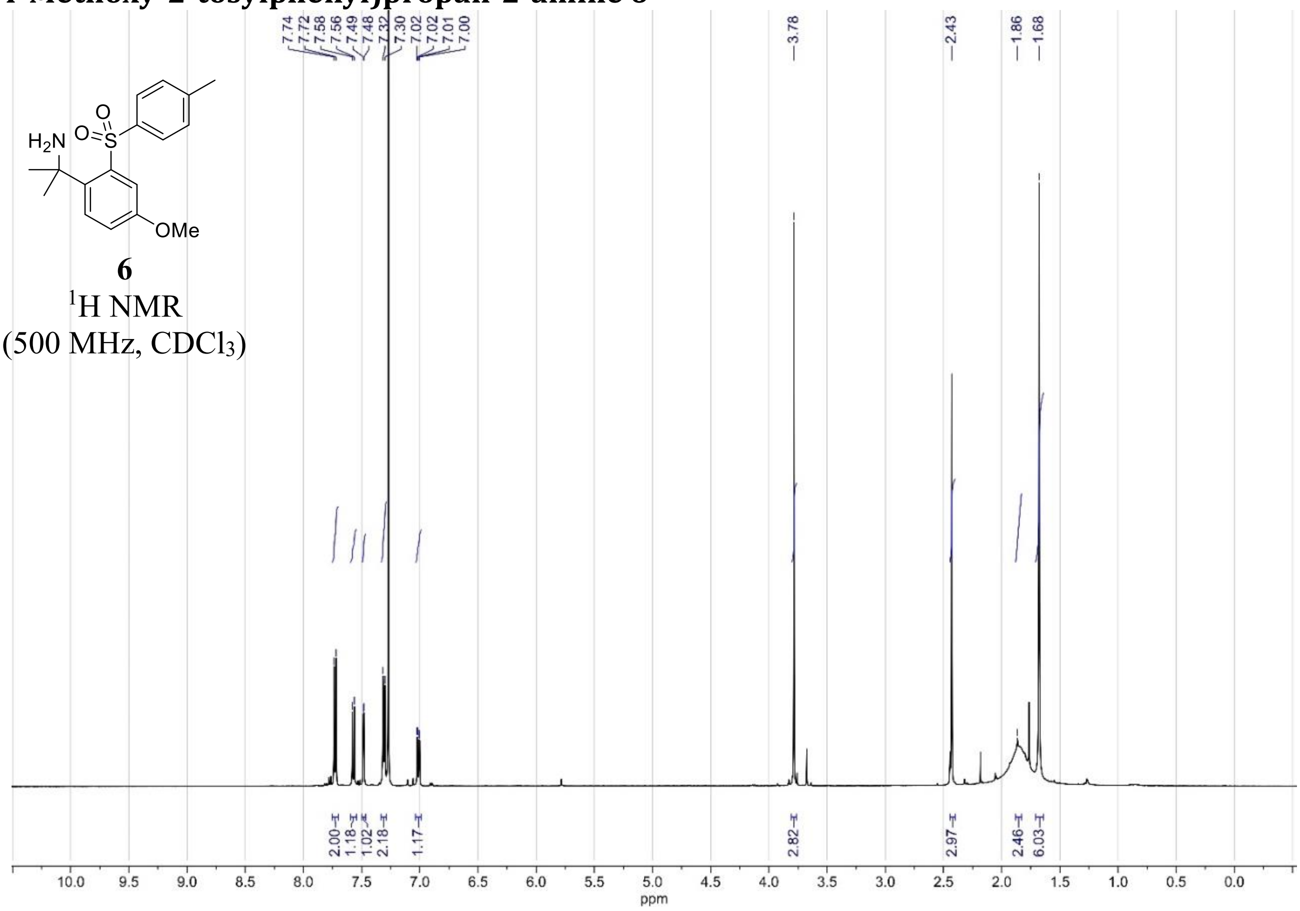

6
^{13}C NMR
(126 MHz, $CDCl_3$)

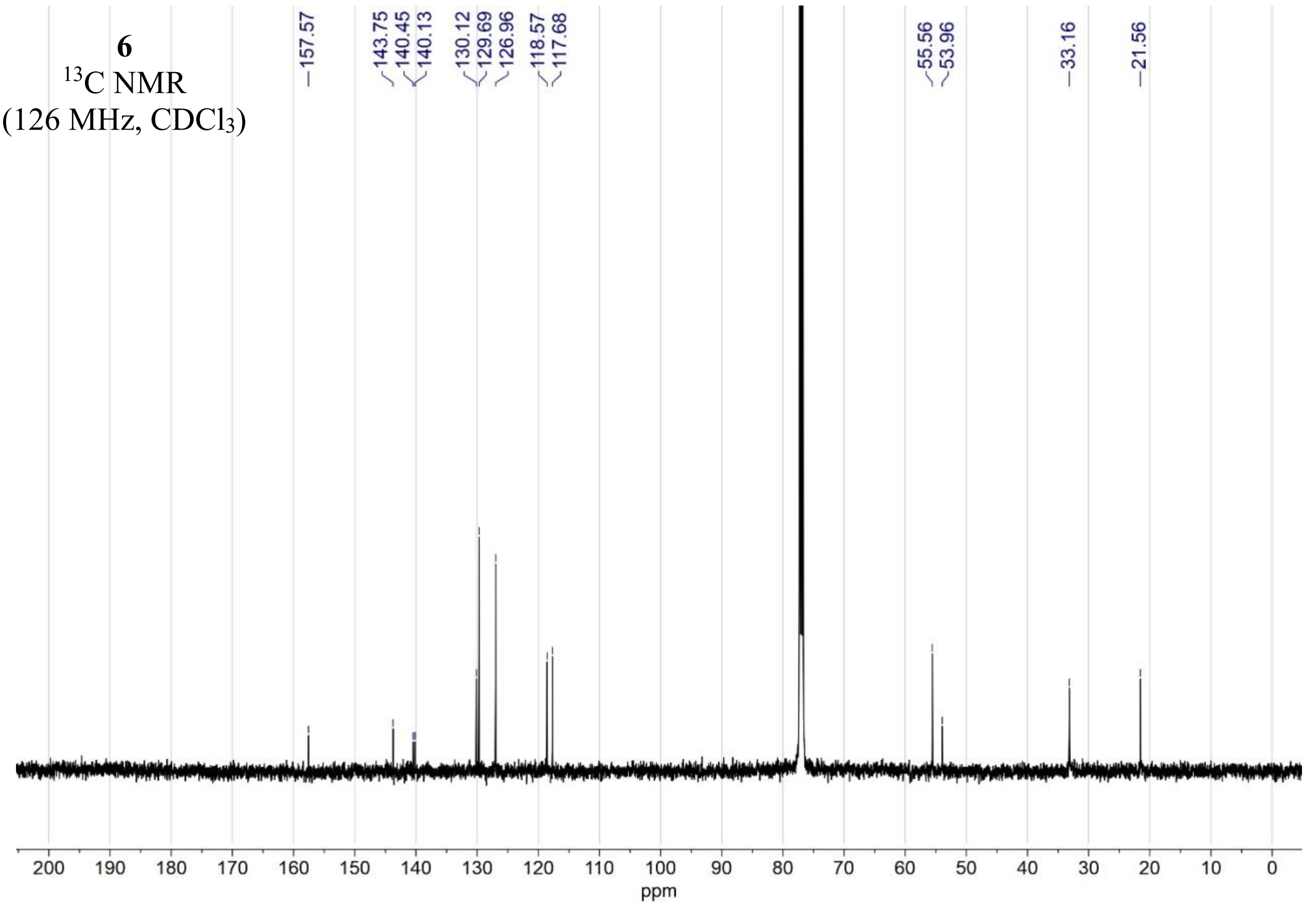

2-(3,4,5-Trimethoxy-2-tosylphenyl)propan-2-amine 7

7
^{1}H NMR
(500 MHz, $CDCl_3$)

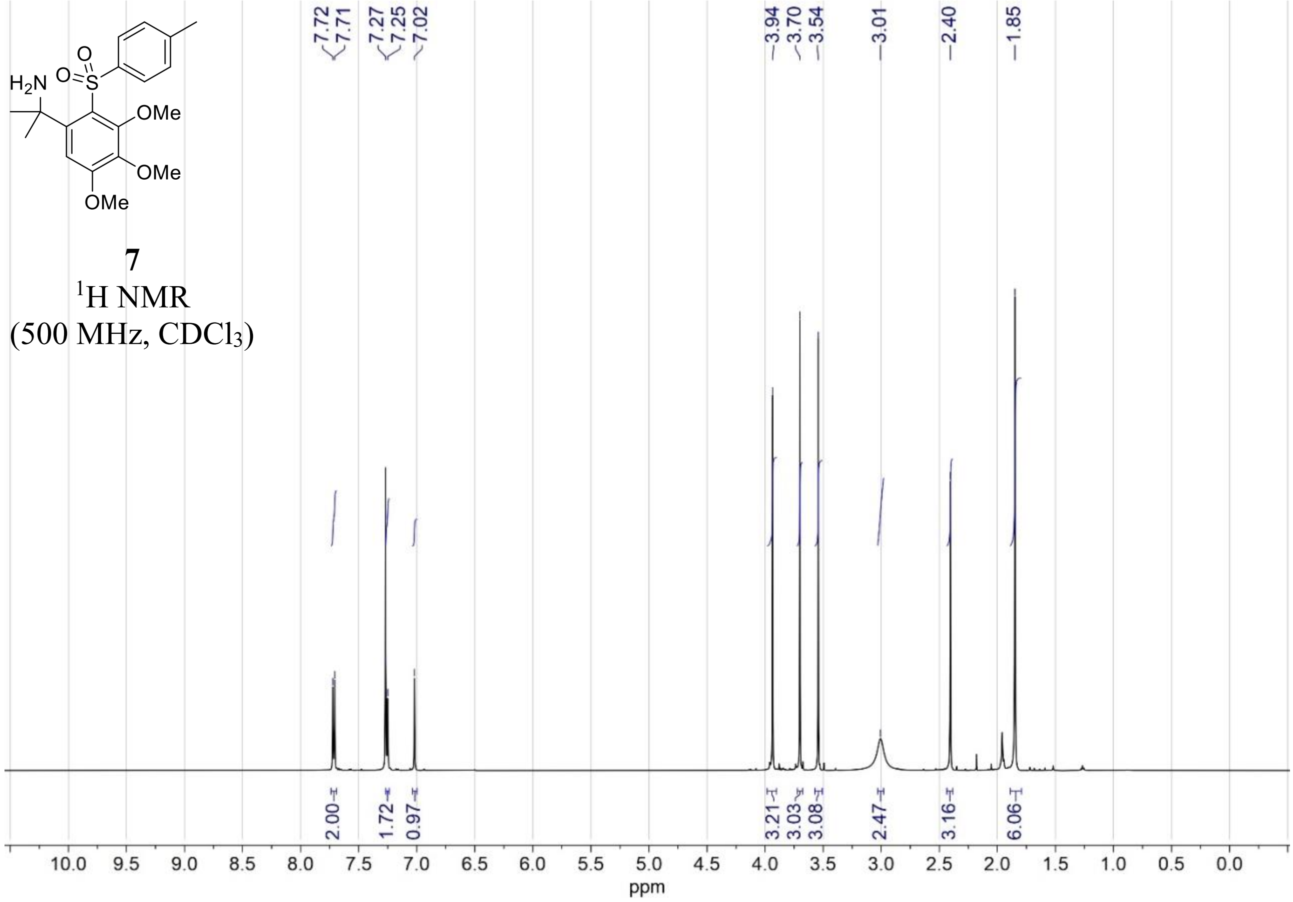

7
^{13}C NMR
(126 MHz, $CDCl_3$)

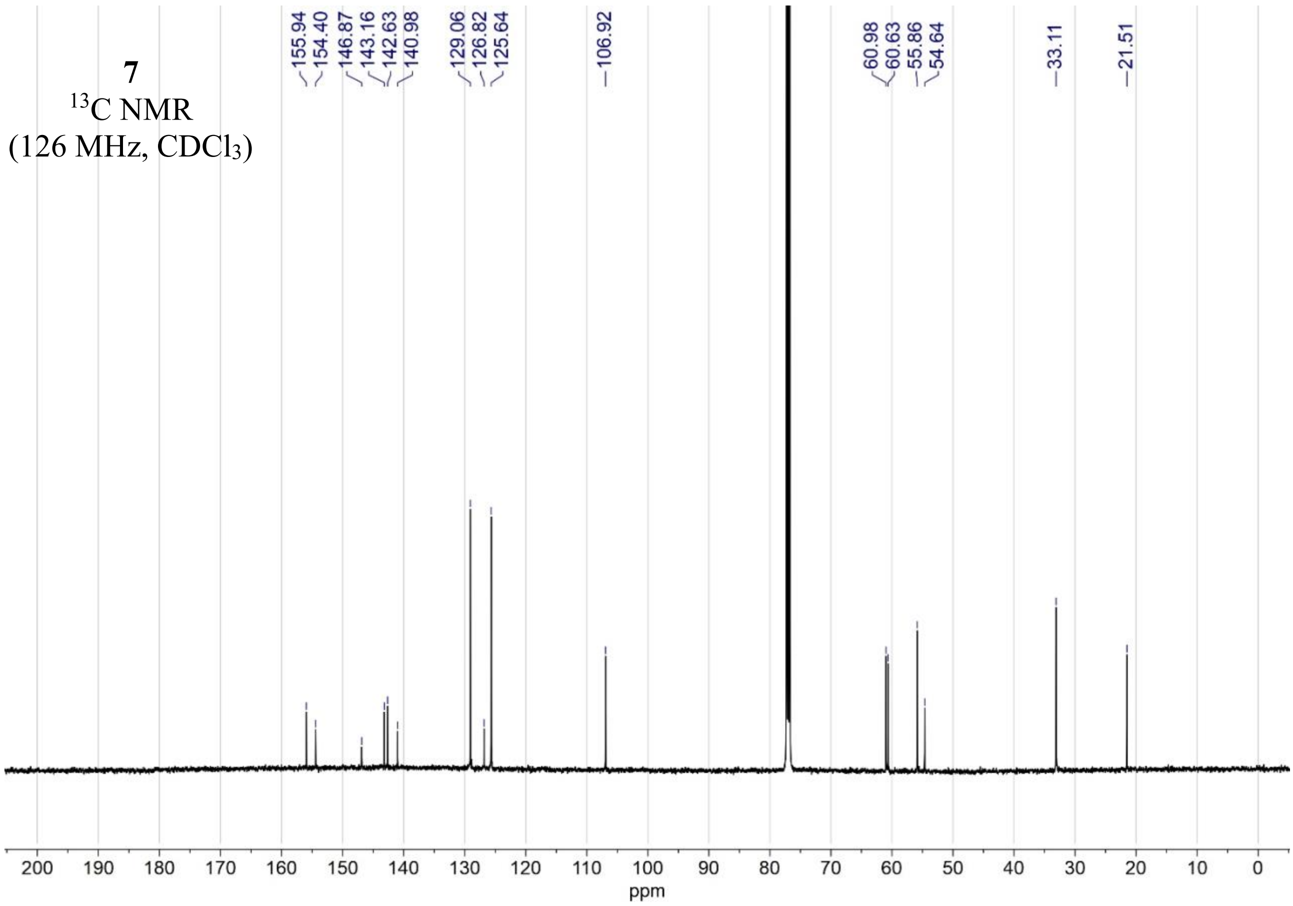

1-(2-Tosylphenyl)cyclopentan-1-amine 8

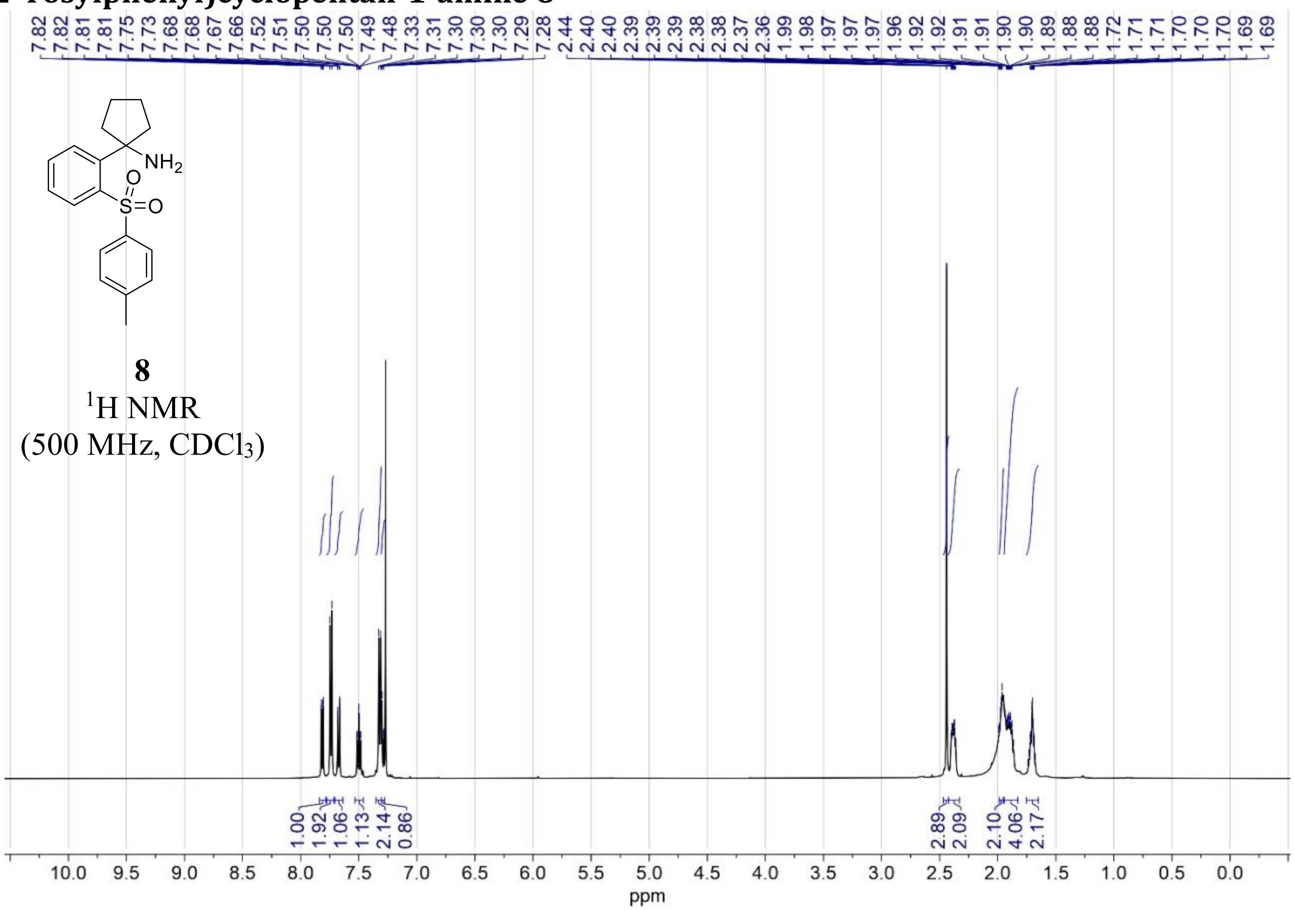

8
^{1}H NMR
(500 MHz, $CDCl_3$)

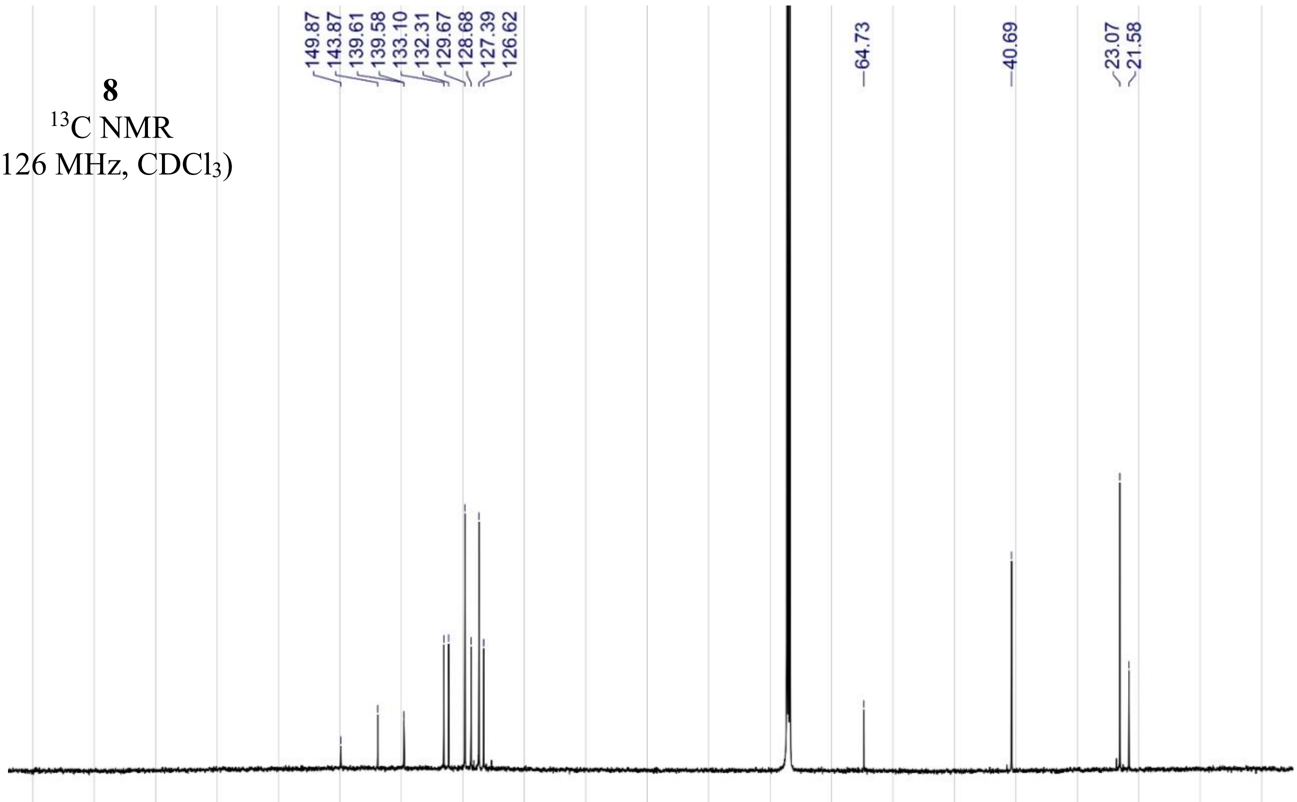

8
^{13}C NMR
(126 MHz, $CDCl_3$)

2-(4-Fluoro-2-tosylphenyl)propan-2-amine 9

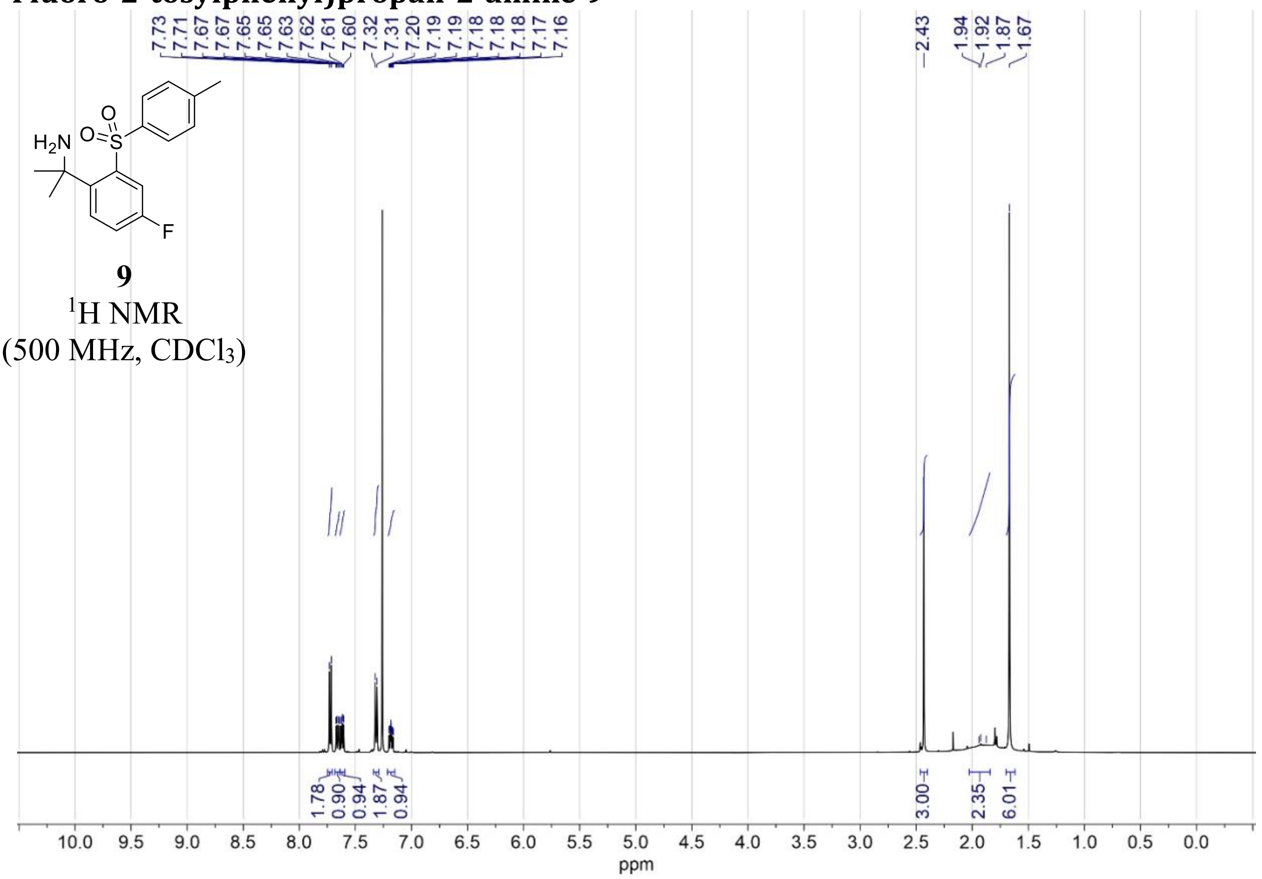

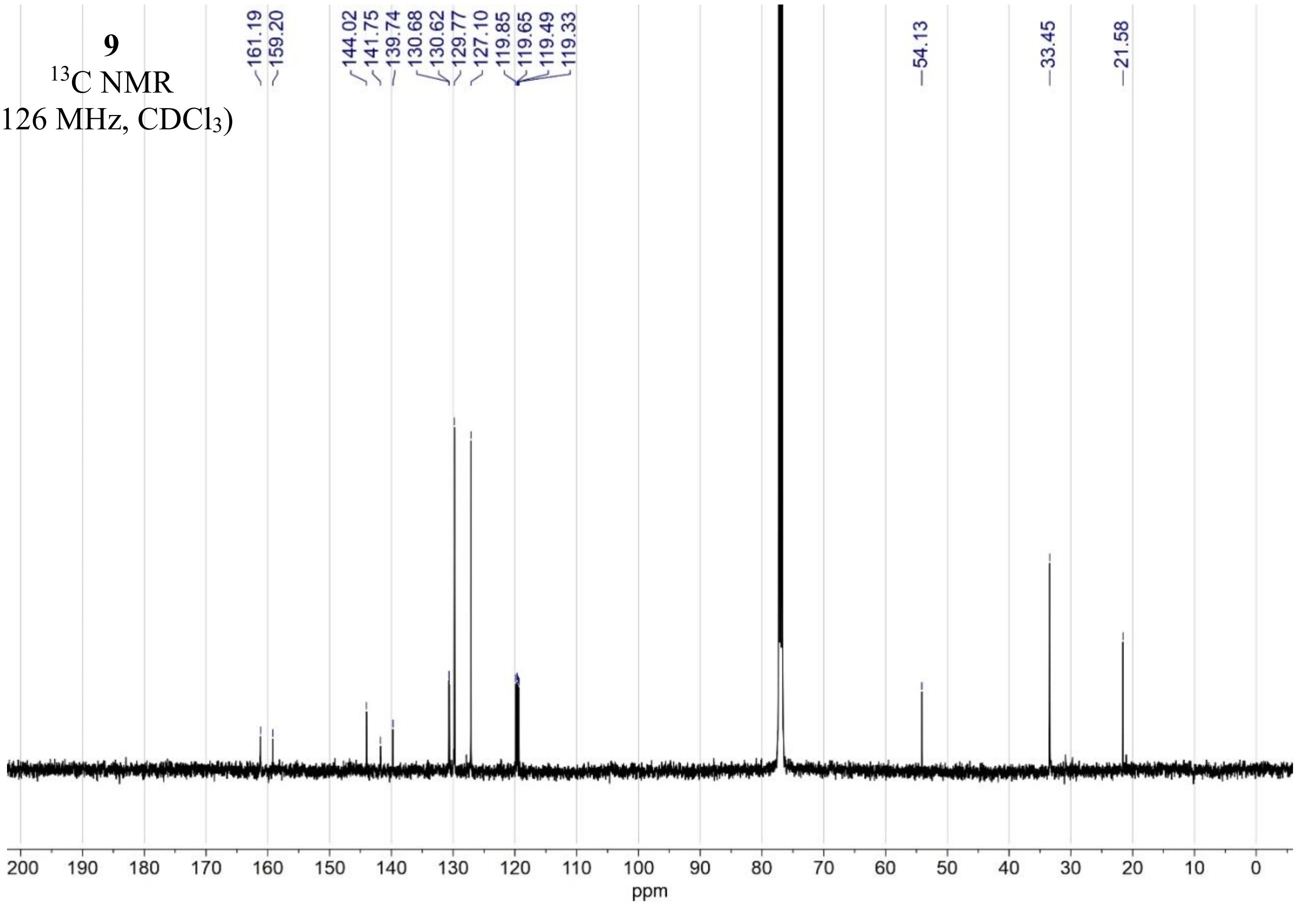

9
^{19}F NMR
(377 MHz, $CDCl_3$)

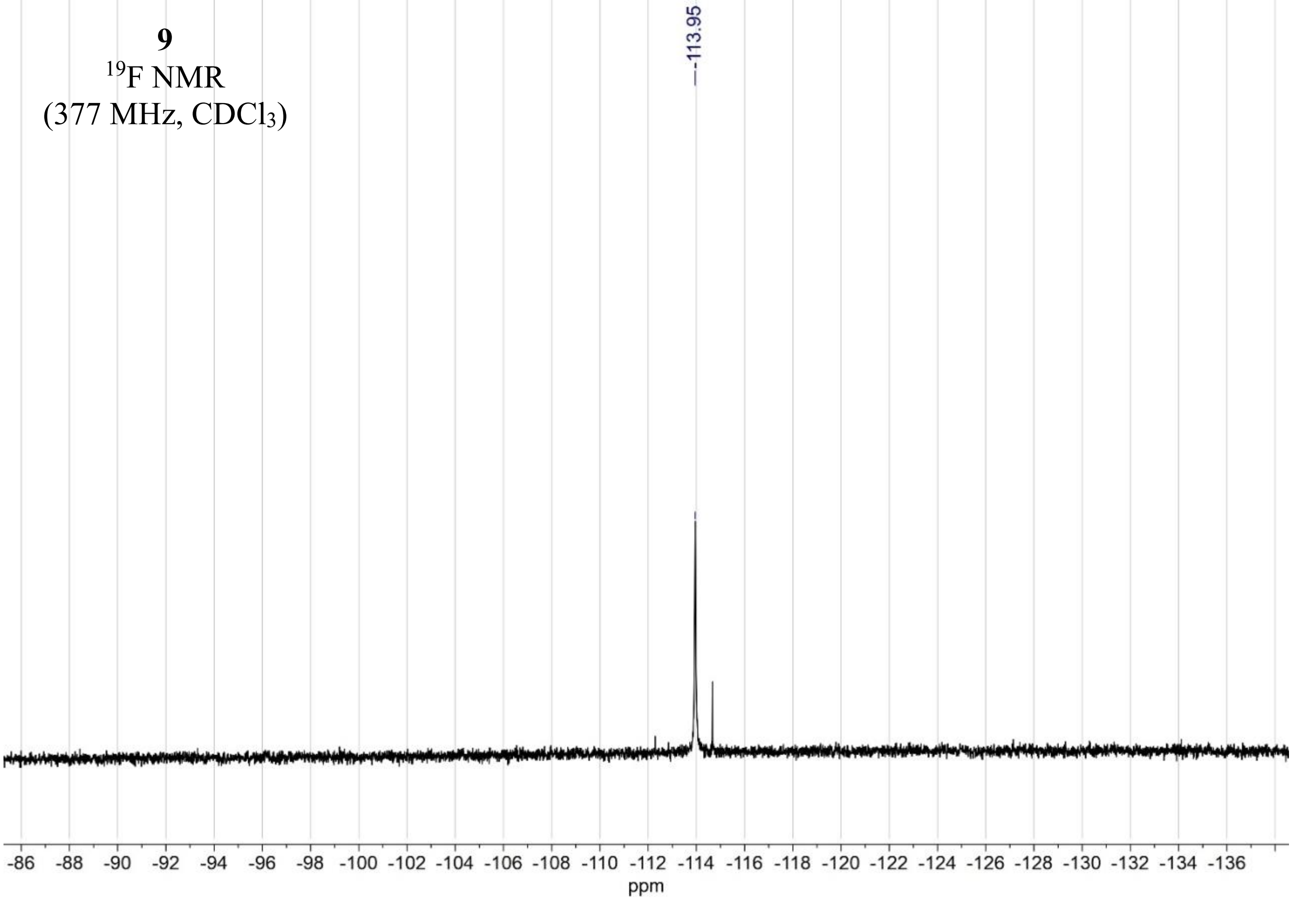

2-(4-Chloro-2-tosylphenyl)propan-2-amine 10

10
^{1}H NMR
(500 MHz, $CDCl_3$)

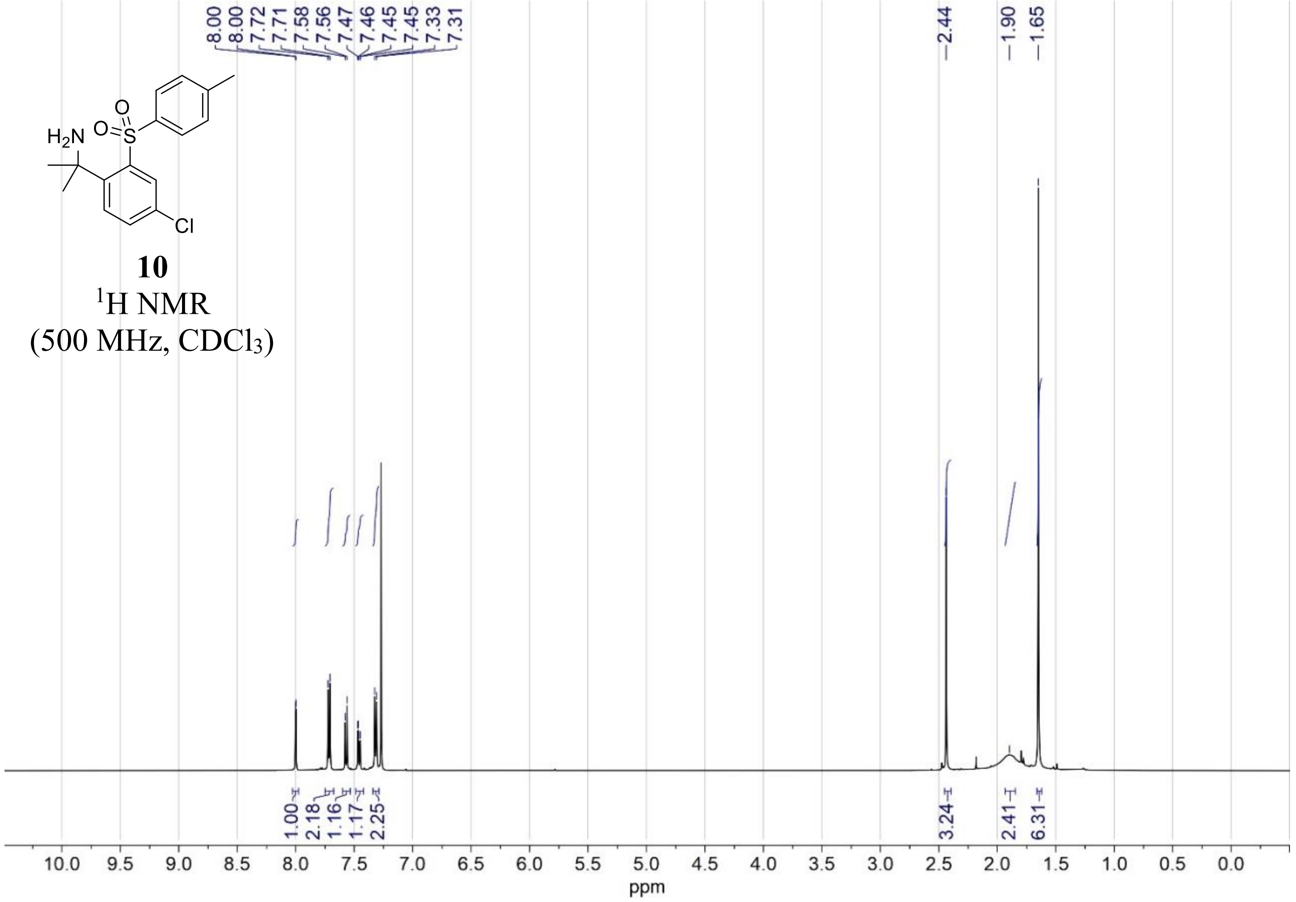

10
^{13}C NMR
(126 MHz, $CDCl_3$)

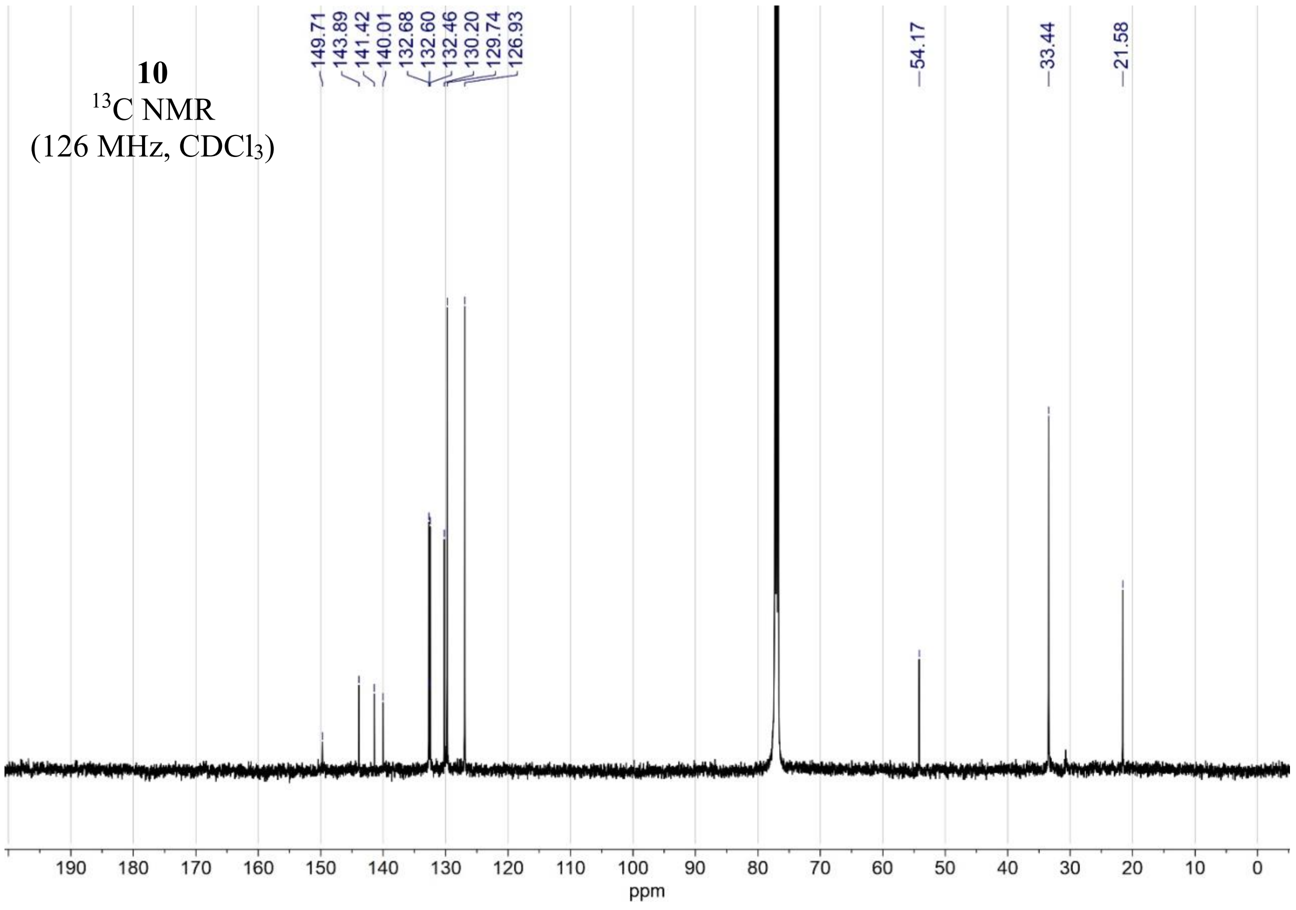

2-(4-Bromo-2-tosylphenyl)propan-2-amine 11

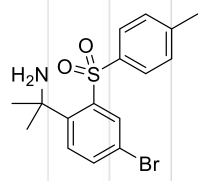

11
^{1}H NMR
(500 MHz, $CDCl_3$)

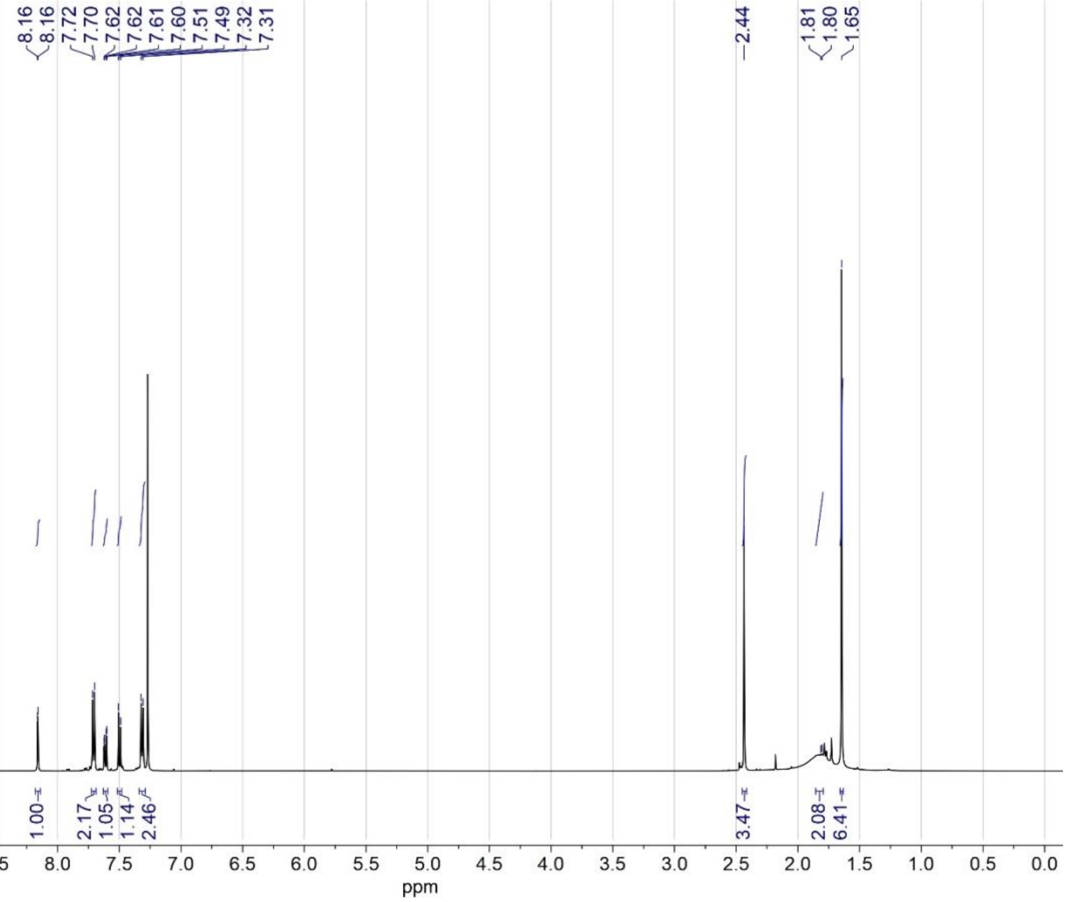

11
^{13}C NMR
(126 MHz, $CDCl_3$)

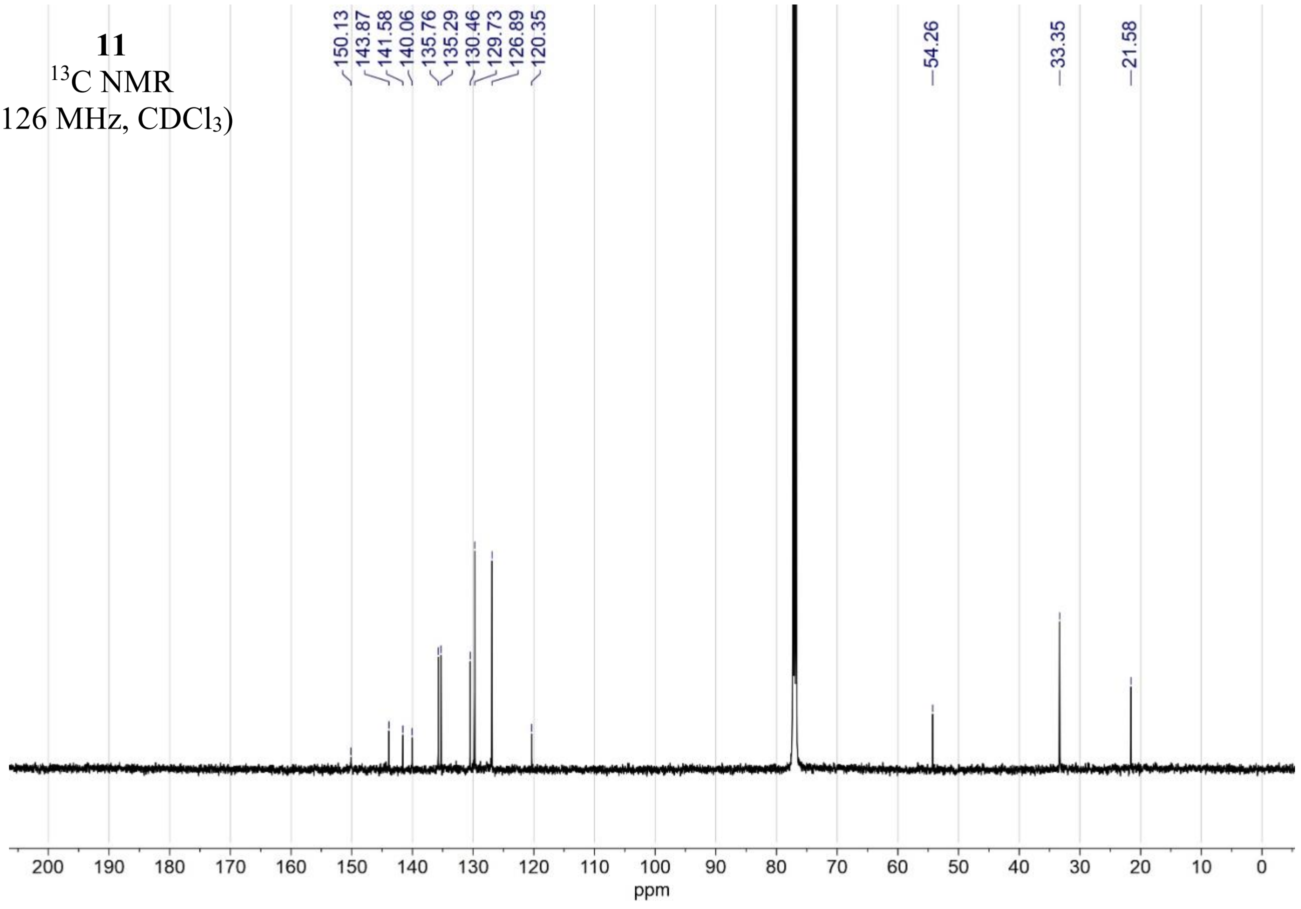

2-(2-Tosyl-4-(trifluoromethyl)phenyl)propan-2-amine 12

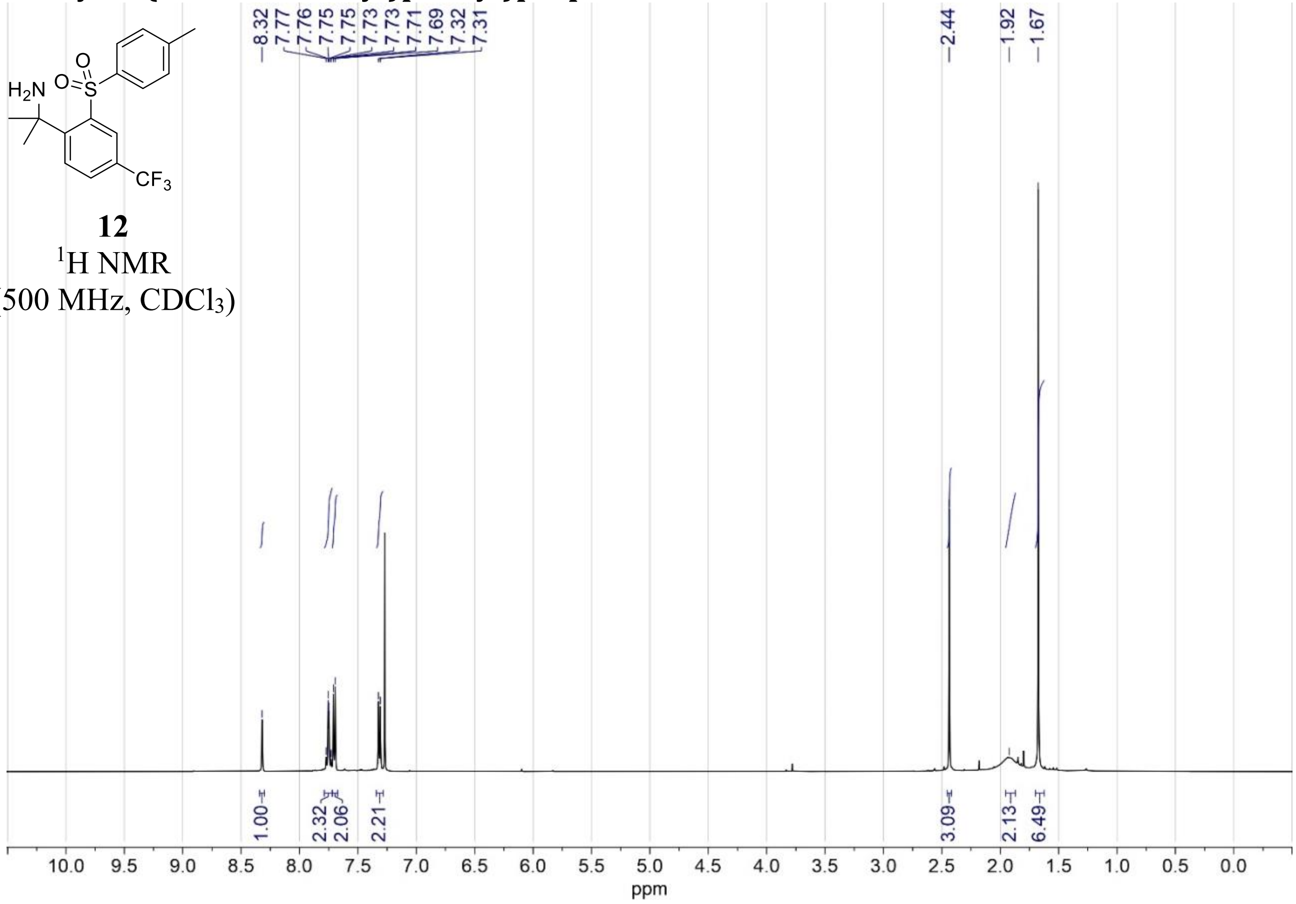

12
^{1}H NMR
(500 MHz, $CDCl_3$)

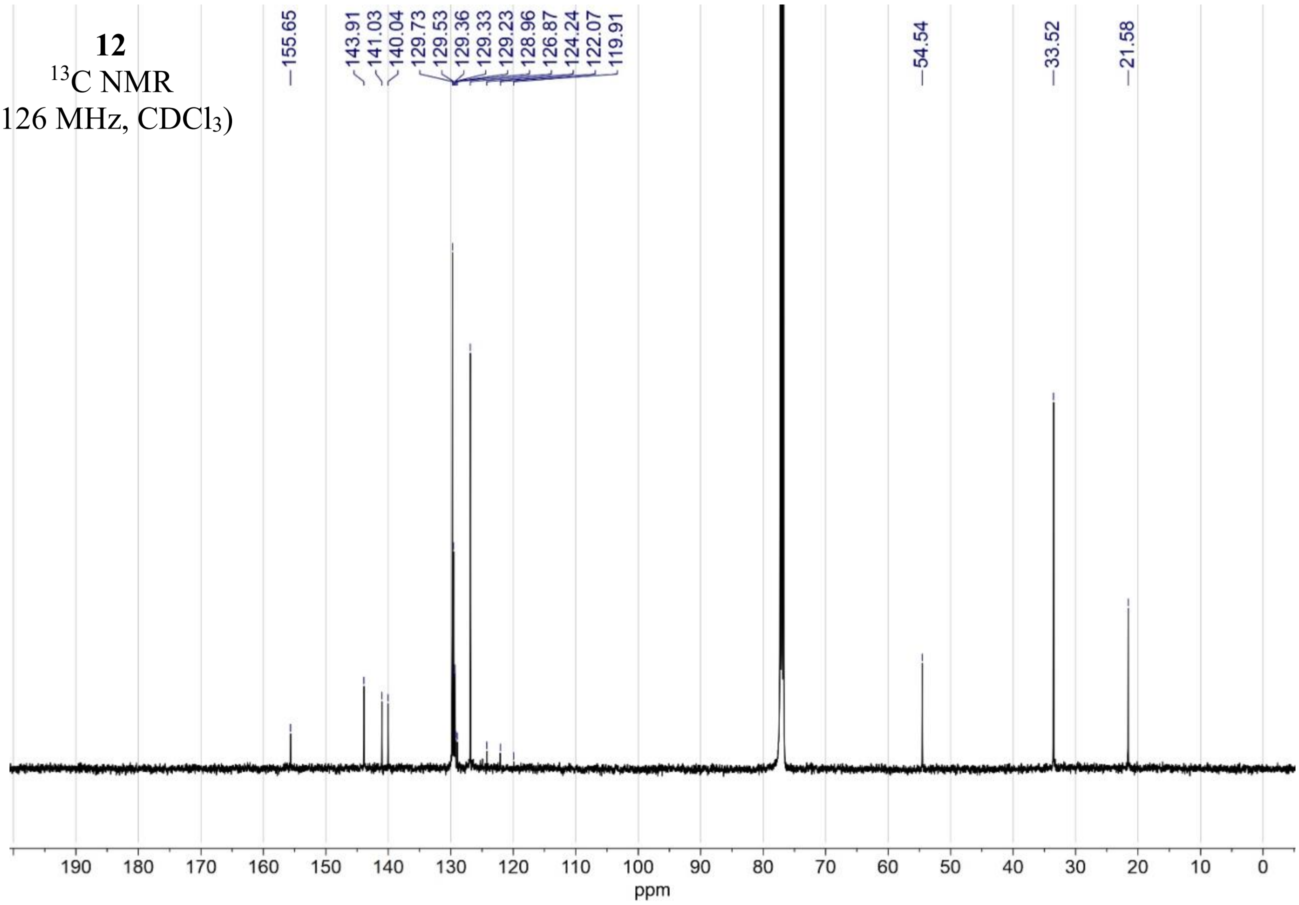

12
^{13}C NMR
(126 MHz, $CDCl_3$)

12
^{19}F NMR
(471 MHz, $CDCl_3$)

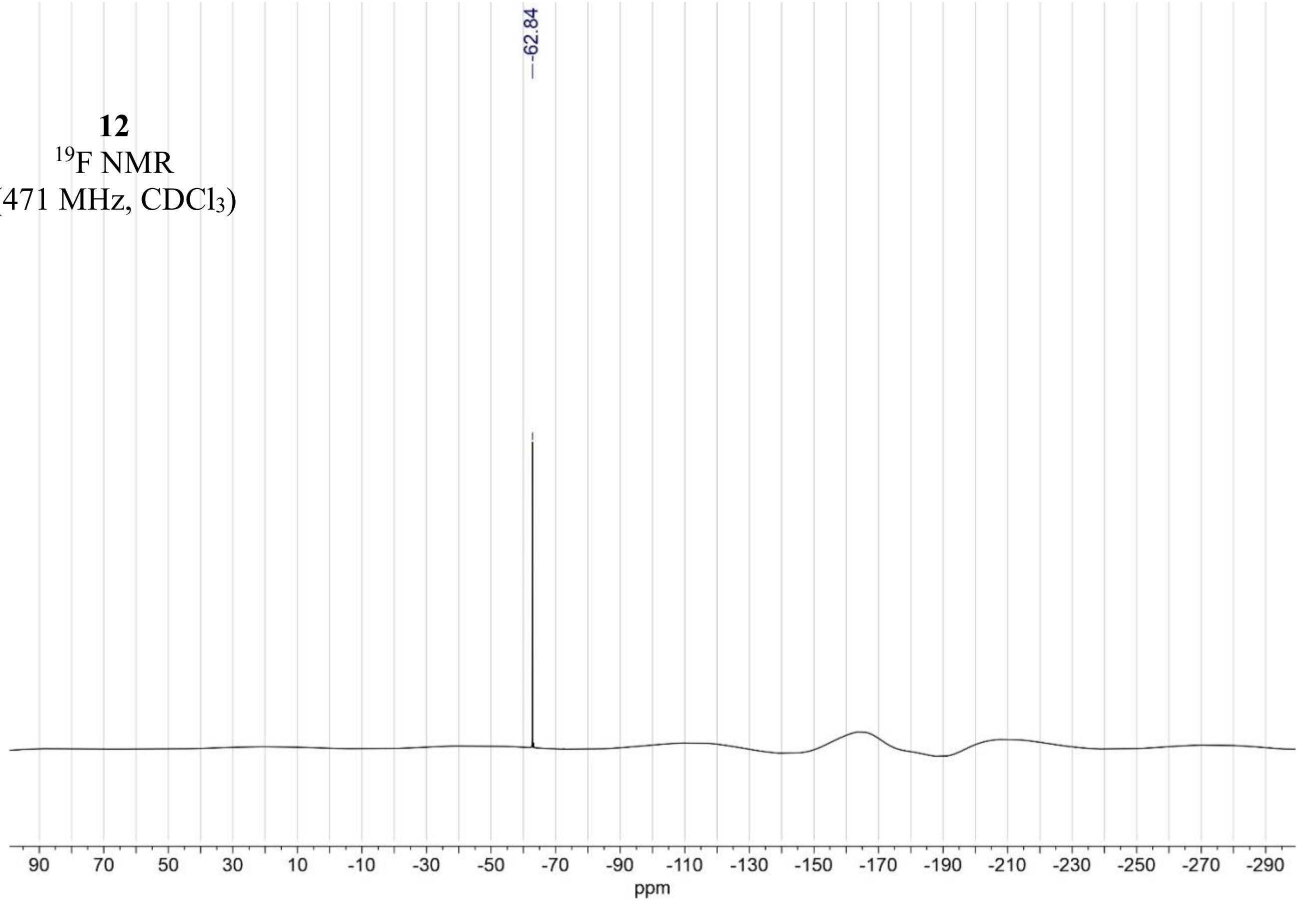

2-(3-Tosyl-[1,1'-biphenyl]-4-yl)propan-2-amine 13

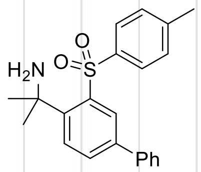

13
^{1}H NMR
(400 MHz, $CDCl_3$)

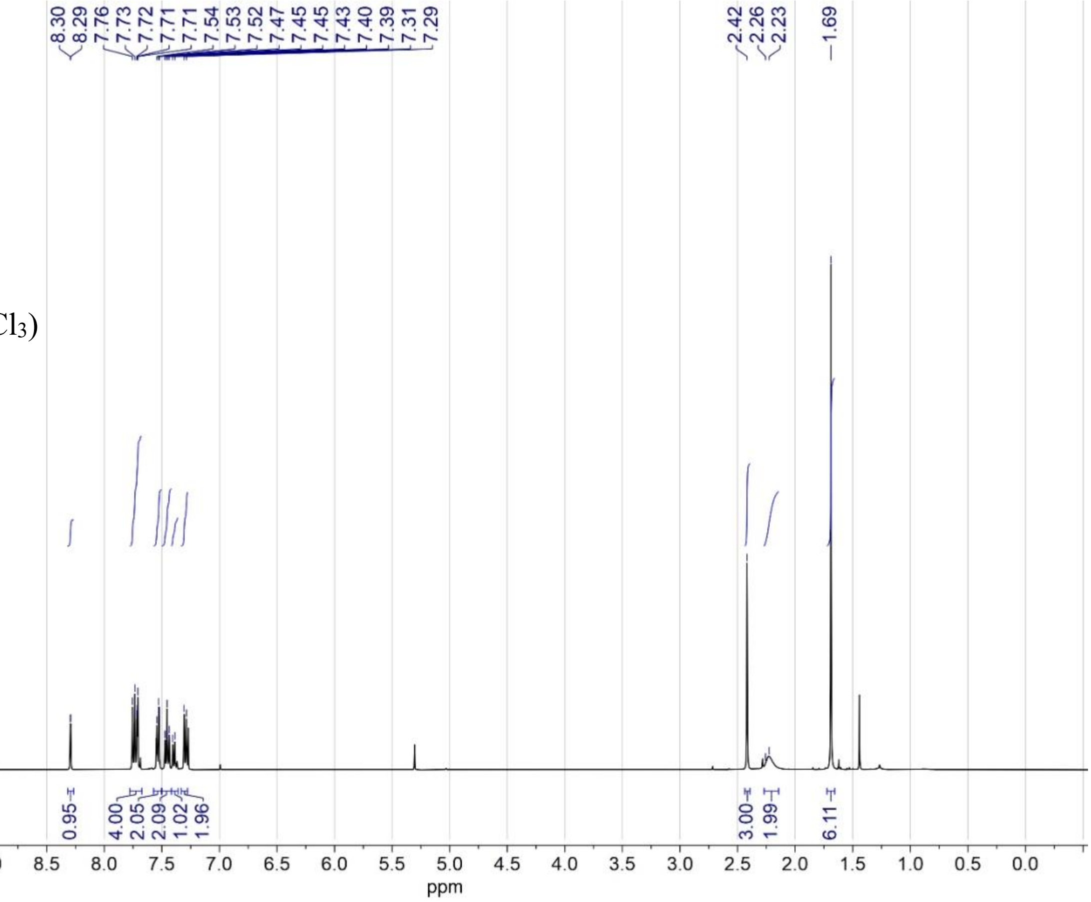

13
^{13}C NMR
(101 MHz, $CDCl_3$)

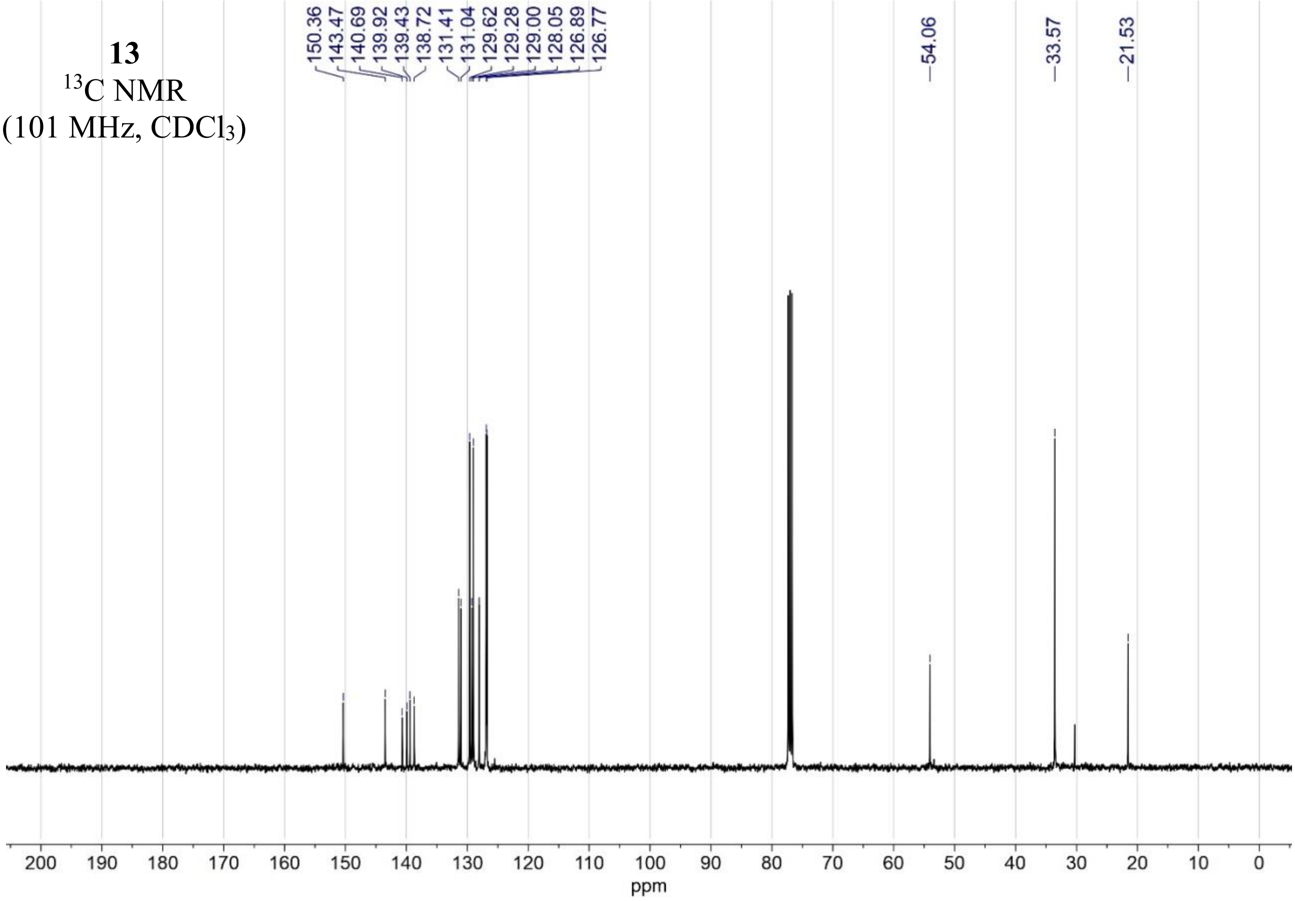

2-(2-Tosylphenyl)propan-2-amine 3

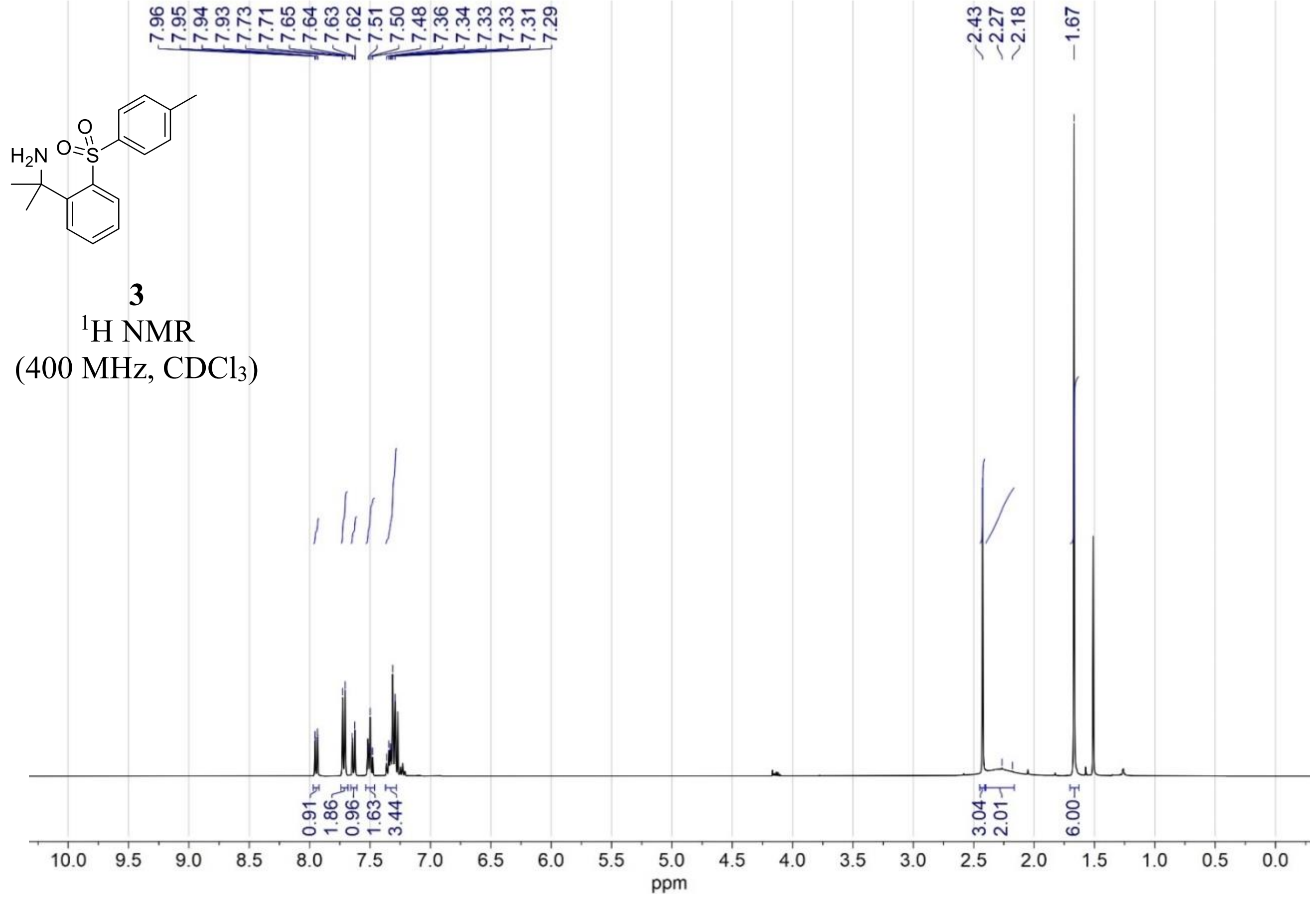

3
^{1}H NMR
(400 MHz, $CDCl_3$)

3
^{13}C NMR
(101 MHz, $CDCl_3$)

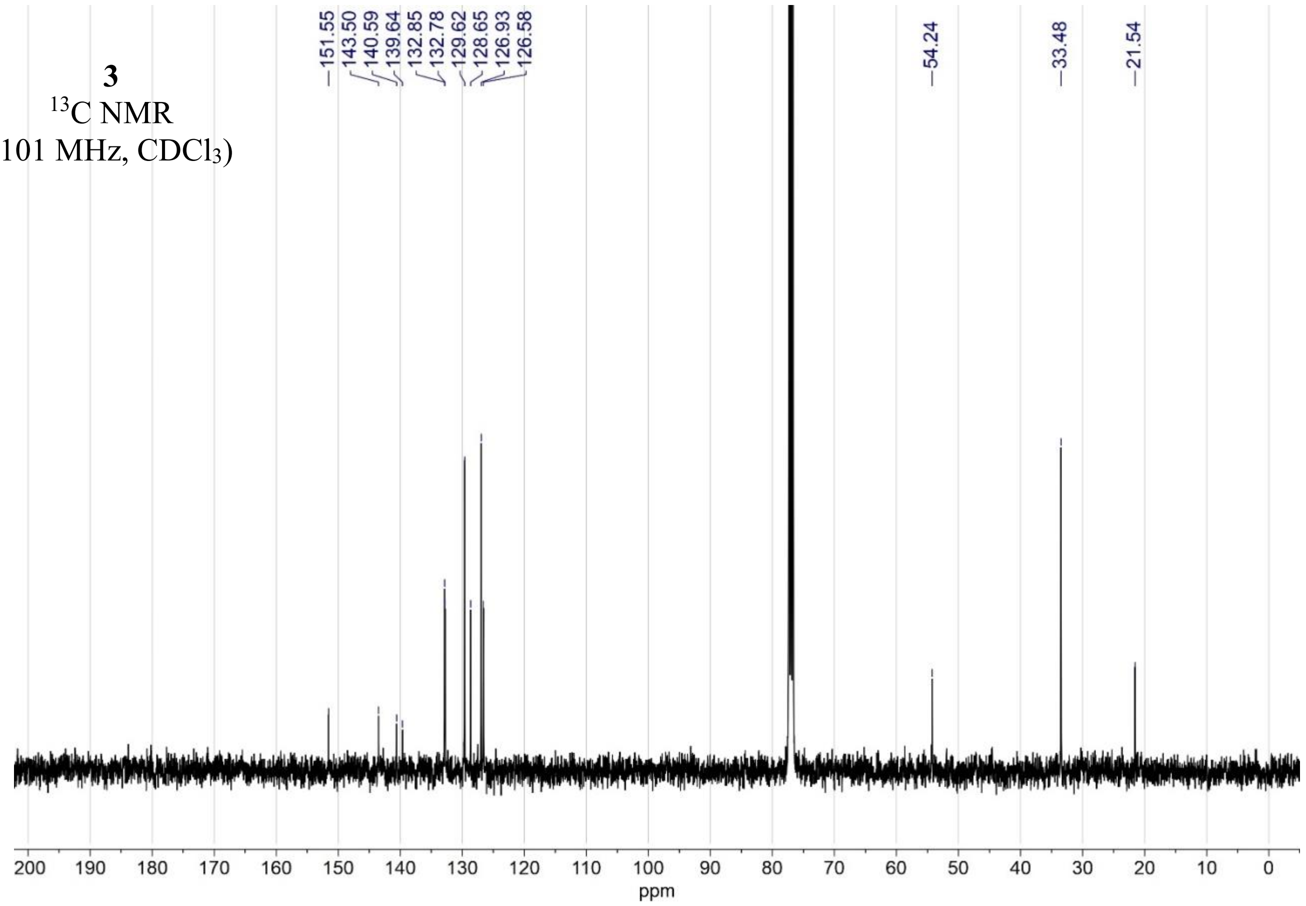

2-(2-(*m*-Tolylsulfonyl)phenyl)propan-2-amine 14

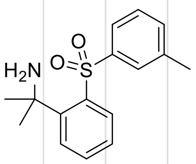

14
^{1}H NMR
(400 MHz, $CDCl_3$)

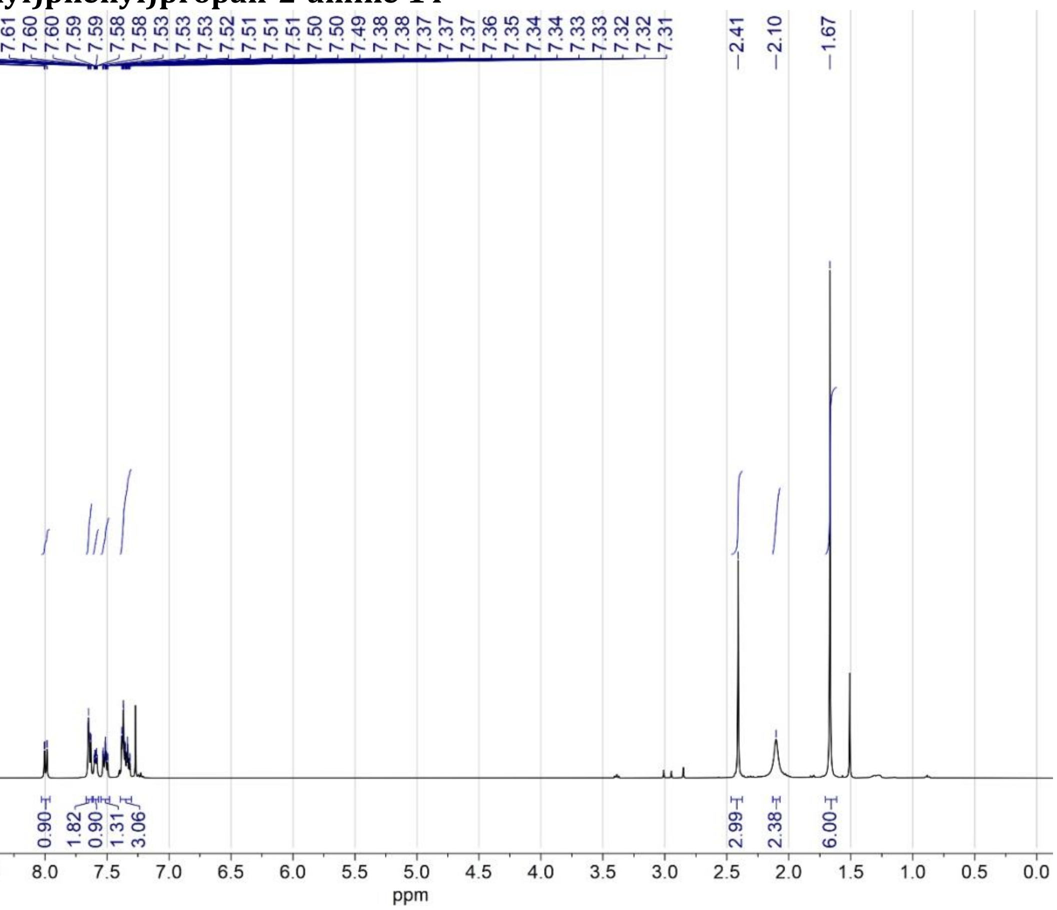

14
^{13}C NMR
(101 MHz, $CDCl_3$)

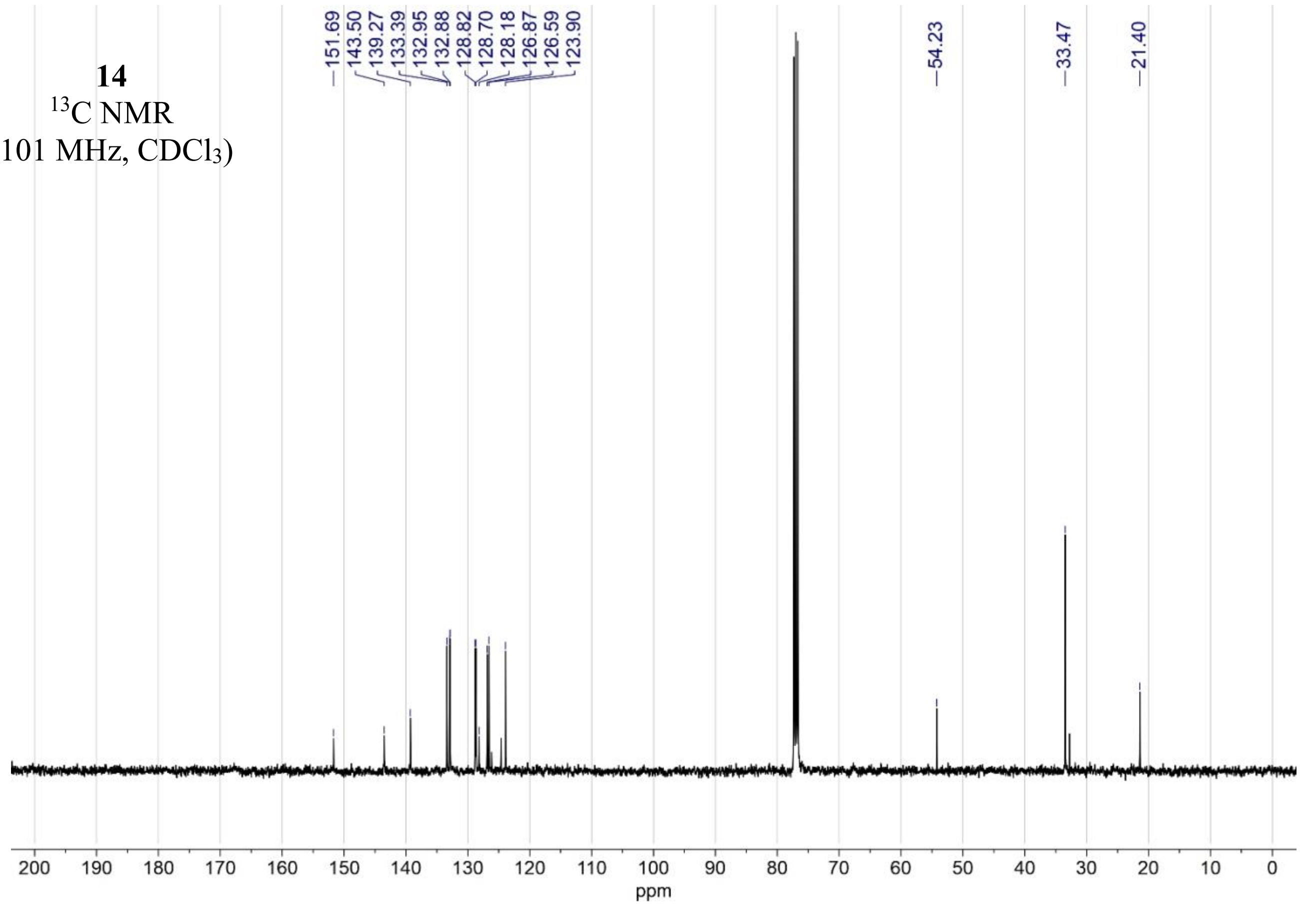

2-(2-(o-Tolylsulfonyl)phenyl)propan-2-amine 15

15
^{1}H NMR
(400 MHz, $CDCl_3$)

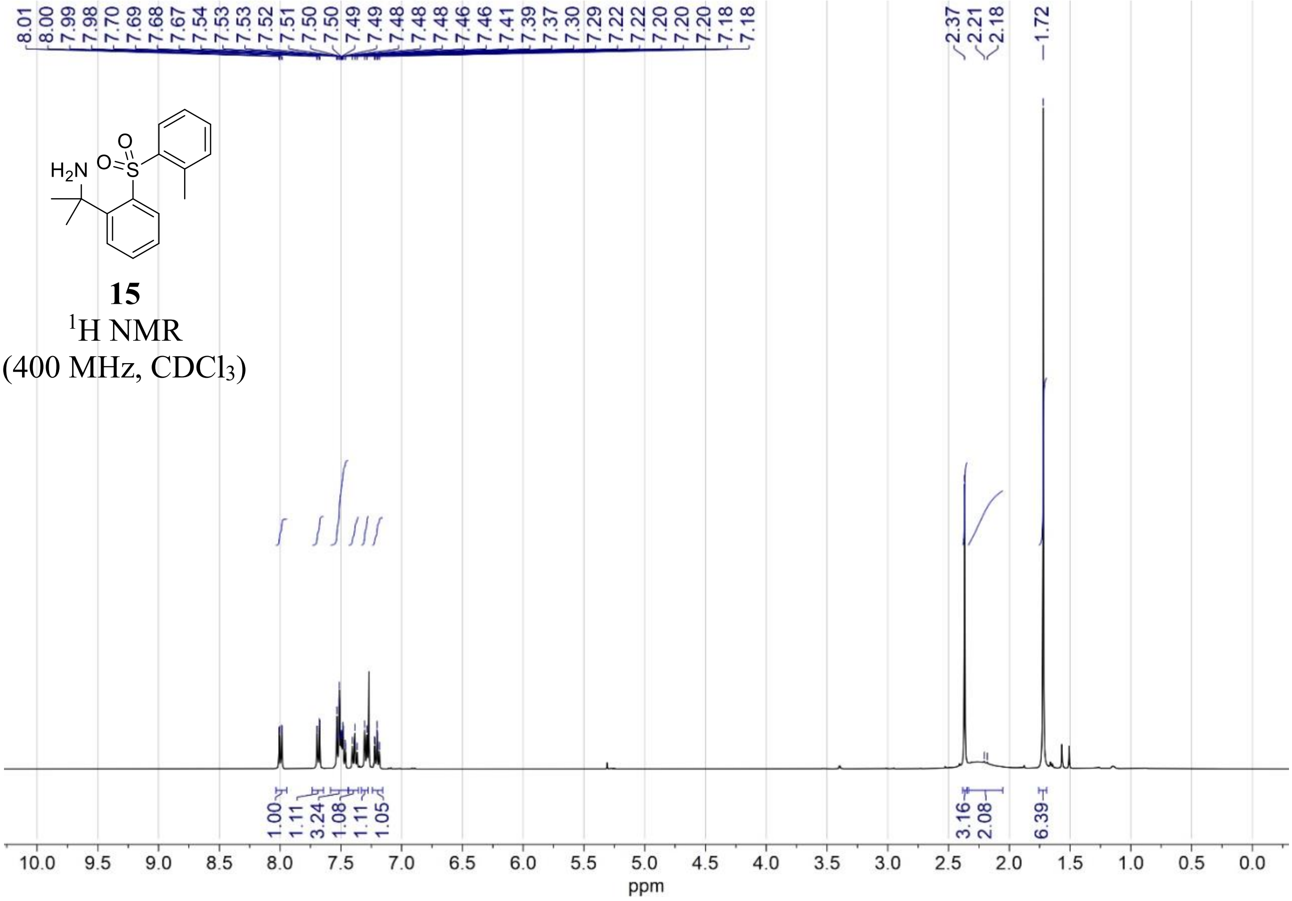

15
^{13}C NMR
(101 MHz, $CDCl_3$)

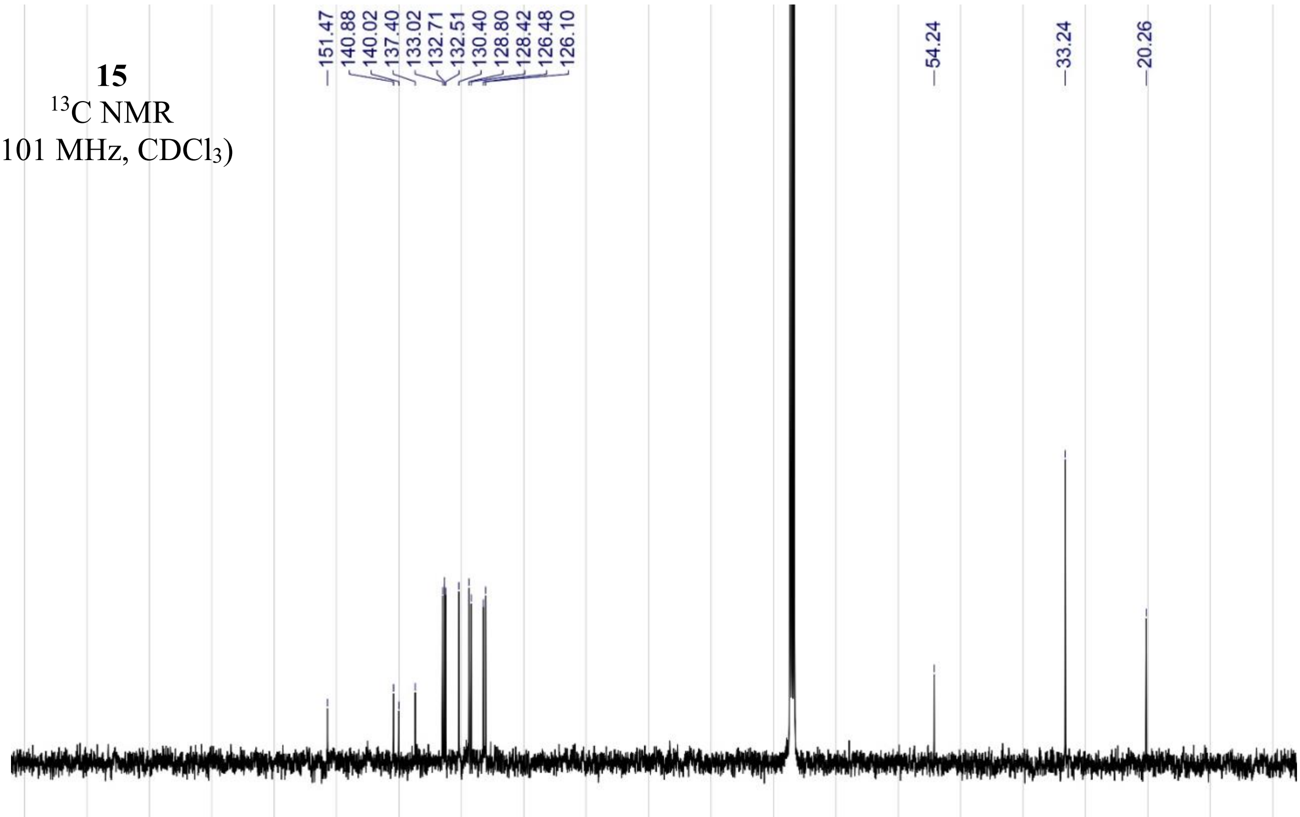

2-(2-(Methylsulfonyl)phenyl)propan-2-amine 16

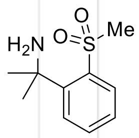

16
^{1}H NMR
(400 MHz, $CDCl_3$)

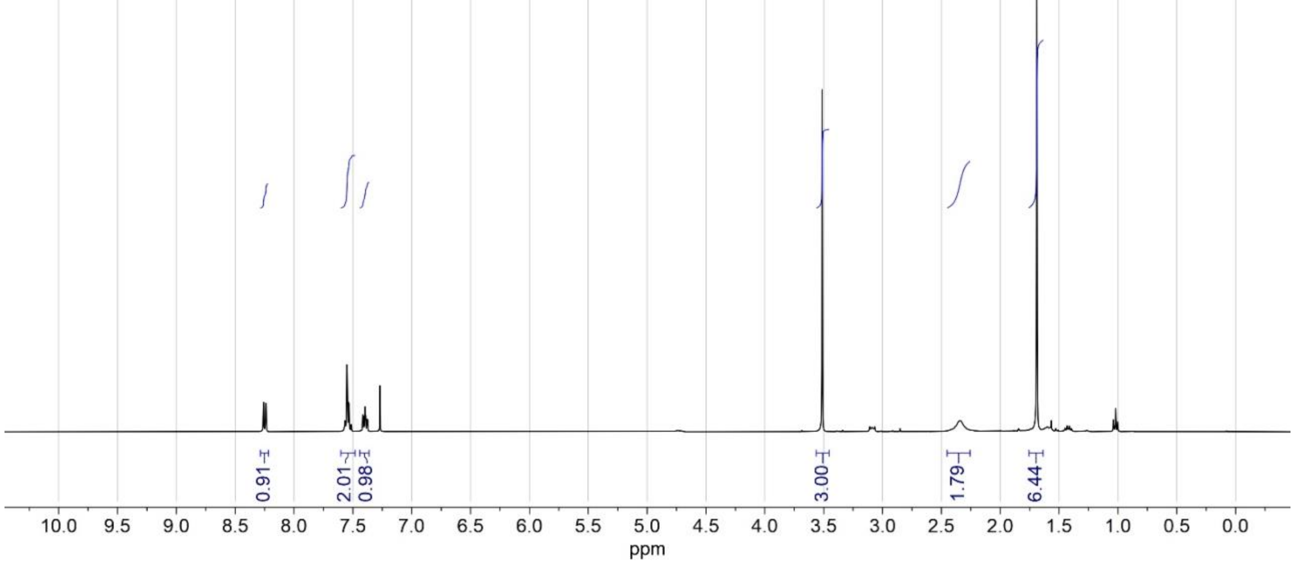

16
^{13}C NMR
(101 MHz, $CDCl_3$)

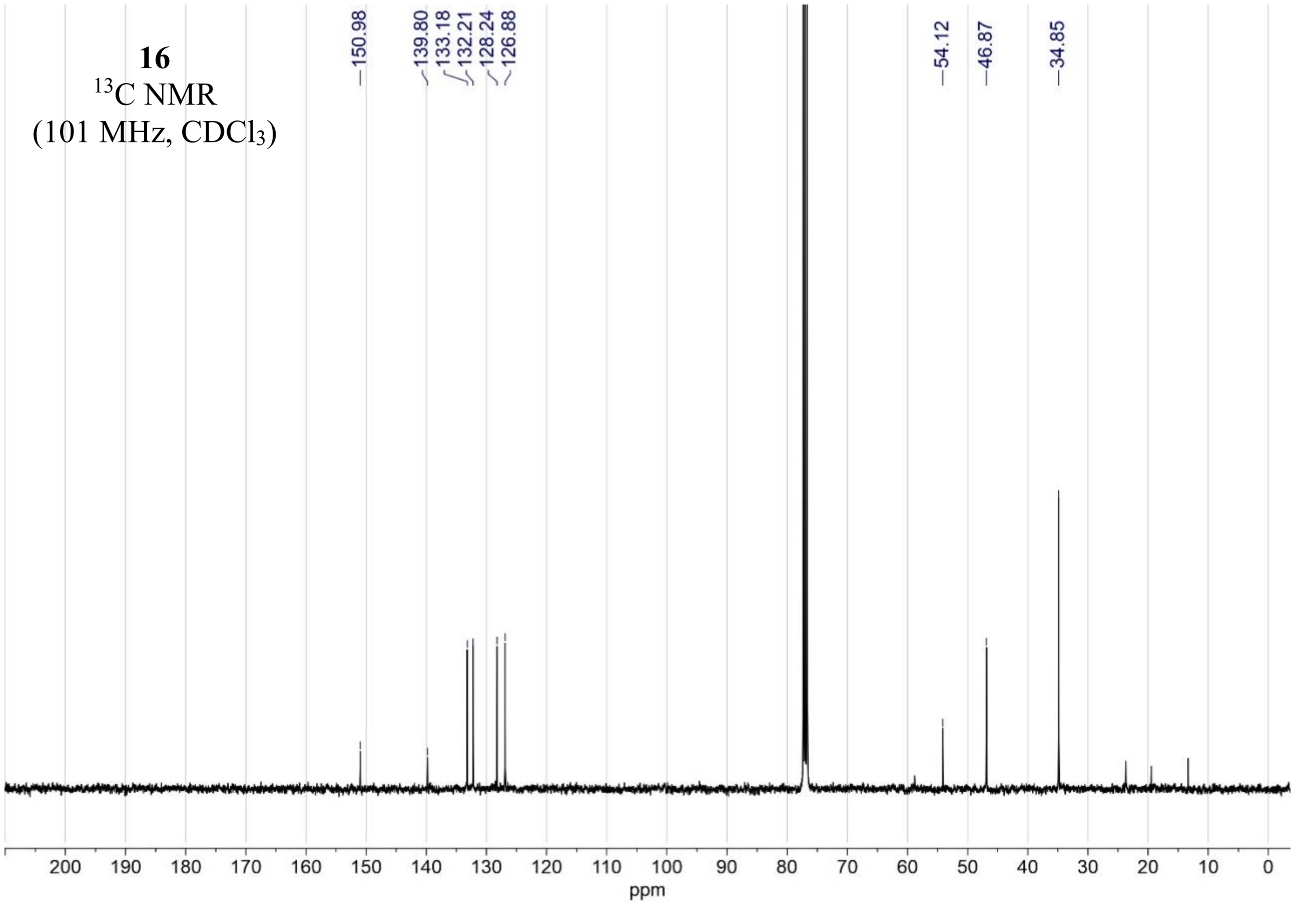

2-(2-(Cyclopropylsulfonyl)phenyl)propan-2-amine 17

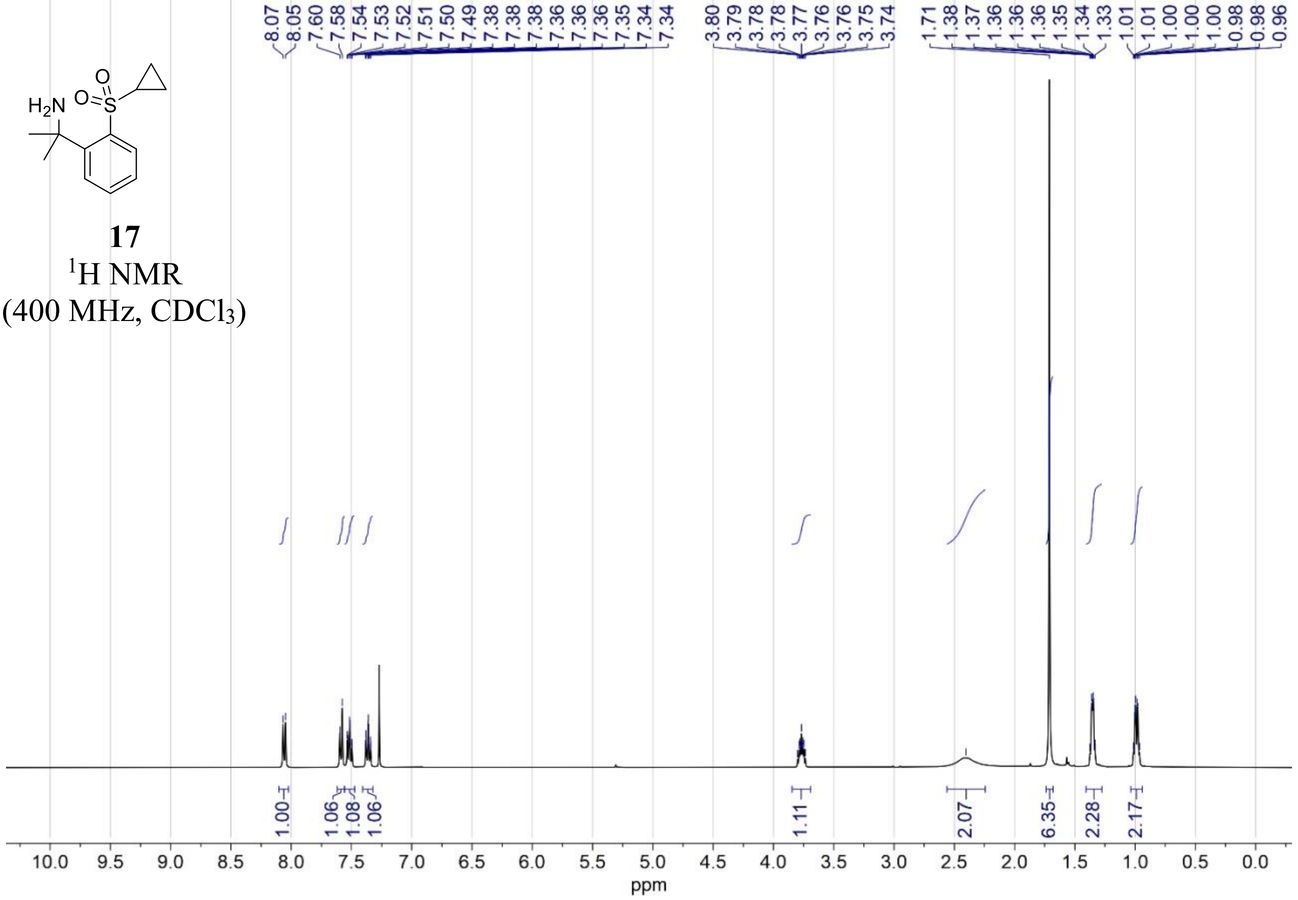

17
^{1}H NMR
(400 MHz, $CDCl_3$)

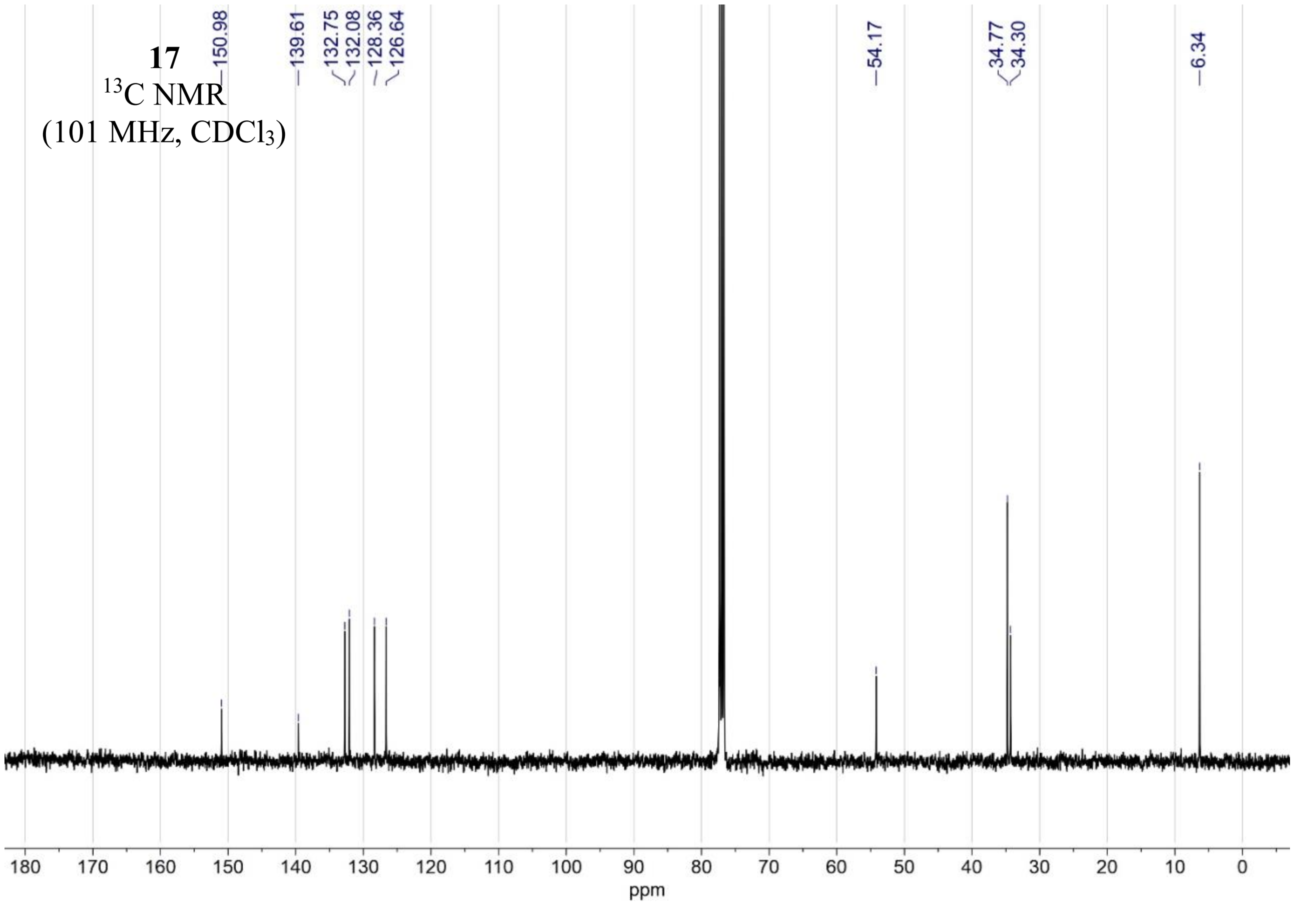

17
^{13}C NMR
(101 MHz, $CDCl_3$)

2-(2-(Bicyclo[1.1.1]pentan-1-ylsulfonyl)phenyl)propan-2-amine 18

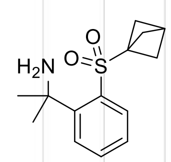

18
^{1}H NMR
(400 MHz, $CDCl_3$)

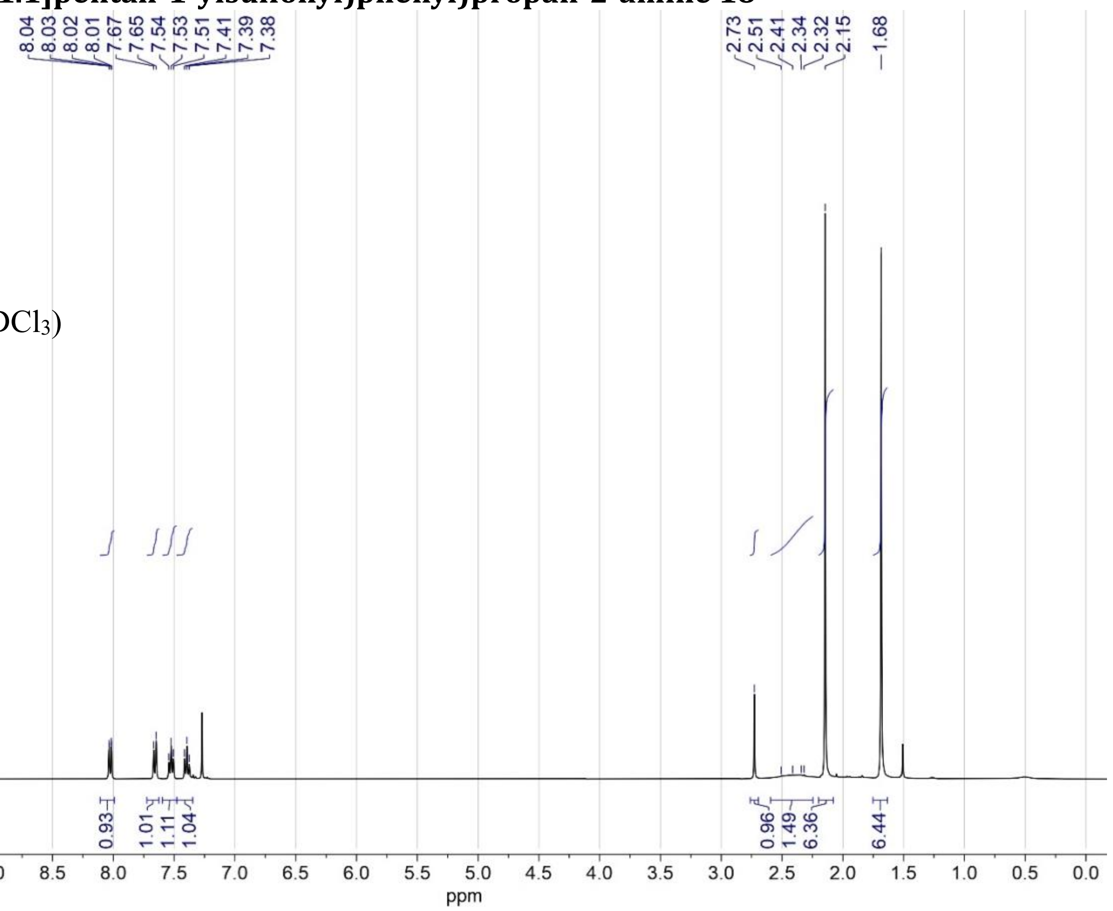

18
^{13}C NMR
(101 MHz, $CDCl_3$)

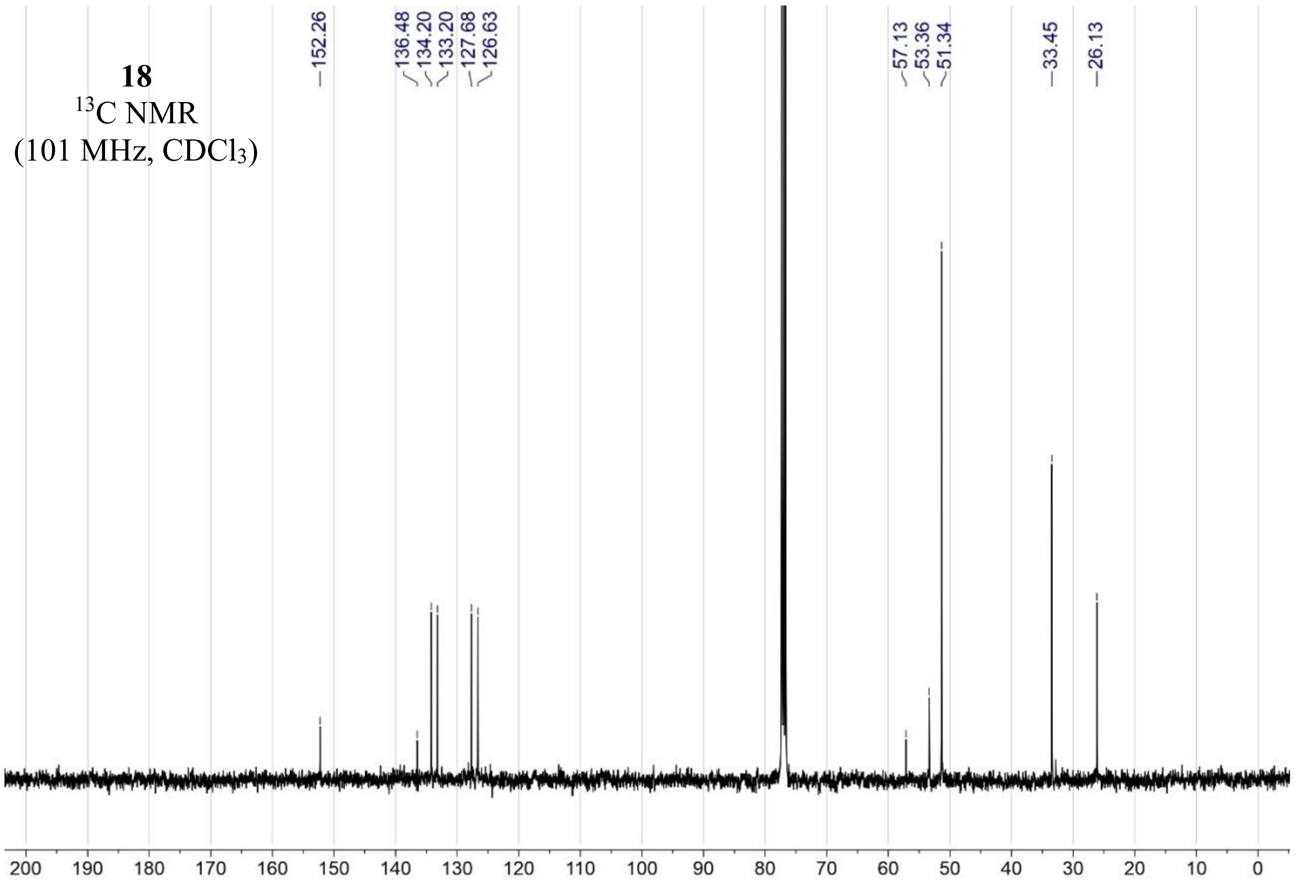

2-(2-(4-Fluorobenzenesulfonyl)phenyl)propan-2-amine 19

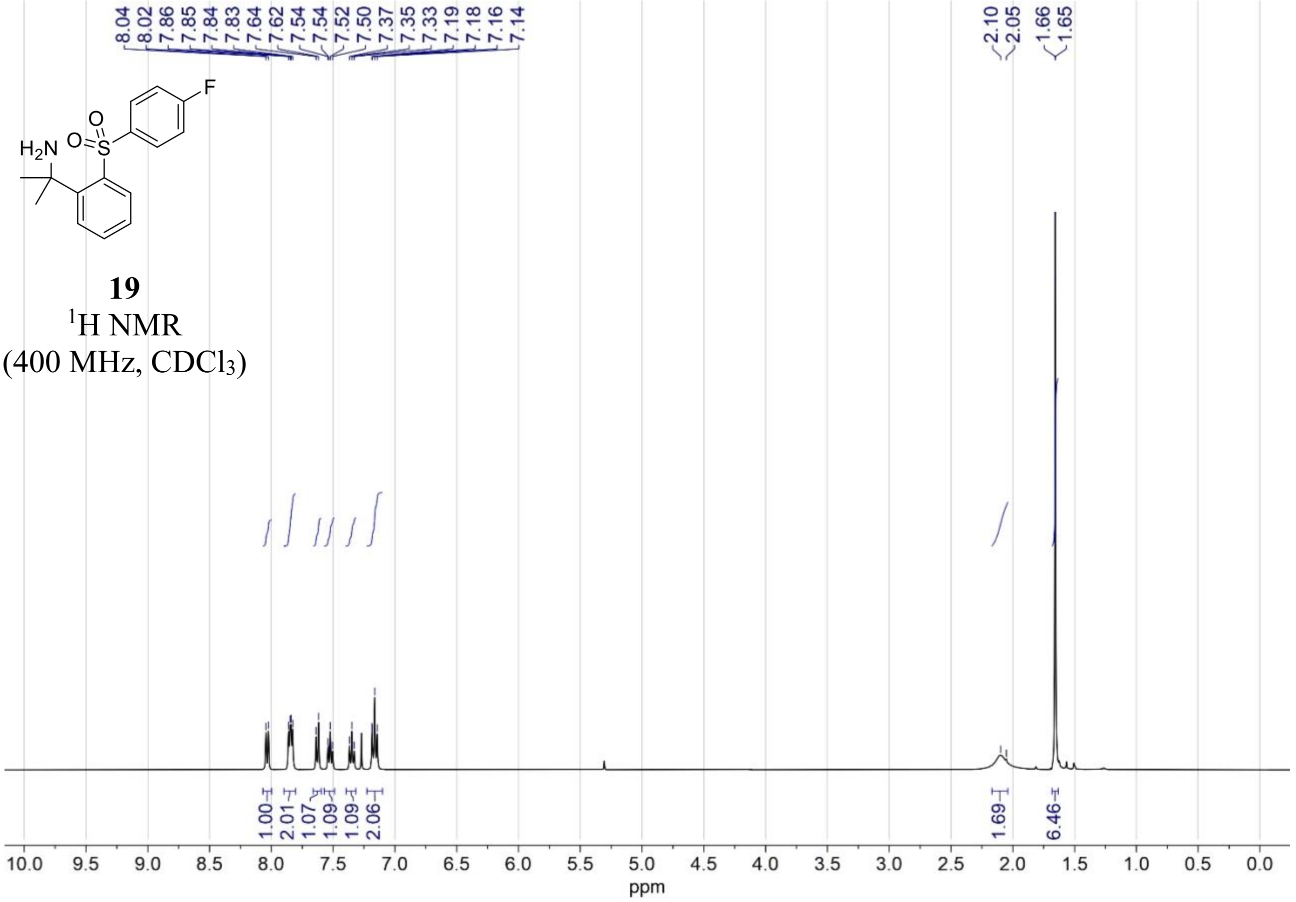

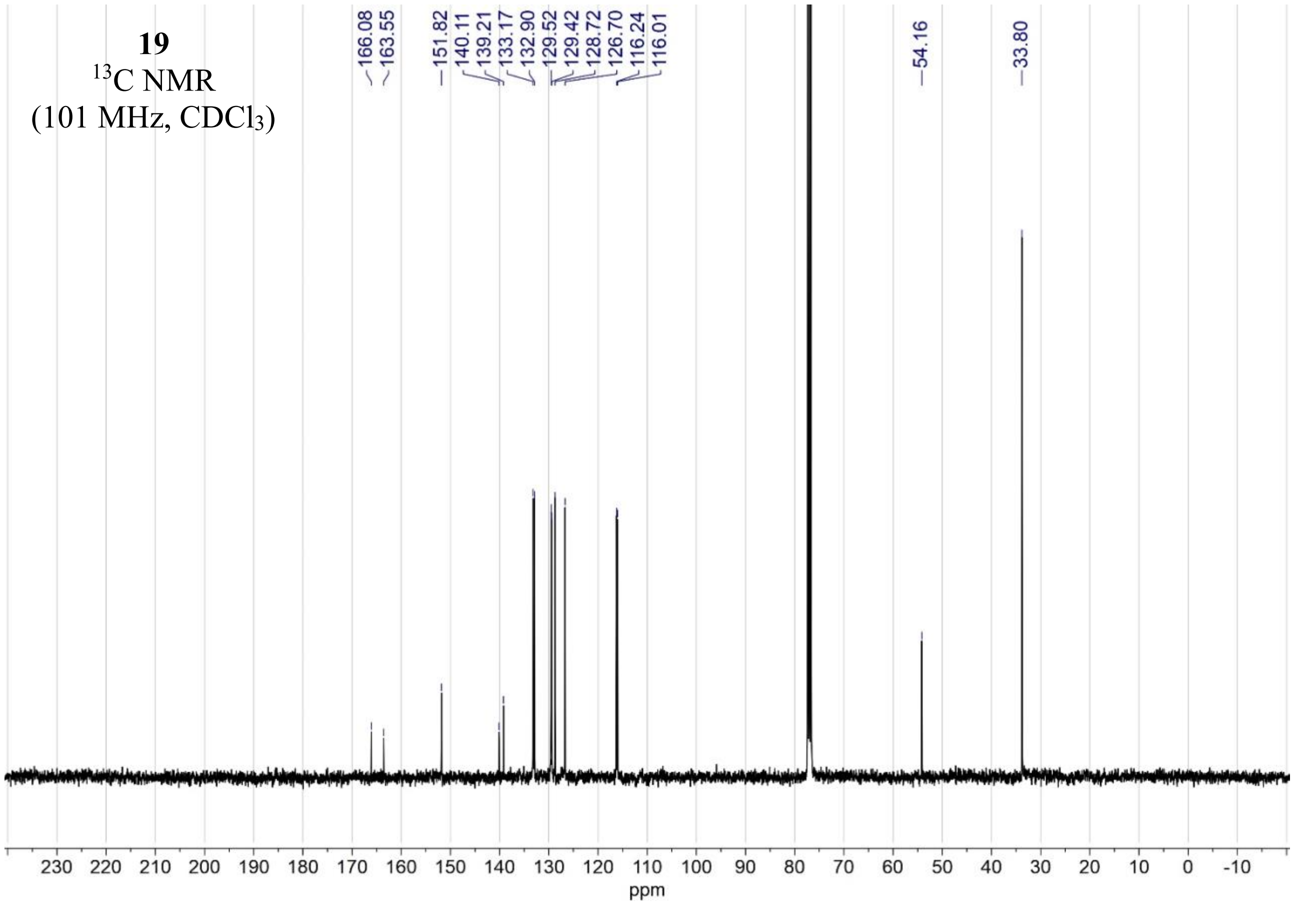

19
^{19}F NMR
(377 MHz, $CDCl_3$)

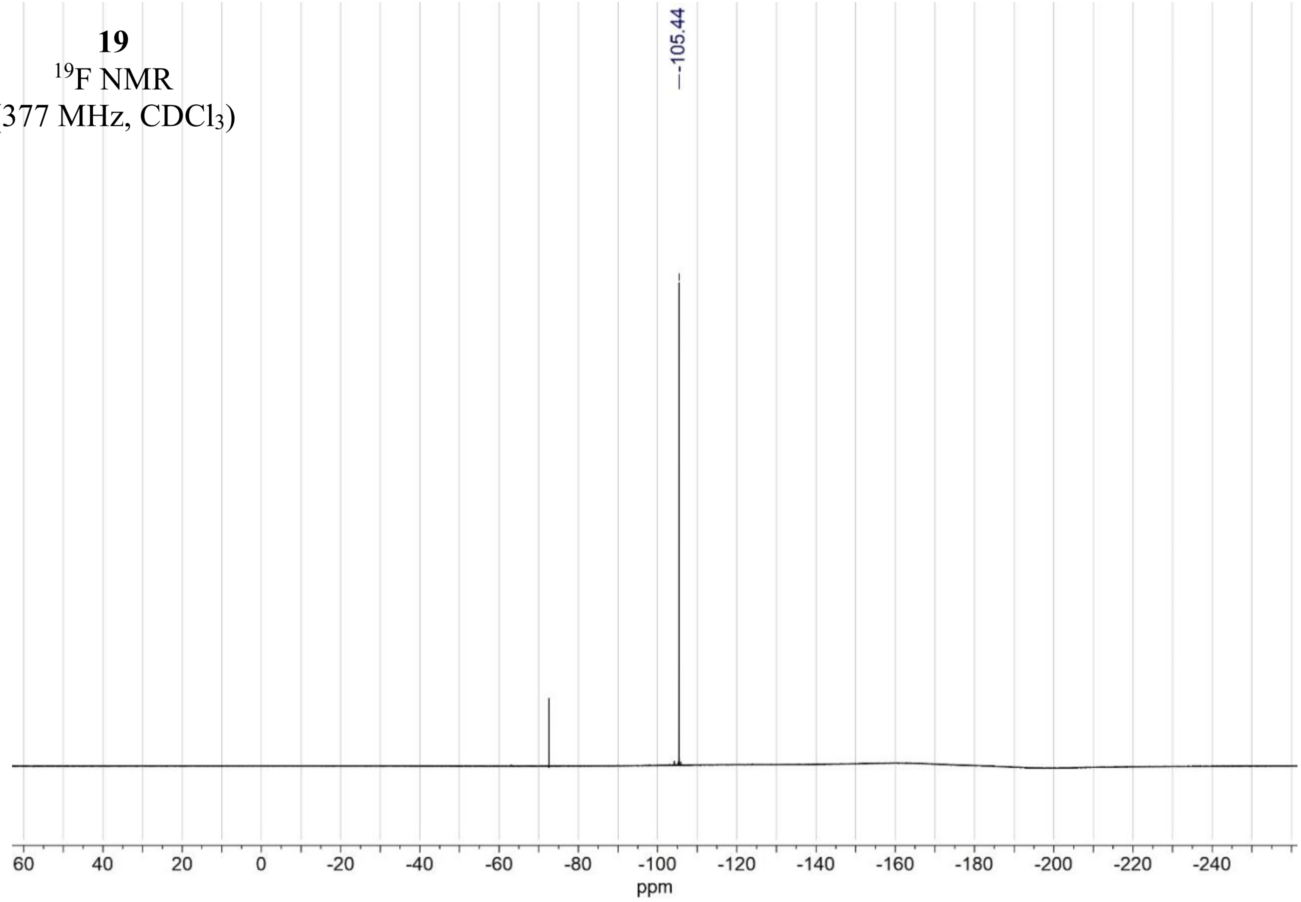

2-(2-(4-Chlorobenzenesulfonyl)phenyl)propan-2-amine 20

20
^{1}H NMR
(400 MHz, $CDCl_3$)

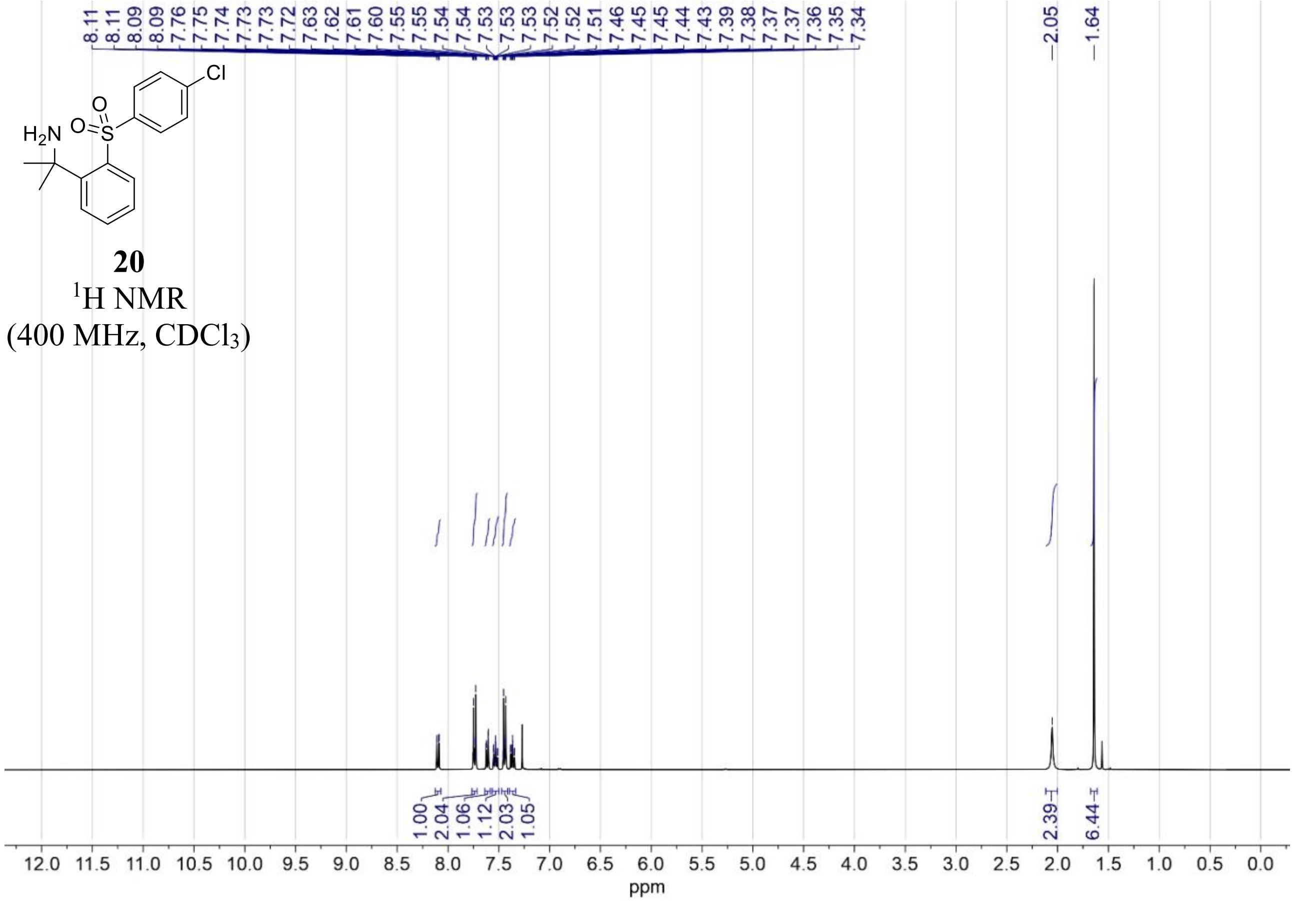

20
^{13}C NMR
(101 MHz, $CDCl_3$)

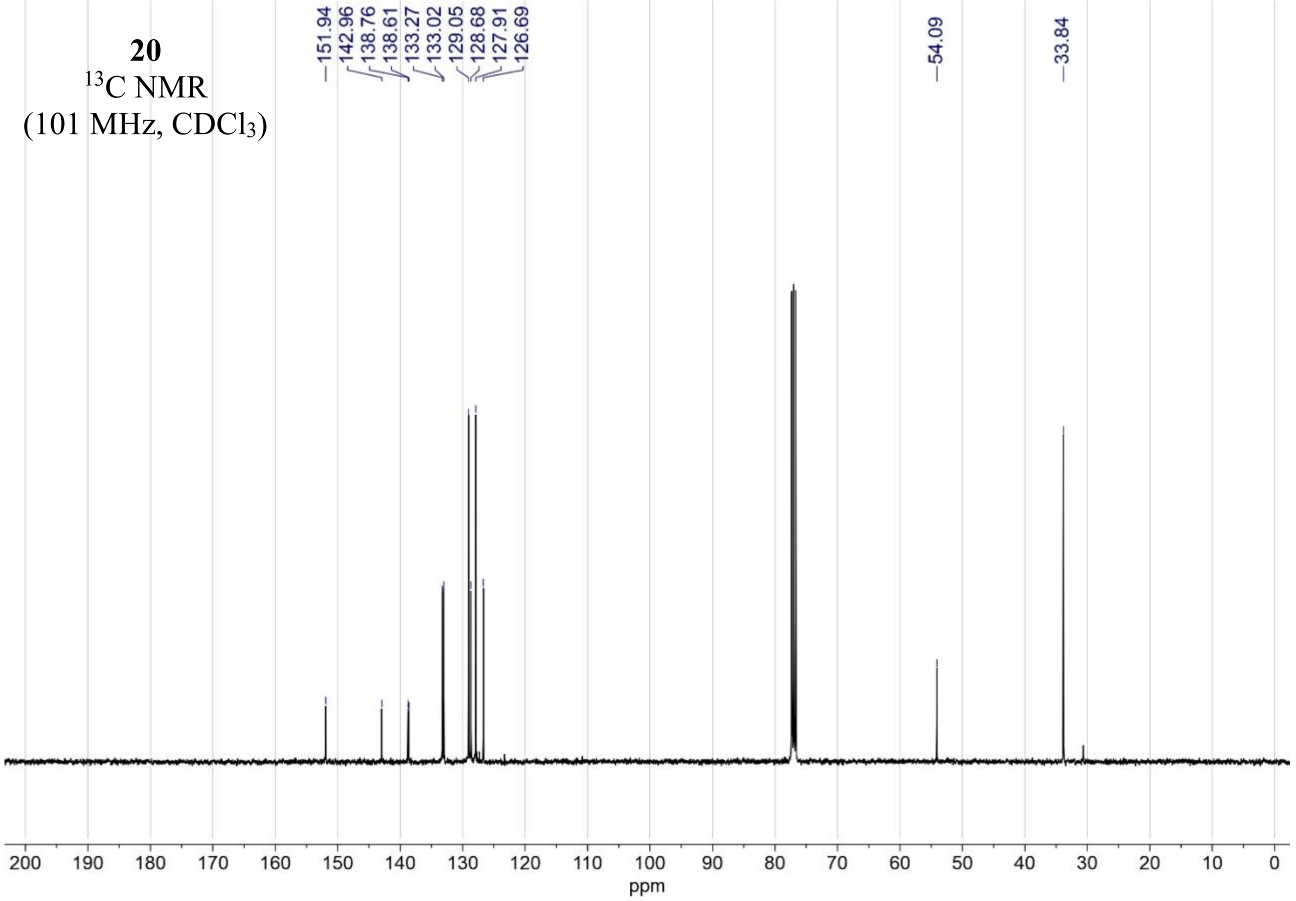

2-(2-(4-Bromobenzenesulfonyl)phenyl)propan-2-amine 21

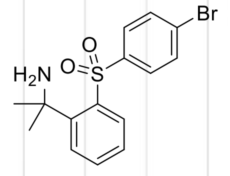

21
^{1}H NMR
(400 MHz, $CDCl_3$)

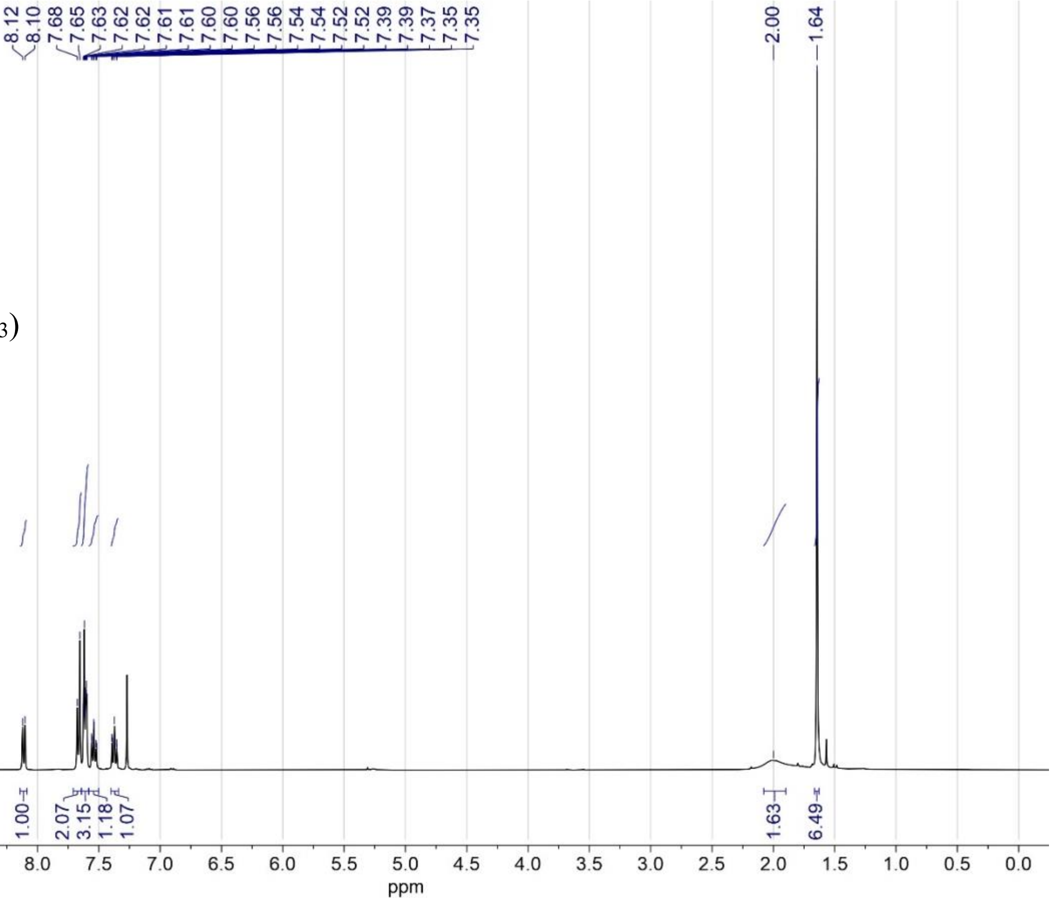

21
^{13}C NMR
(101 MHz, $CDCl_3$)

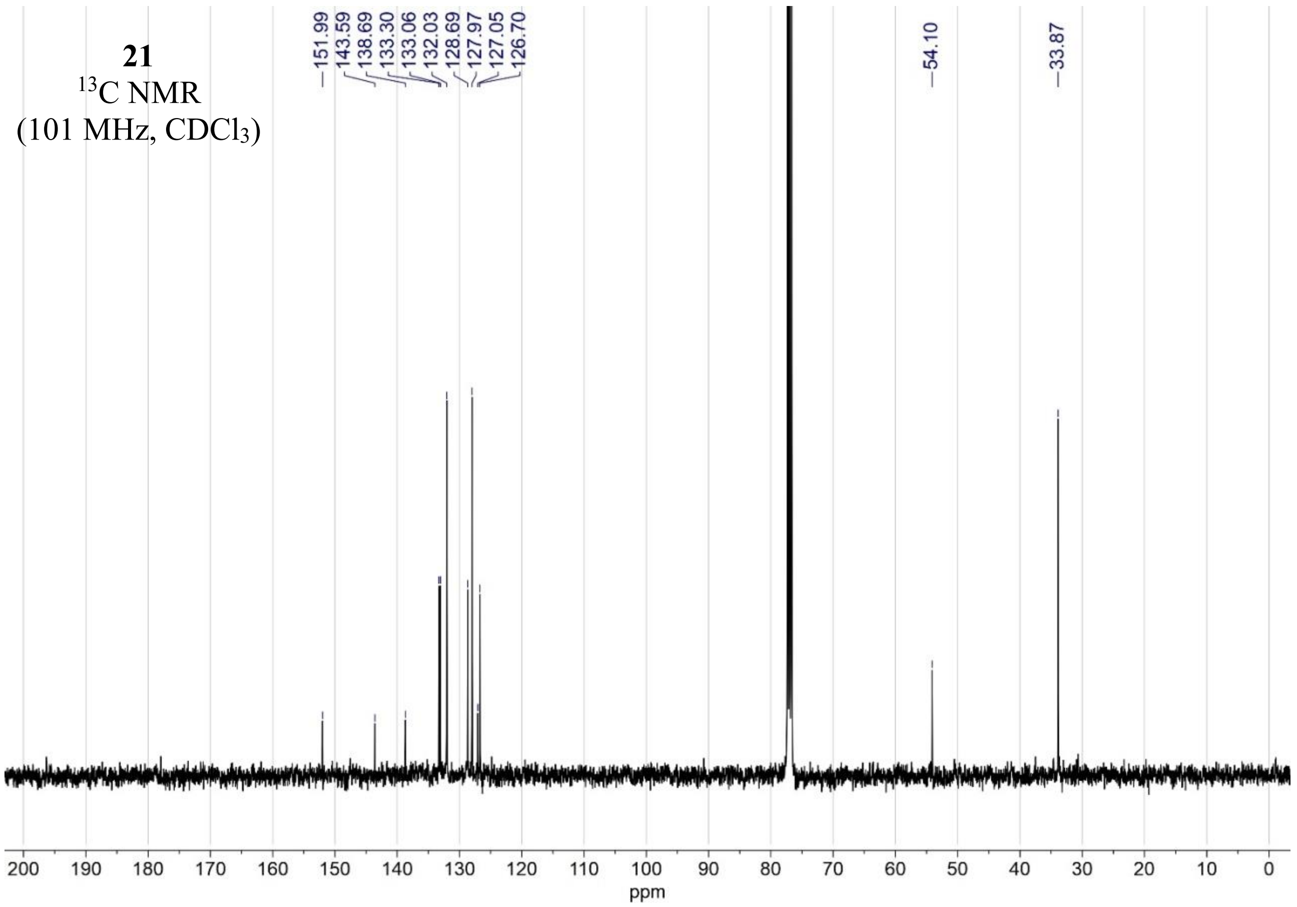

2-(2-(Naphthalen-2-ylsulfonyl)phenyl)propan-2-amine 22

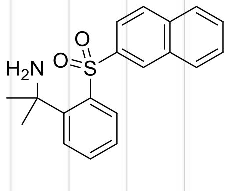

22
^{1}H NMR
(500 MHz, $CDCl_3$)

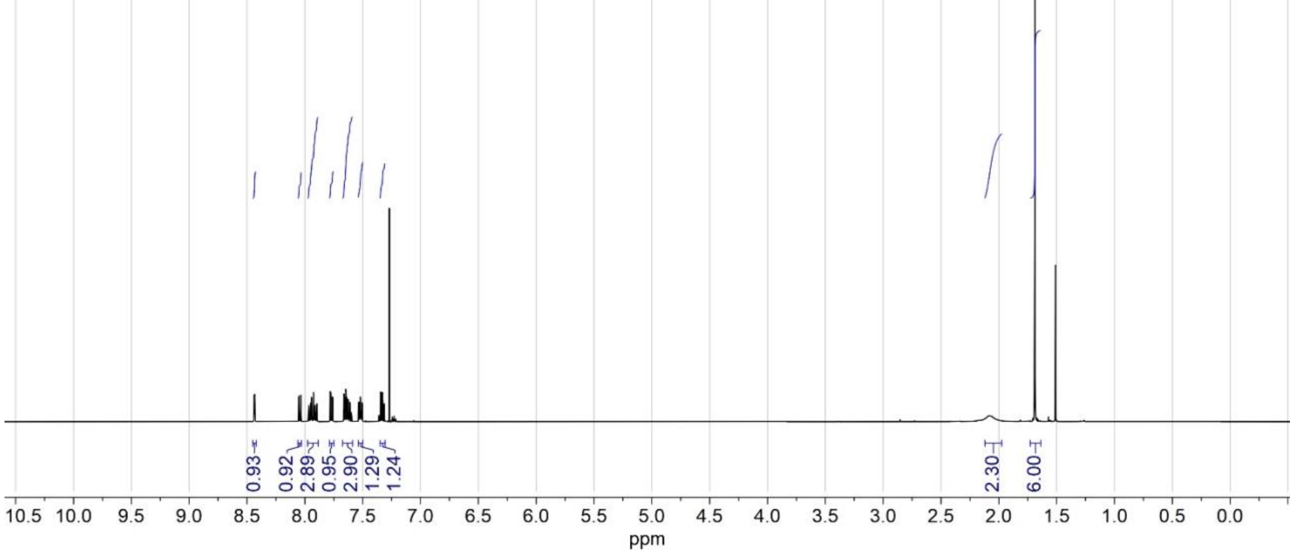

22
^{13}C NMR
(126 MHz, $CDCl_3$)

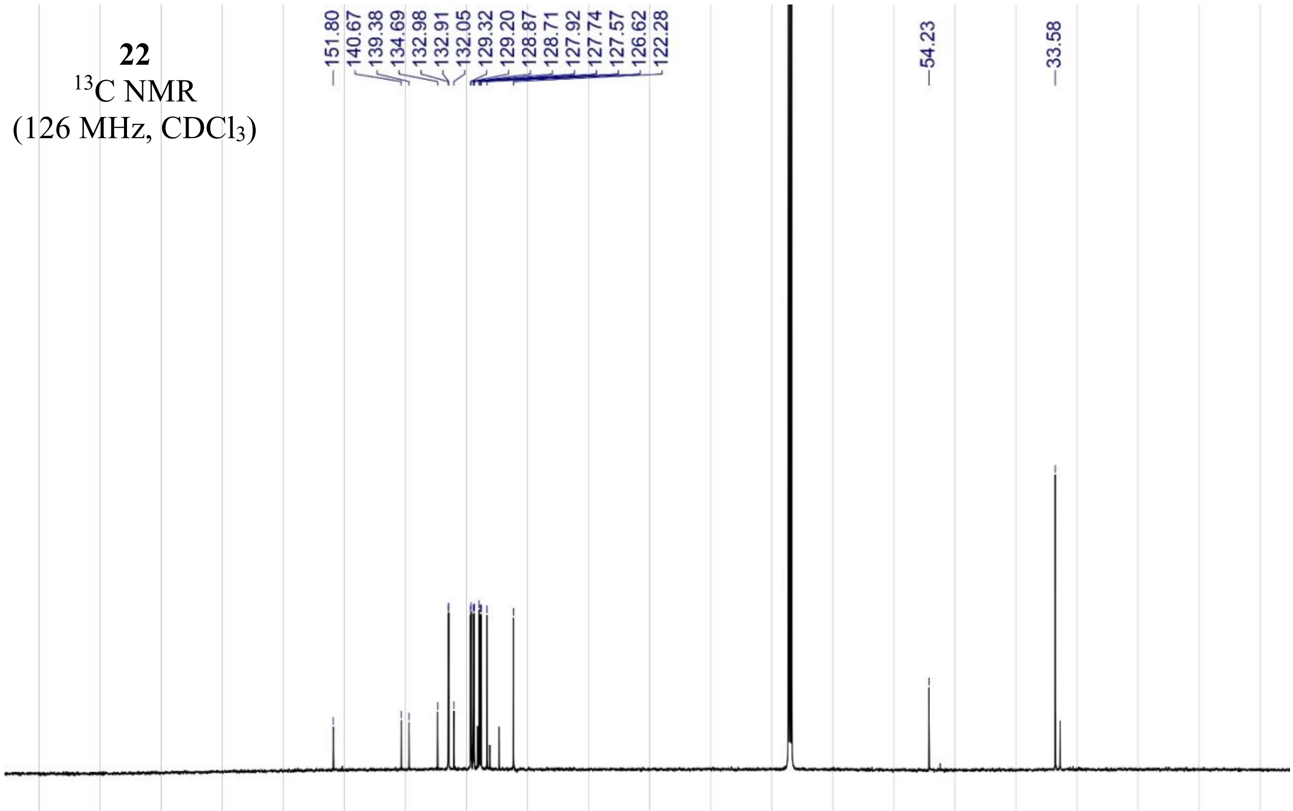

2-(2-(Phenylsulfonyl)phenyl)propan-2-amine 23

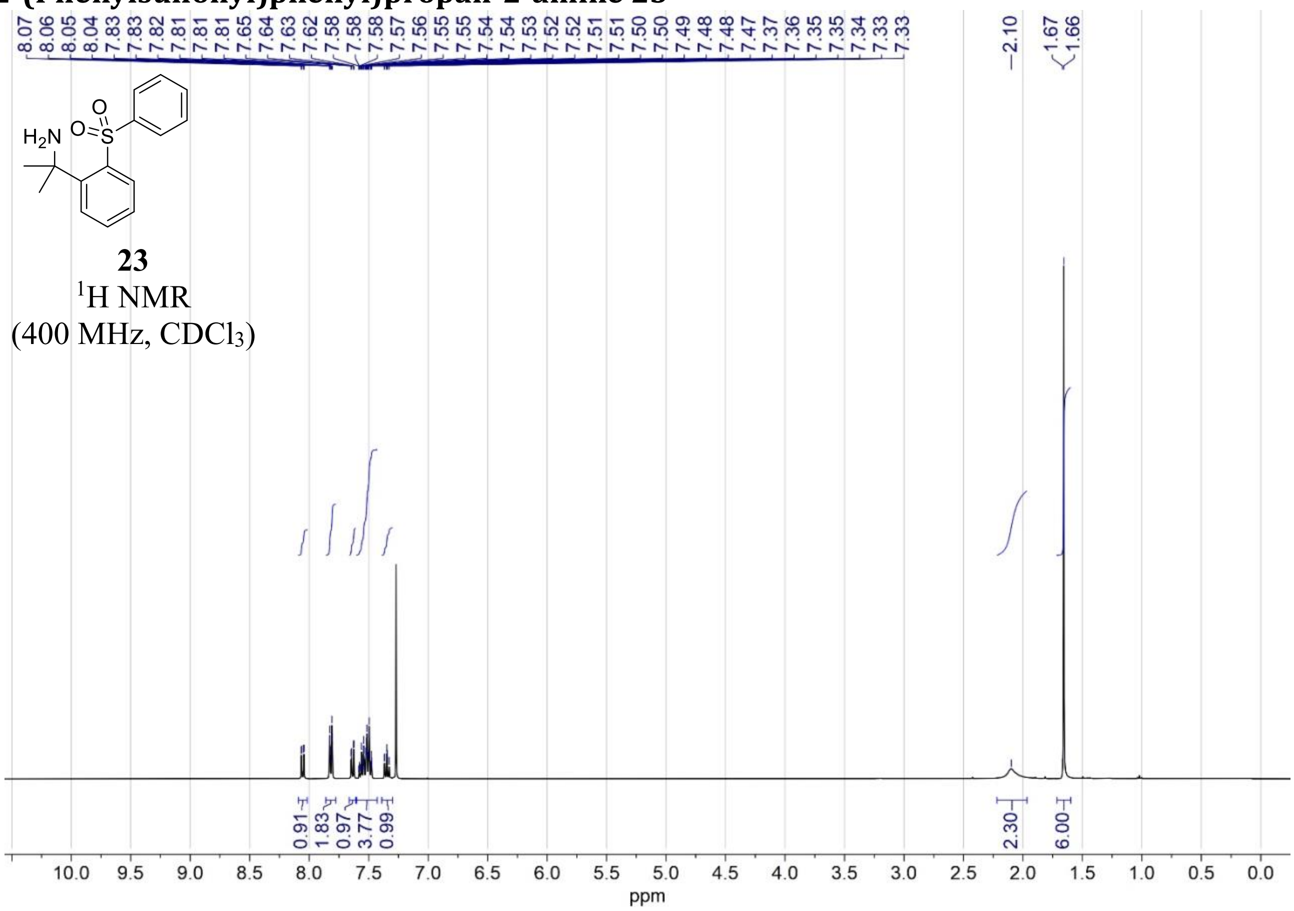

23
^{1}H NMR
(400 MHz, $CDCl_3$)

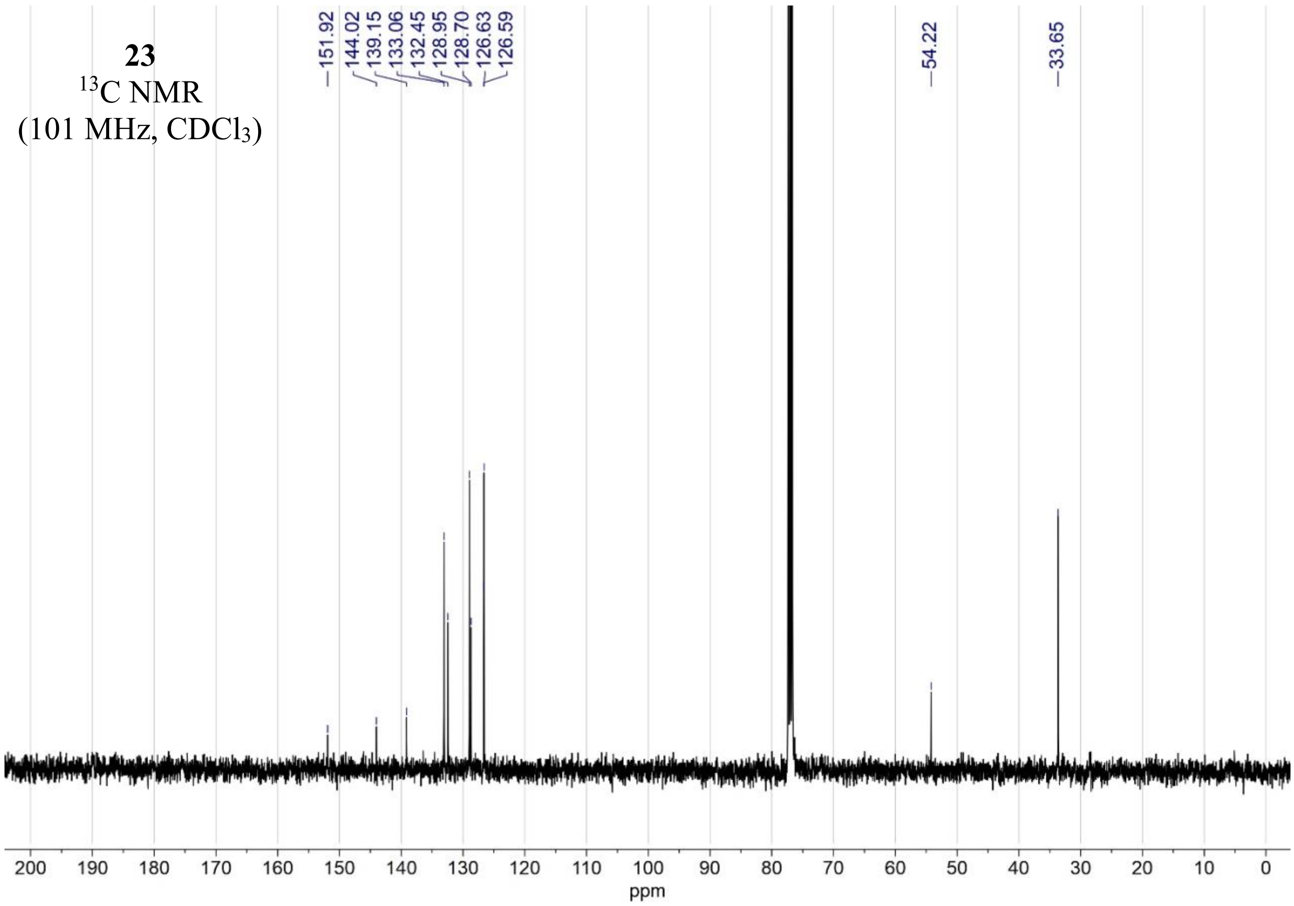

23
^{13}C NMR
(101 MHz, $CDCl_3$)

2-(2-((4-(*tert*-Butyl)phenyl)sulfonyl)phenyl)propan-2-amine 24

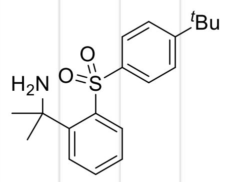

24
^{1}H NMR
(500 MHz, $CDCl_3$)

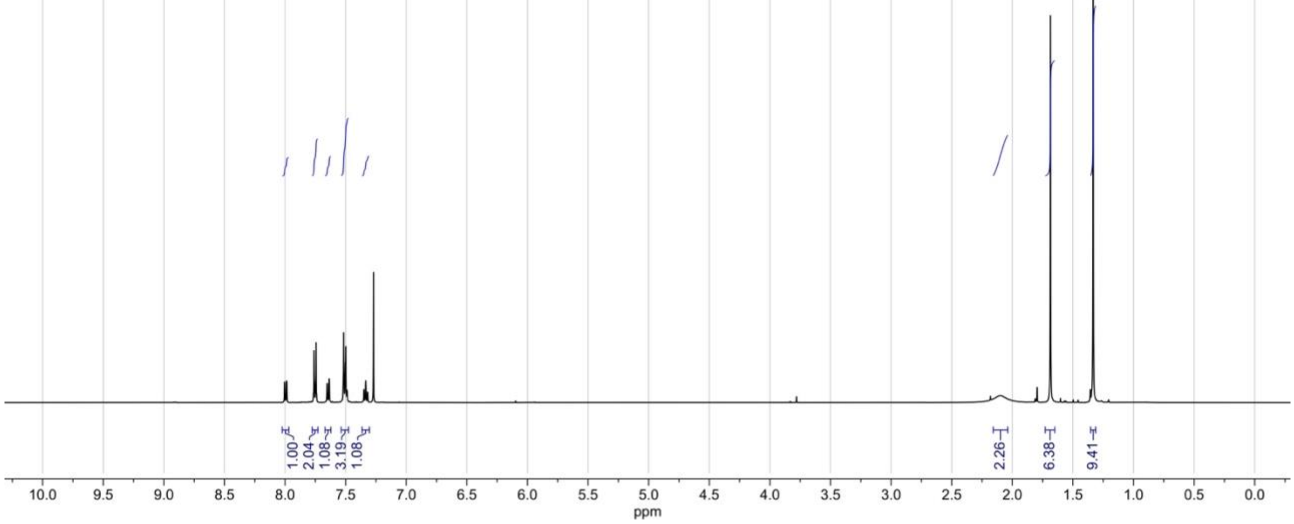

24
^{13}C NMR
(126 MHz, $CDCl_3$)

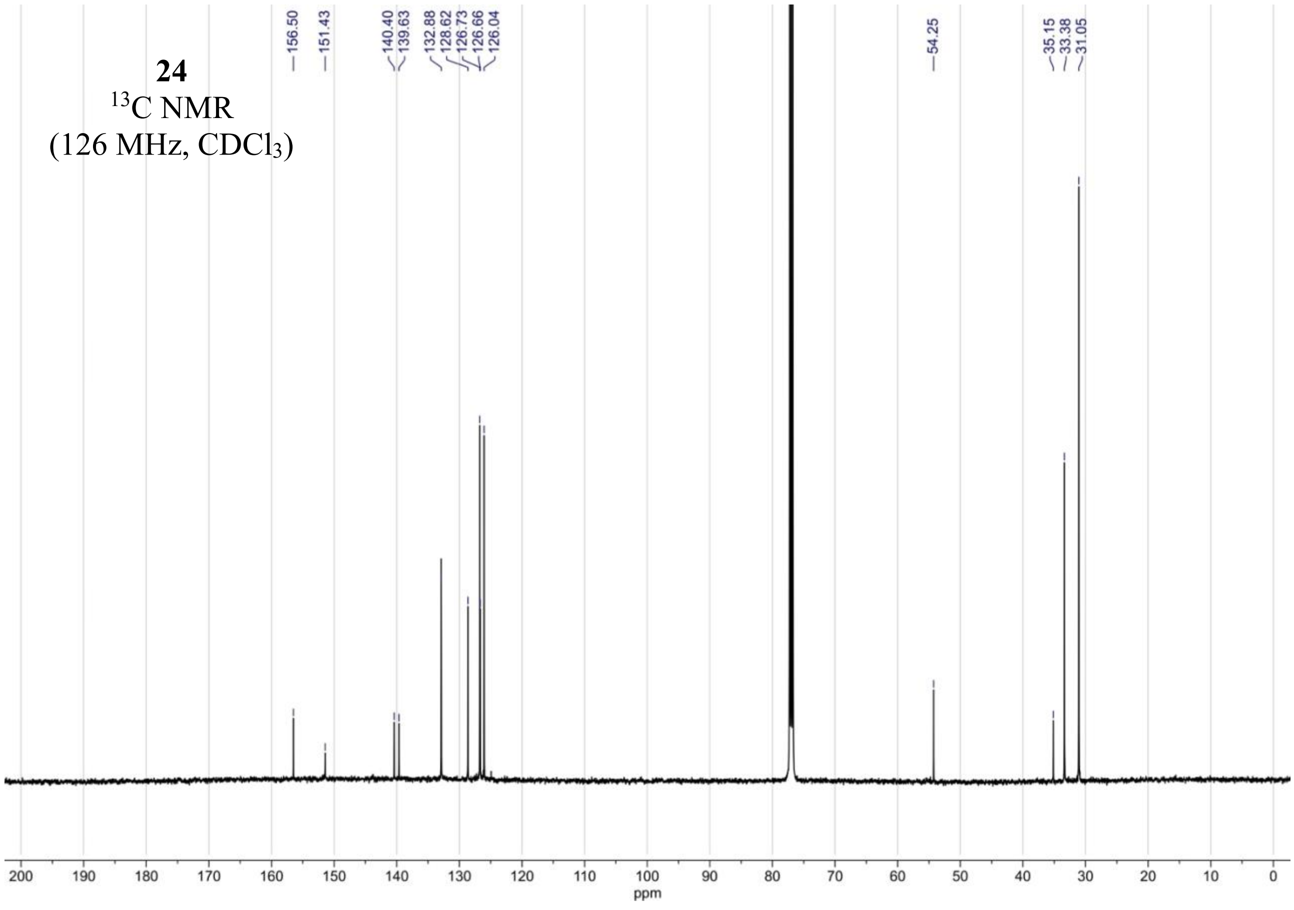

2-(2-((4-Methoxyphenyl)sulfonyl)phenyl)propan-2-amine 25

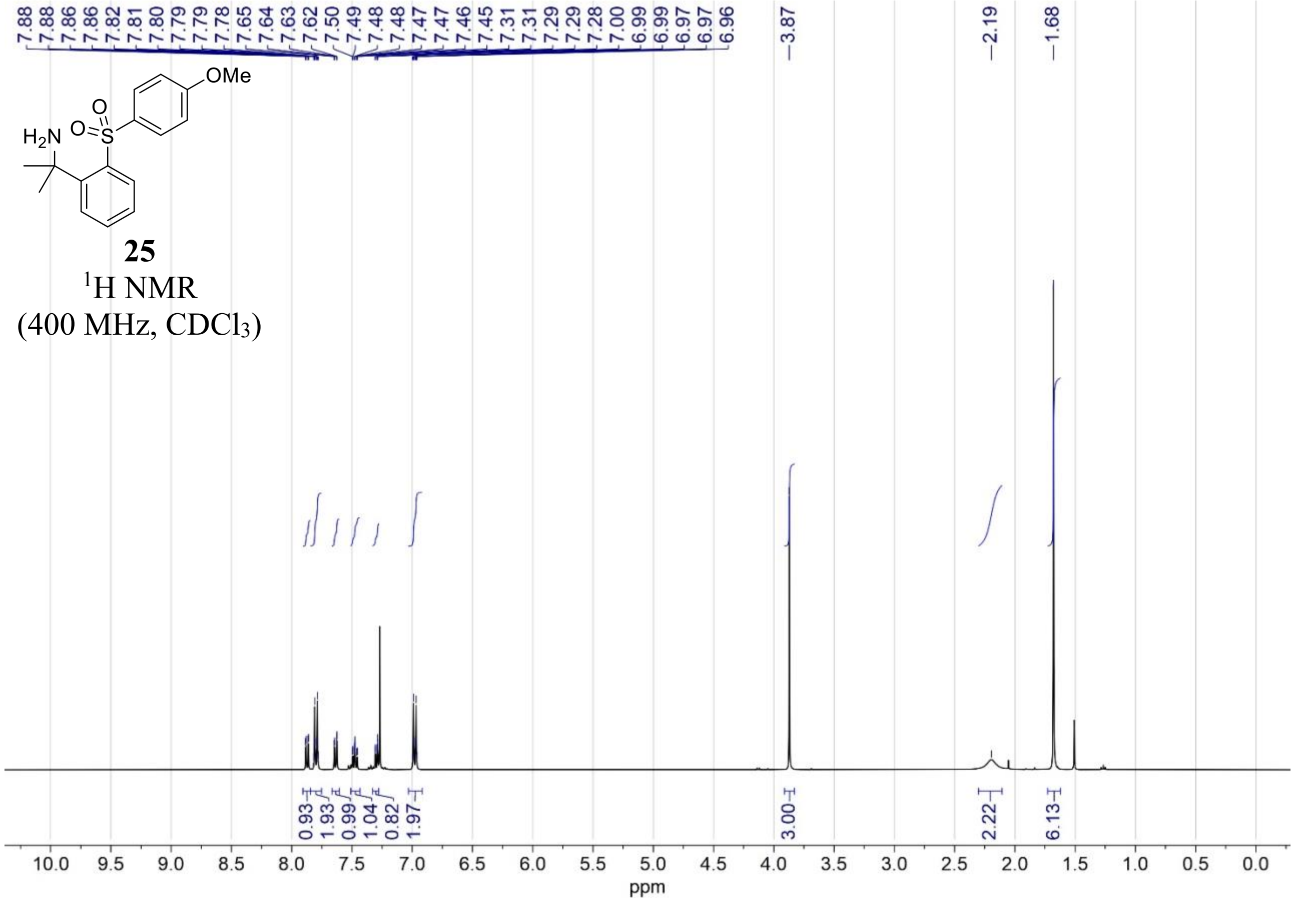

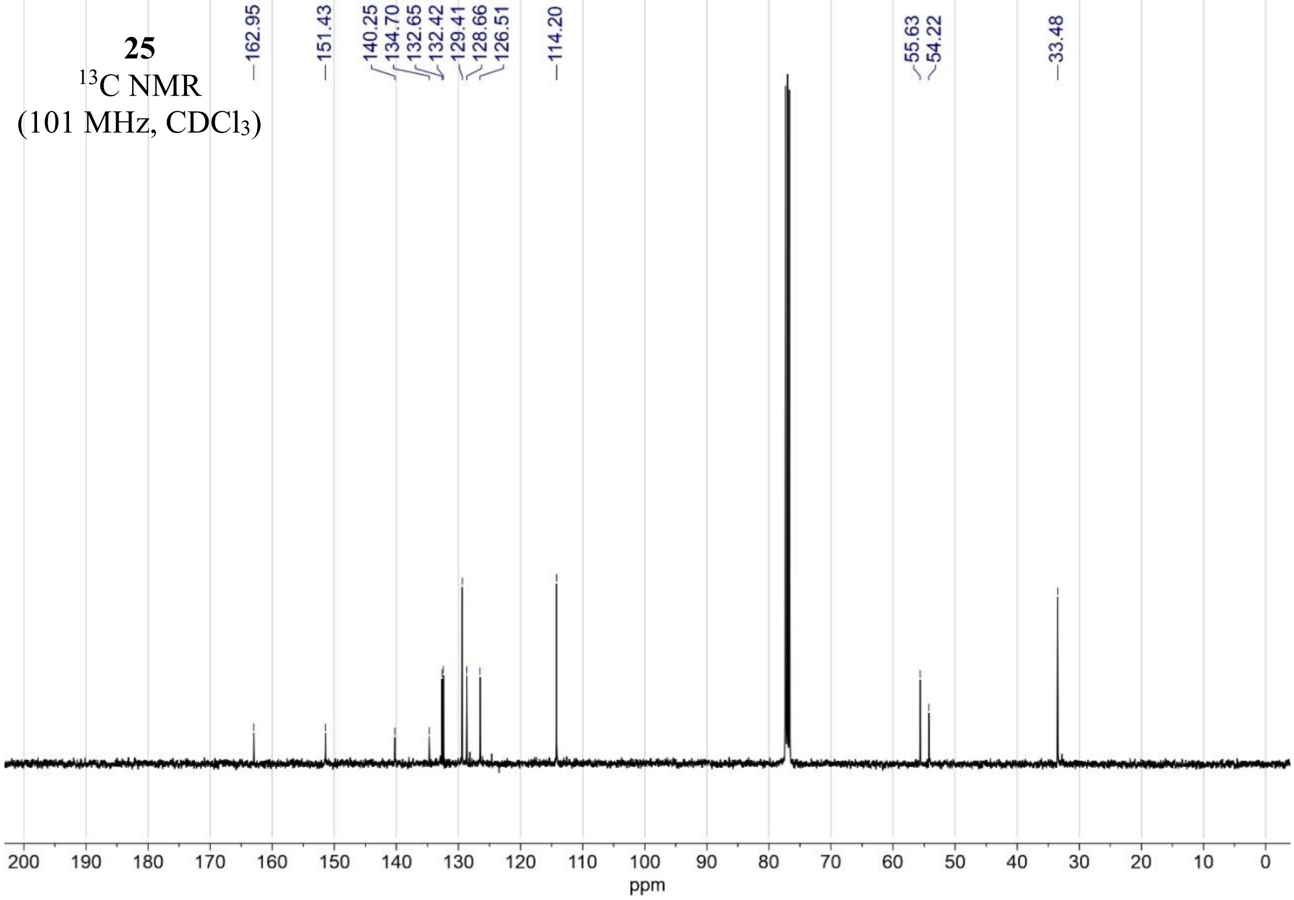

2-(2-((4-(Trifluoromethyl)phenyl)sulfonyl)phenyl)propan-2-amine 26

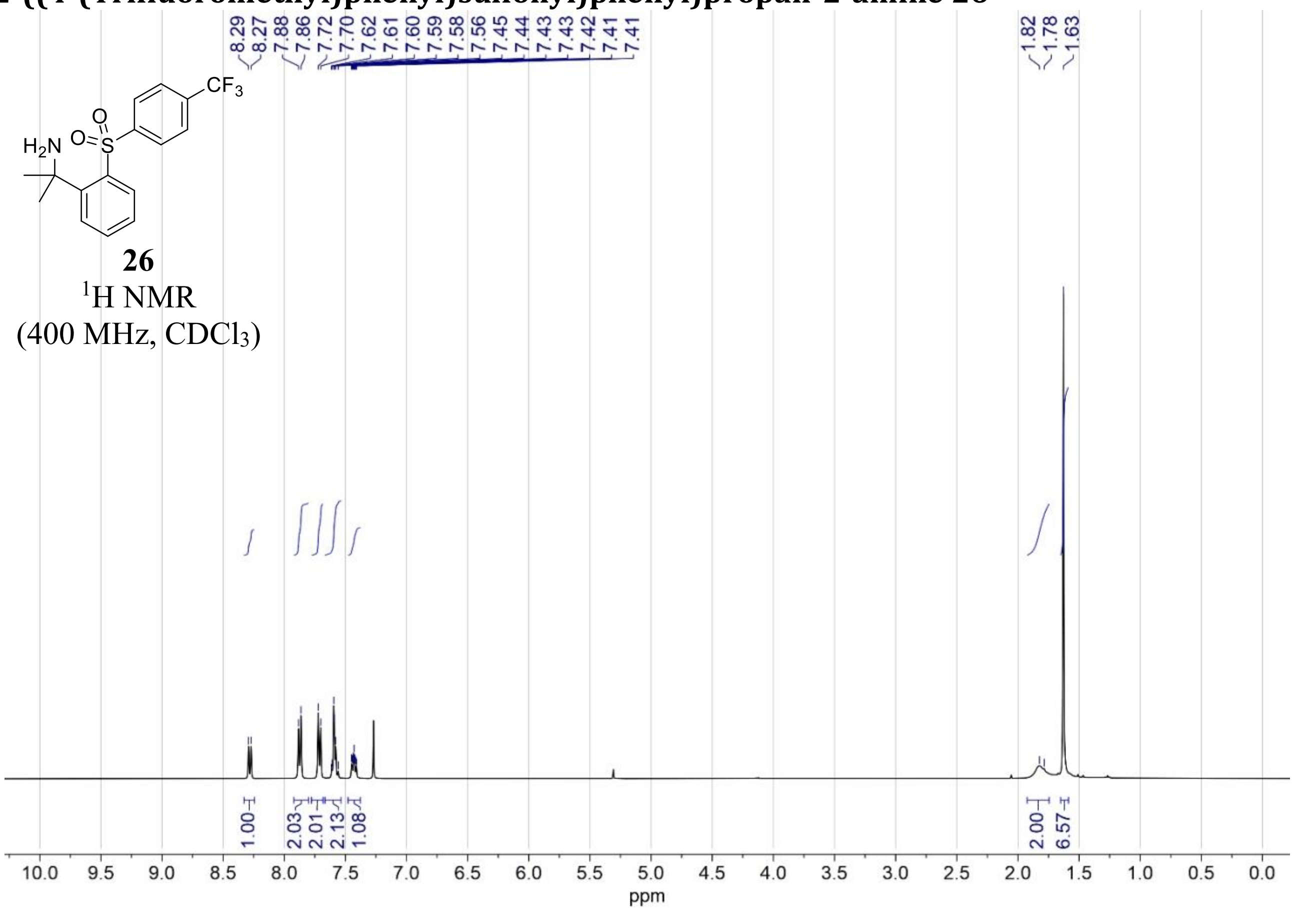

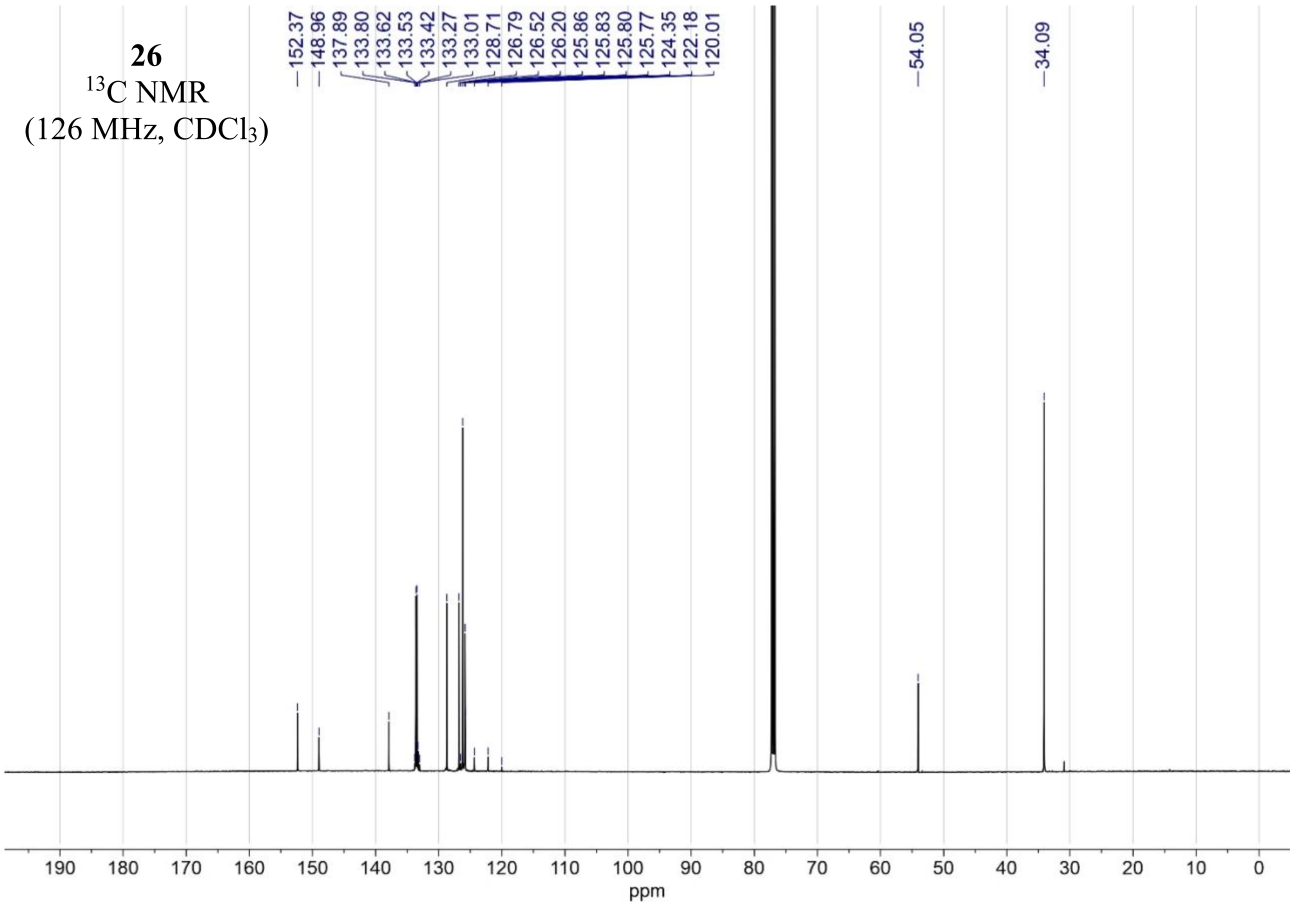

26
^{19}F NMR
(377 MHz, $CDCl_3$)

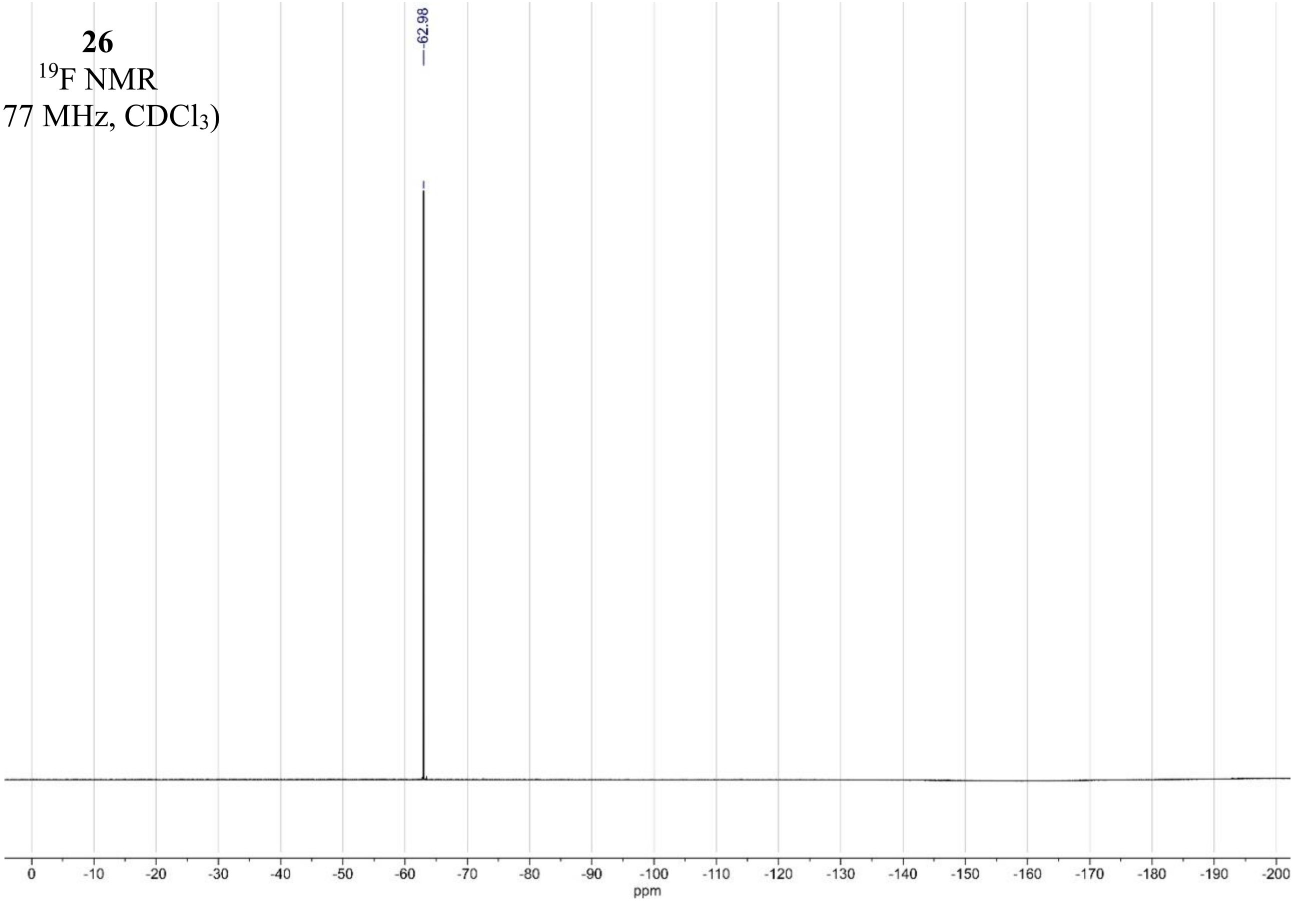

2-(2-((3,4-Dichlorophenyl)sulfonyl)phenyl)propan-2-amine 27

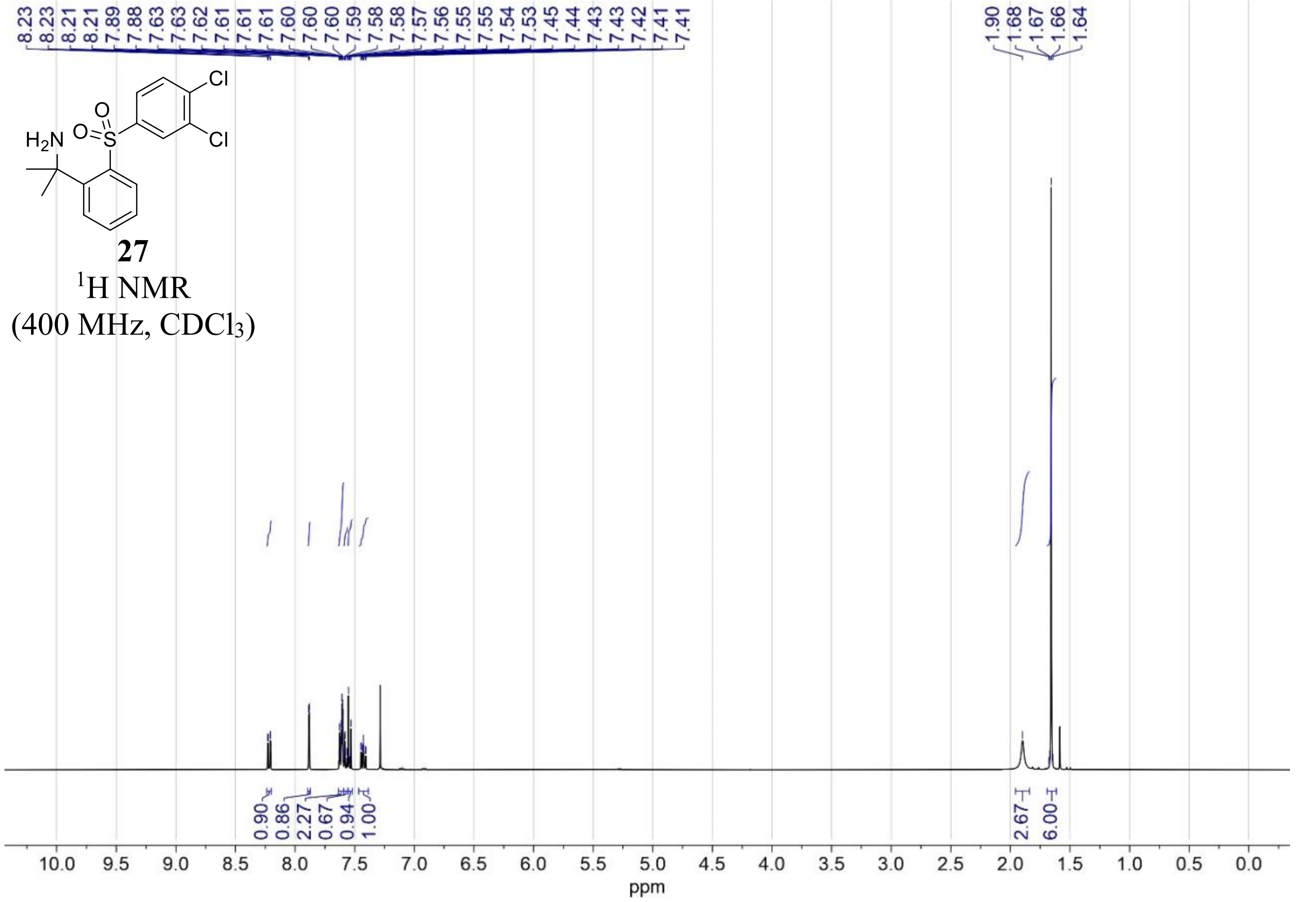

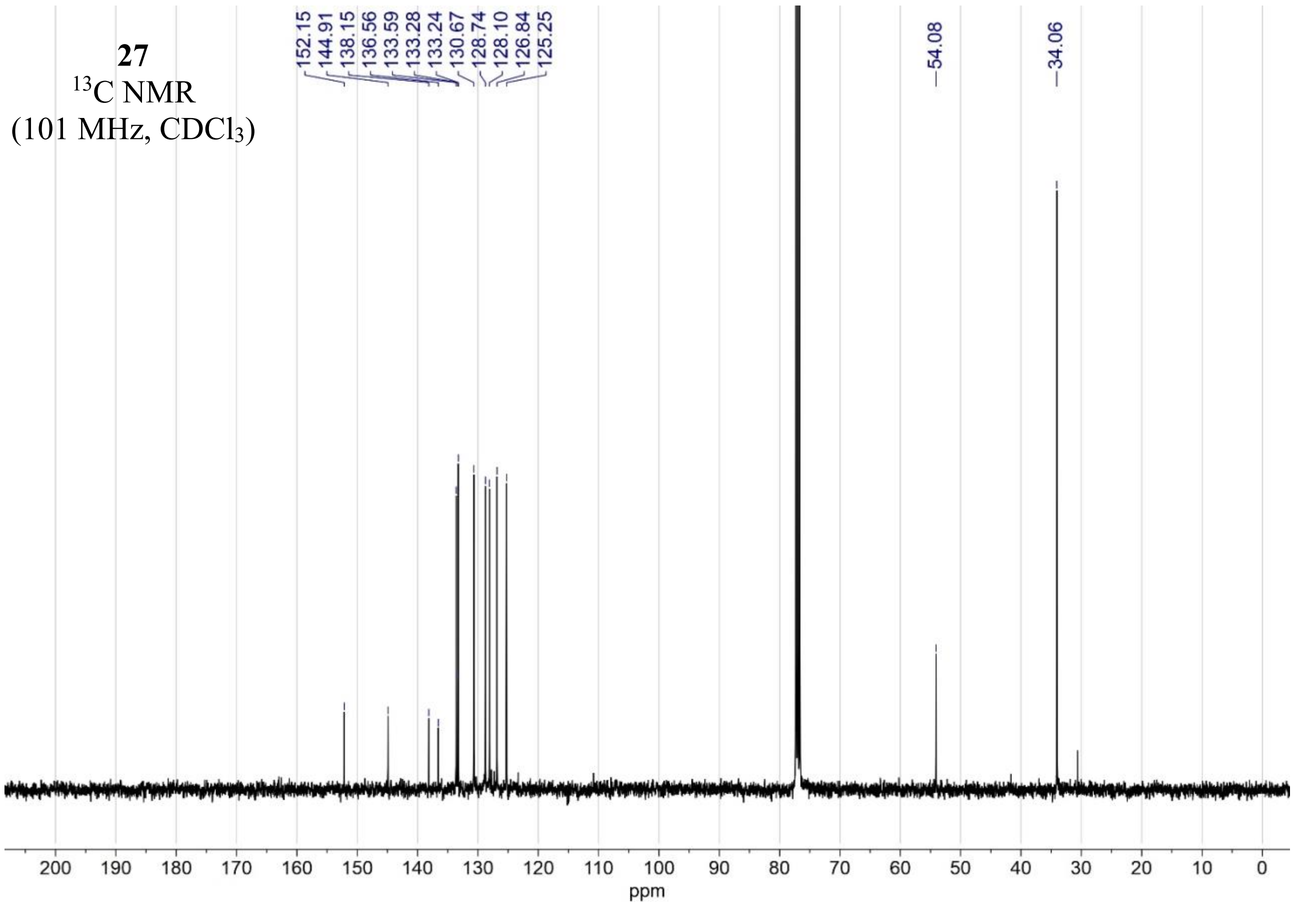

2-(2-((3,5-Bis(trifluoromethyl)phenyl)sulfonyl)phenyl)propan-2-amine 28

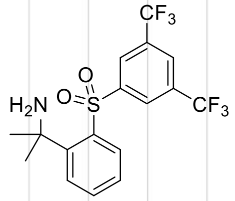

28
^{1}H NMR
(400 MHz, $CDCl_3$)

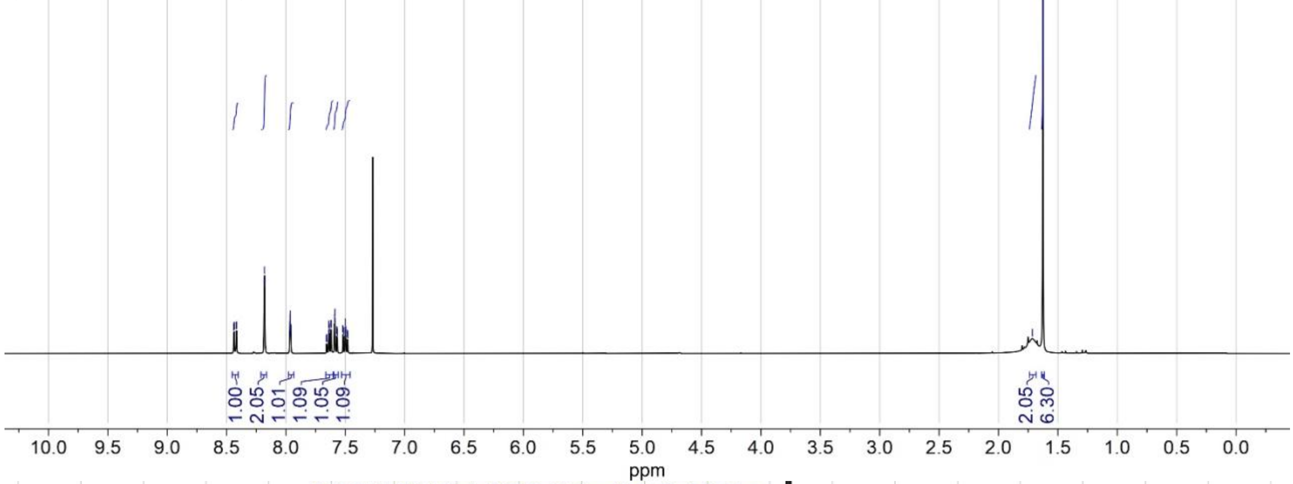

28
^{13}C NMR
(101 MHz, $CDCl_3$)

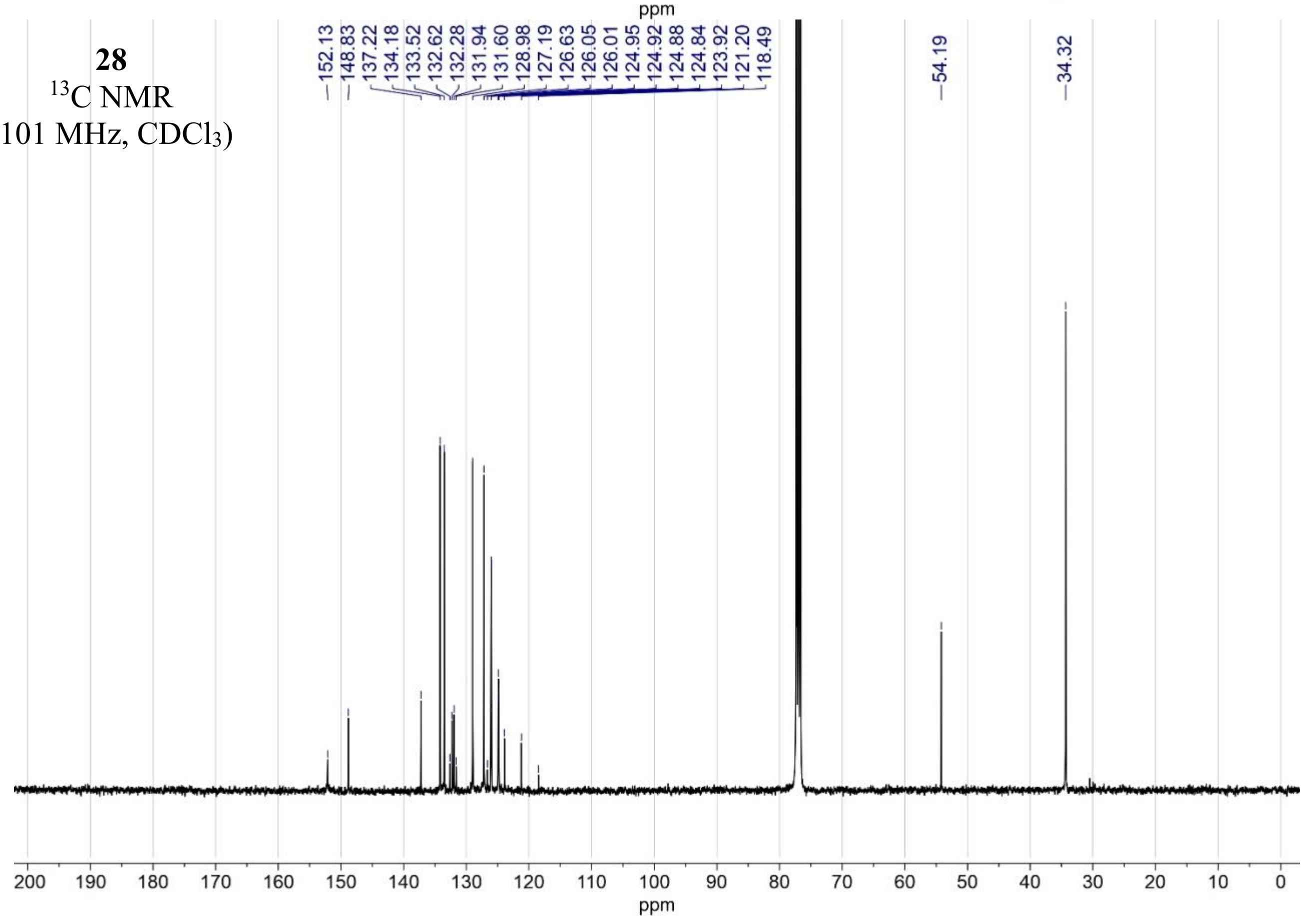

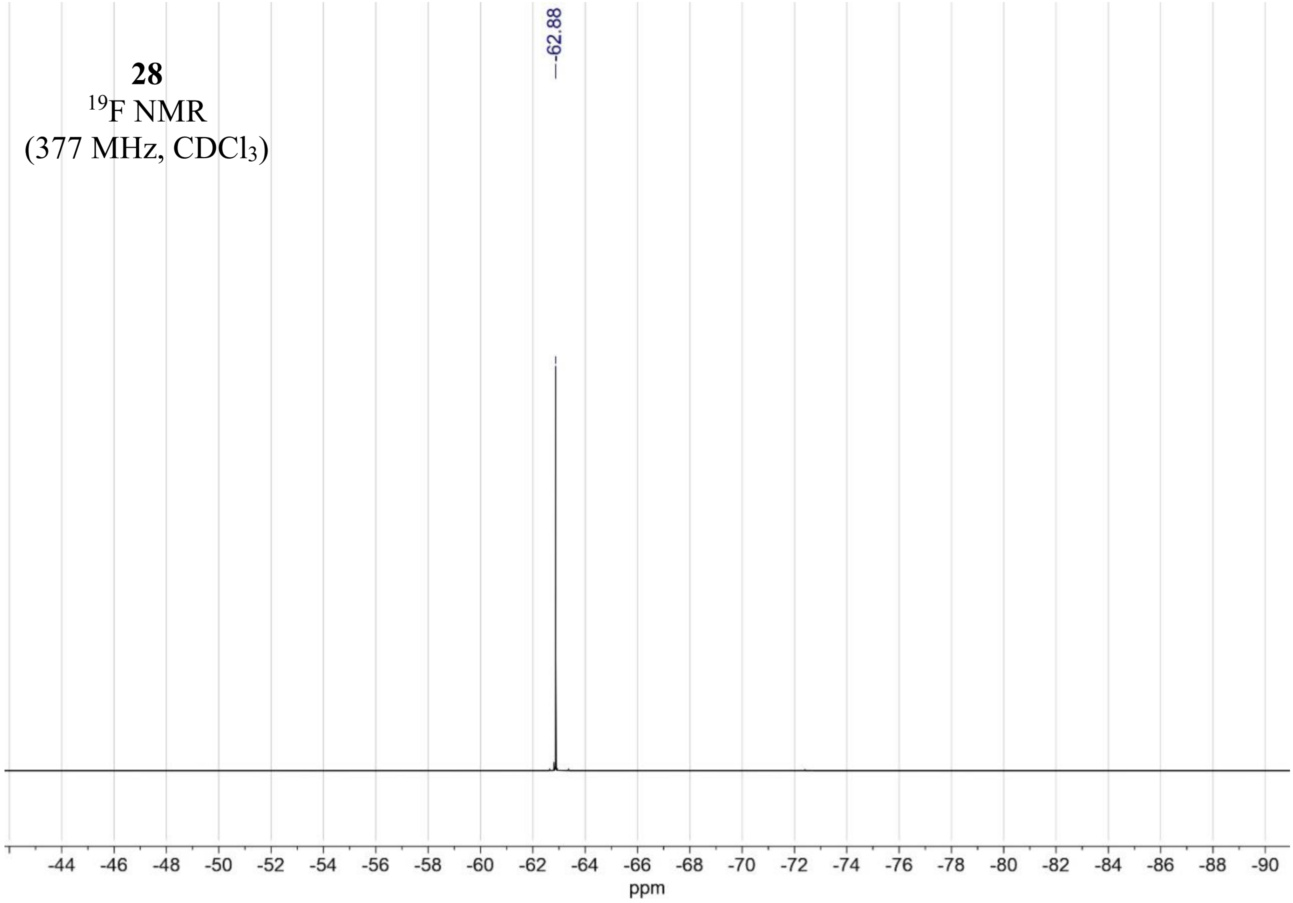

28
^{19}F NMR
(377 MHz, $CDCl_3$)
-62.88
-44 -46 -48 -50 -52 -54 -56 -58 -60 -62 -64 -66 -68 -70 -72 -74 -76 -78 -80 -82 -84 -86 -88 -90
ppm

X-Ray Crystallography

Data were collected using Agilent Xcalibur PX Ultra A [**8 9**, **17**, **21**] and Agilent Xcalibur 3 E [**10**] diffractometers, and the structures were solved by direct methods and refined by full-matrix least squares on F2 for all data using the OLEX2,[7] SHELXTL[8] and SHELX-2013[9] program systems.[10,11] The absolute structure of **9**, **10** and **17** were determined by use of the Flack parameter [x = 0.01(2), 0.05(3) and 0.006(16) respectively]. Crystallographic data and related CIFs for the structures **8**, **9**, **10**, **17** and **21** have been deposited with the joint Cambridge Crystallographic Data Centre and Fachinformationszentrum Karlsruhe Access Structures service and are available free of charge with the following deposition numbers: CCDC Deposition Numbers: 2416954-2416958.

X-Ray Crystallography Notes

The N–H hydrogen atoms in the structure of **8**, **9**, **10**, **17** and **21** were located from a ΔF map and refined freely subject to an N–H distance constraint of 0.90 Å.

The structure of **9** was found to contain two crystallographically independent molecules (**9** and **9**-B) in the asymmetric unit.

The structure of **10** was found to contain two crystallographically independent molecules (**10** and **10**-B) in the asymmetric unit.

An extinction correction was applied to the structure of **17** (EXTI 0.0150(18)) to account for the intensity changes associated with extinction from the large, selected crystal (0.17 x 0.29 x 0.67).

Crystal Structure: 8

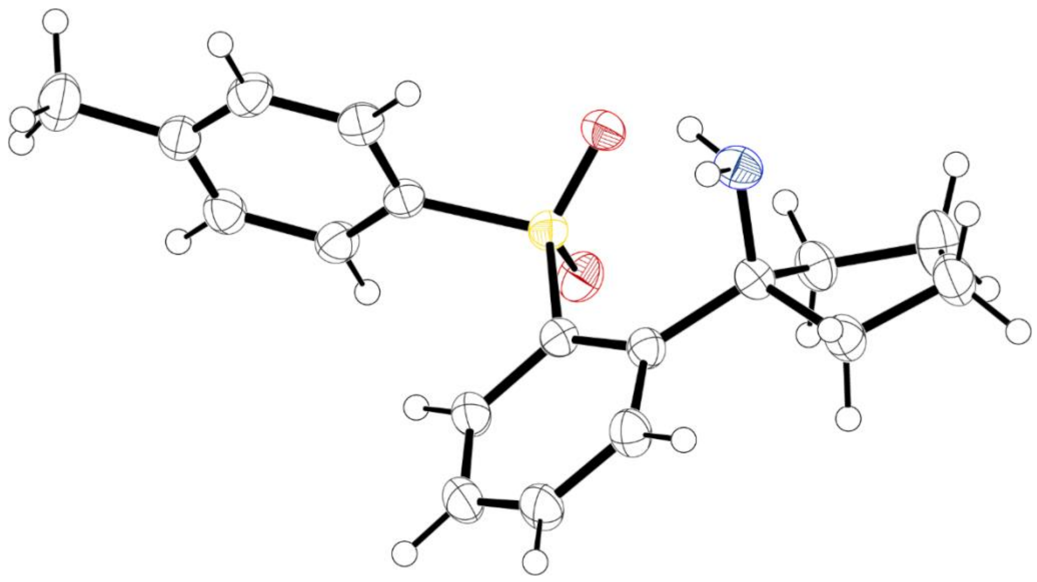

CCDC deposition number	2416954
Empirical formula	$C_{18}H_{21}NO_2S$
Formula weight	315.42
Temperature/K	174(1)
Crystal system	monoclinic
Space group	$P2_1/c$
a/Å	8.8204(2)
b/Å	17.4912(4)
c/Å	10.3266(3)
α/°	90
β/°	93.091(2)
γ/°	90
Volume/Å^3	1590.86(7)
Z	4
ρ_{calc}g/cm^3	1.317
μ/mm^{-1}	1.856
F(000)	672.0
Crystal size/mm^3	0.254 × 0.194 × 0.171
Radiation	Cu Kα (λ = 1.54184)
2Θ range for data collection/°	9.958 to 147.072
Index ranges	-10 ≤ h ≤ 10, -21 ≤ k ≤ 21, -11 ≤ l ≤ 12
Reflections collected	9366
Independent reflections	3149 [R_{int} = 0.0388, R_{sigma} = 0.0376]
Data/restraints/parameters	3149/2/208
Goodness-of-fit on F^2	1.037
Final R indexes [I>=2σ (I)]	R_1 = 0.0391, wR_2 = 0.0992
Final R indexes [all data]	R_1 = 0.0484, wR_2 = 0.1080
Largest diff. peak/hole / e Å^{-3}	0.30/-0.42

Crystal Structure: 9

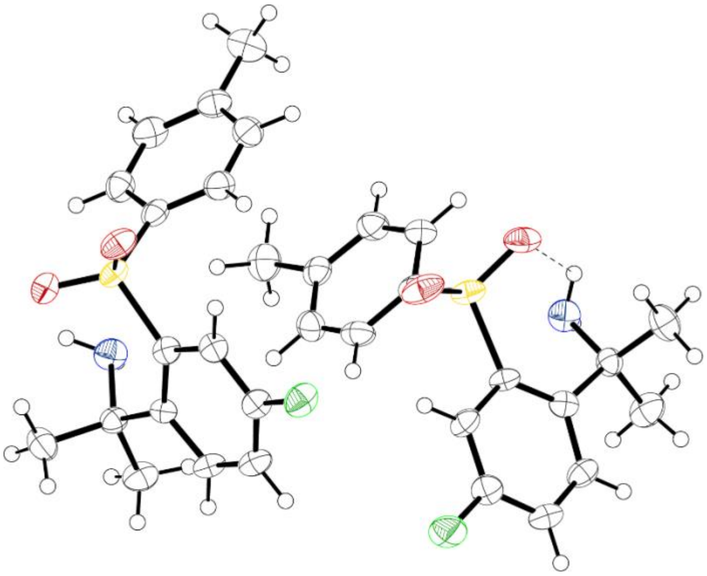

CCDC deposition number	2416955
Empirical formula	$C_{16}H_{18}NO_2FS$
Formula weight	307.37
Temperature/K	173(2)
Crystal system	orthorhombic
Space group	$Pna2_1$
a/Å	26.6268(6)
b/Å	8.17700(10)
c/Å	13.6282(3)
α/°	90
β/°	90
γ/°	90
Volume/Å^3	2967.23(10)
Z	8
ρ_{calc}g/cm^3	1.376
μ/mm^{-1}	2.076
F(000)	1296.0
Crystal size/mm^3	0.2 × 0.16 × 0.04
Radiation	Cu Kα (λ = 1.54184)
2Θ range for data collection/°	6.64 to 147.166
Index ranges	-31 ≤ h ≤ 29, -10 ≤ k ≤ 6, -16 ≤ l ≤ 11
Reflections collected	8349
Independent reflections	4328 [R_{int} = 0.0360, R_{sigma} = 0.0440]
Data/restraints/parameters	4328/5/401
Goodness-of-fit on F^2	1.035
Final R indexes [I>=2σ (I)]	R_1 = 0.0539, wR_2 = 0.1330
Final R indexes [all data]	R_1 = 0.0591, wR_2 = 0.1371
Largest diff. peak/hole / e Å^{-3}	0.94/-0.35
Flack parameter	0.01(2)

Crystal Structure: 10

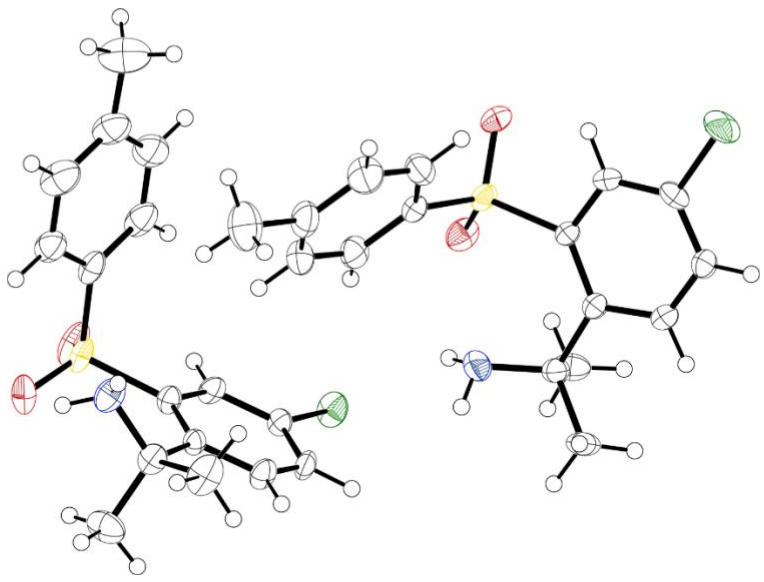

CCDC deposition number	2416956
Empirical formula	$C_{16}H_{18}NO_2SCl$
Formula weight	323.82
Temperature/K	172.95(10)
Crystal system	orthorhombic
Space group	$Pca2_1$
a/Å	15.9386(4)
b/Å	10.0214(3)
c/Å	19.7857(6)
α/°	90
β/°	90
γ/°	90
Volume/Å^3	3160.31(16)
Z	8
ρ_{calc}g/cm^3	1.361
μ/mm^{-1}	0.377
F(000)	1360.0
Crystal size/mm^3	0.352 × 0.132 × 0.063
Radiation	Mo Kα (λ = 0.71073)
2Θ range for data collection/°	4.802 to 56.458
Index ranges	-20 ≤ h ≤ 12, -13 ≤ k ≤ 7, -25 ≤ l ≤ 25
Reflections collected	9581
Independent reflections	6005 [R_{int} = 0.0244, R_{sigma} = 0.0414]
Data/restraints/parameters	6005/5/401
Goodness-of-fit on F^2	1.056
Final R indexes [I>=2σ (I)]	R_1 = 0.0368, wR_2 = 0.0744
Final R indexes [all data]	R_1 = 0.0454, wR_2 = 0.0793
Largest diff. peak/hole / e Å^{-3}	0.27/-0.27
Flack parameter	0.05(3)

Crystal Structure: 17

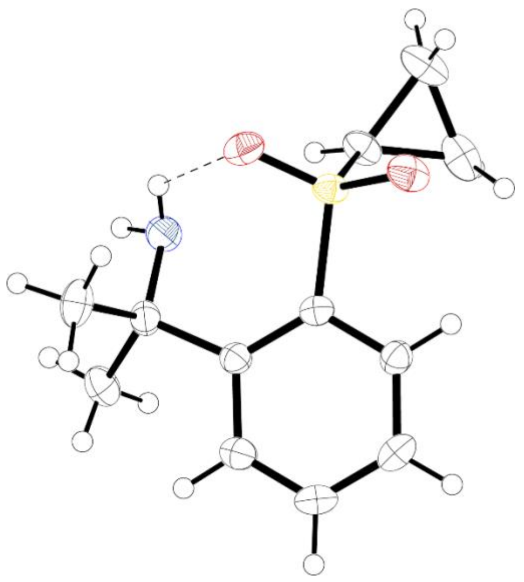

CCDC deposition number	2416957
Empirical formula	$C_{12}H_{17}NO_2S$
Formula weight	239.32
Temperature/K	173(2)
Crystal system	monoclinic
Space group	*P*2$_1$
a/Å	8.0035(3)
b/Å	9.4127(2)
c/Å	8.1768(3)
α/°	90
β/°	101.588(3)
γ/°	90
Volume/Å^3	603.44(3)
Z	2
ρ_{calc}g/cm^3	1.317
μ/mm^{-1}	2.267
F(000)	256.0
Crystal size/mm^3	0.673 × 0.292 × 0.172
Radiation	Cu Kα (λ = 1.54184)
2Θ range for data collection/°	11.046 to 146.716
Index ranges	-7 ≤ h ≤ 9, -11 ≤ k ≤ 11, -9 ≤ l ≤ 8
Reflections collected	3477
Independent reflections	2278 [R_{int} = 0.0202, R_{sigma} = 0.0302]
Data/restraints/parameters	2278/3/156
Goodness-of-fit on F^2	1.071
Final R indexes [I>=2σ (I)]	R_1 = 0.0294, wR_2 = 0.0773
Final R indexes [all data]	R_1 = 0.0309, wR_2 = 0.0785
Largest diff. peak/hole / e Å^{-3}	0.17/-0.23
Flack parameter	0.006(16)

Crystal Structure: 21

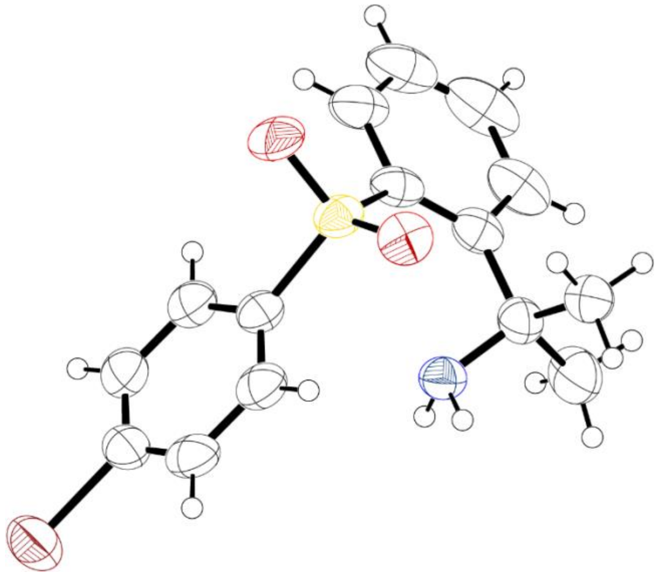

CCDC deposition number	2416958
Empirical formula	$C_{15}H_{16}BrNO_2S$
Formula weight	354.26
Temperature/K	173(2)
Crystal system	orthorhombic
Space group	*P*bca
a/Å	14.4036(5)
b/Å	7.06690(10)
c/Å	30.0137(8)
α/°	90
β/°	90
γ/°	90
Volume/Å^3	3055.06(14)
Z	8
ρ_{calc}g/cm^3	1.540
μ/mm^{-1}	4.952
F(000)	1440.0
Crystal size/mm^3	0.39 × 0.18 × 0.04
Radiation	Cu Kα (λ = 1.54184)
2Θ range for data collection/°	8.51 to 147.258
Index ranges	-16 ≤ h ≤ 11, -8 ≤ k ≤ 8, -36 ≤ l ≤ 25
Reflections collected	8097
Independent reflections	2986 [R_{int} = 0.0320, R_{sigma} = 0.0312]
Data/restraints/parameters	2986/2/191
Goodness-of-fit on F^2	1.120
Final R indexes [I>=2σ (I)]	R_1 = 0.0669, wR_2 = 0.1814
Final R indexes [all data]	R_1 = 0.0774, wR_2 = 0.1893
Largest diff. peak/hole / e Å^{-3}	1.13/-0.71

References

(1) Ma, B.; Lee, W.-C. A Modified Curtius Reaction: An Efficient and Simple Method for Direct Isolation of Free Amine. *Tetrahedron Lett.* **2010**, *51*, 385–386. https://doi.org/10.1016/j.tetlet.2009.11.038.

(2) Higham, J. I.; Ma, T.-K.; Bull, J. A. Dual Copper- and Aldehyde-Catalyzed Transient C–H Sulfonylation of Benzylamines. *Org. Lett.* **2023**, *25*, 5285–5290. https://doi.org/10.1021/acs.orglett.3c01783.

(3) Wang, R.; Gregg, B. T.; Zhang, W.; Golden, K. C.; Quinn, J. F.; Cui, P.; Tymoshenko, D. O. Rapid $Ti(Oi\text{-}Pr)_4$ Facilitated Synthesis of *α*, *α*, *α*-Trisubstituted Primary Amines by the Addition of Grignard Reagents to Nitriles under Microwave Heating Conditions. *Tetrahedron Lett.* **2009**, *50*, 7070–7073. https://doi.org/10.1016/j.tetlet.2009.09.178.

(4) Vasu, D.; Arriba, A. L. F. de; Leitch, J. A.; Gombert, A. de; Dixon, D. J. Primary *α*-Tertiary Amine Synthesis via *α*-C–H Functionalization. *Chem. Sci.* **2019**, *10*, 3401–3407. https://doi.org/10.1039/C8SC05164J.

(5) Kovacic, P.; Gormish, J. F.; Hopper, R. J.; Knapczyk, J. W. Chemistry of N-Halamines. XI. Side-Chain Amination of Aryldialkylmethines with Trichloramine-Aluminum Chloride-Tert-Butyl Bromide. *J. Org. Chem.* **1968**, *33*, 4515–4520. https://doi.org/10.1021/jo01276a047.

(6) Limanto, J.; Dorner, B.; Devine, P. Non-Cryogenic $CeCl_3$-Promoted Double Additions of Methyllithium and *n*-Butyllithium to Unsaturated Nitriles. *Synthesis* **2006**, *2006*, 4143–4150. https://doi.org/10.1055/s-2006-950362.

(7) Dolomanov, O. V.; Bourhis, L. J.; Gildea, R. J.; Howard, J. A. K.; Puschmann, H. OLEX2: A Complete Structure Solution, Refinement and Analysis Program. *J. Appl. Crystallogr.* **2009**, *42* (2), 339–341. https://doi.org/10.1107/S0021889808042726.

(8) Sheldrick, G. M. SHELXTL, V5.1; Bruker AXS: Madison, WI, **1998**.

(9) G.M. Sheldrick. Crystal Structure Refinement with SHELXL. *Acta Cryst.*, **2015**, *C71*, 3-8. https://doi.org/10.1107/S2053229614024218.

(10) Spek A.L. PLATON, A Multipurpose Crystallographic Tool, Utrecht University, Utrecht, (2003, 2009), The Netherlands.

(11) Spek, A. L. PLATON SQUEEZE: A Tool for the Calculation of the Disordered Solvent Contribution to the Calculated Structure Factors. *Acta Crystallogr., Sect. C: Struct. Chem.* **2015**, *C71* , 9–18. https://doi.org/10.1107/S2053229614024929.
